# Chemical Pollutant Exposure in Neurodevelopmental Disorders: Integrating Toxicogenomic and Transcriptomic Evidence to Elucidate Shared Biological Mechanisms and Developmental Signatures

**DOI:** 10.3390/toxics13040282

**Published:** 2025-04-08

**Authors:** Xuping Gao, Xinyue Wang, Xiangyu Zheng, Yilu Zhao, Ning Wang, Suhua Chang, Li Yang

**Affiliations:** 1 Peking University Sixth Hospital, Peking University Institute of Mental Health, NHC Key Laboratory of Mental Health (Peking University), National Clinical Research Center for Mental Disorders (Peking University Sixth Hospital), No. 51 HuayuanBei Road, Beijing 100191, China; xuping@bjmu.edu.cn (X.G.);; 2 Department of Public Health and Preventive Medicine, School of Medicine, Jinan University, No. 601 Huangpu Road West, Guangzhou 510632, China; 3 Affiliated Mental Health Center & Hangzhou Seventh People’s Hospital, Zhejiang University School of Medicine, No. 305 Tianmushan Street, Hangzhou 310007, China; 4 Department of Clinical Psychology, Beijing Anzhen Hospital, Capital Medical University, No. 2 Anzhen Road, Beijing 100029, China

**Keywords:** chemical pollution, exposome, neurodevelopmental disorders, Comparative Toxicogenomics Database (CTD), developmental transcriptome, xenobiotic metabolic process

## Abstract

Rapid industrialization has introduced a range of chemicals into the environment, posing significant risks to fetal and child brain development. Using the Comparative Toxicogenomics Database (CTD), we constructed chemical exposome frameworks for seven neurodevelopmental disorders (NDDs) and identified chemical pollutants of epidemiological concern, including air pollutants (n = 8), toxic elements (n = 14), pesticides and related compounds (n = 18), synthetic organic chemicals (n = 16), and solvents (n = 5). Gene set enrichment analysis validated and revealed significant toxicogenomic associations between these chemical pollutants and NDDs, including autism spectrum disorder (ASD) (12 pollutants, proportional reporting ratio (PRR) 3.56–7.21) and intellectual disability (ID) (9 pollutants, PRR 3.13–5.59). Functional annotation of pollutant-specific gene sets highlighted shared biological processes, such as metabolic processes (e.g., xenobiotic metabolic process, xenobiotic catabolic process, and cytochrome P450 pathway) for ASD and cognitive processes (e.g., cognition, social behavior, and synapse assembly) for ID (Bonferroni-corrected *p*-values < 0.05). Time trajectory analysis of developmental transcriptomic data from the BrainSpan database for ASD (275 genes) and ID (93 genes) revealed three distinct expression patterns of chemical-pollutant-associated genes—higher prenatal, postnatal, and perinatal expression—indicating common and divergent underlying mechanisms across critical windows of chemical pollutant exposure.

## 1. Introduction

The rapid industrialization and intensification of human activities have introduced a wide range of chemicals into the environment. Fetuses and children are particularly sensitive to these chemicals due to their actively developing brains and immature detoxification systems, making them more susceptible than adults [1,2]. Over time, exposure to hazardous chemicals can disrupt the intricate gene–environment interplay, potentially altering stable brain development and increasing the risk of neurodevelopmental disorders (NDDs) [3]. These disorders, characterized by delays or impairments in cognitive, social, and motor development, lead to deficits in adaptive behavior [4]. According to the latest 2023 report from the World Health Organization and the United Nations Children’s Fund, developmental disabilities currently affect over 316.8 million children worldwide, constituting a significant global health challenge [5].

NDDs exhibit heterogeneous etiology and broadly include autism spectrum disorder (ASD), intellectual disability (ID), attention deficit hyperactivity disorder (ADHD), and language disorders, among others [4,6]. Despite their high prevalence, understanding the etiology of NDDs remains complex, as it involves challenges such as delineating the genetic components, identifying environmental risk factors, and elucidating the precise mechanisms through which of these factors interact to contribute to the development of these disorders [7]. Advances in next-generation sequencing (NGS) and mass spectrometry (MS) technologies have boosted etiological research into the omics era, facilitating more comprehensive analyses of both genetic and non-genetic factors. Complementing genomics, the concept of the exposome emphasizes the systematic identification and evaluation of environmental exposures and their impacts on health [8]. Integrating the chemical exposome, particularly chemical pollutants, is essential for elucidating the precise mechanisms underlying NDDs, as these exposures could disrupt normal developmental processes from the embryonic stage through childhood, resulting in various adverse neurodevelopmental outcomes [9,10,11].

Increasing attention has been directed toward how pollutant exposures shape the developmental trajectory by identifying genes and pathways associated with the type and severity of exposures [12]. In vivo studies have demonstrated that chronic low-level exposure to organophosphate (OP) pesticides can alter gene expression related to psychological disorders, disrupt neurotransmitter systems (e.g., glutamate, dopamine, and serotonin), and impair cognitive function and behavior [13,14]. Similarly, long-term gestational exposure to air pollution in mice induces transcriptomic changes that disrupt cytokine production, IL-17a signaling, and dopamine degradation, leading to behavioral and phenotypic alterations [15,16]. Manganese (Mn) exposure has been found to upregulate genes related to apoptosis and DNA damage repair, consequently disrupting the MAPK pathway in zebrafish larvae [17]. However, current studies often lack an omics-based approach to comprehensively characterize chemical pollutant exposures and typically focus on a single category or substance. Research on the interactions between genes and pollutants in the etiology of NDDs remains limited, partly due to the substantial financial resources and long-term follow-up required, which constrain both sample size and analytical depth.

A comprehensive investigation of the pollutants and pathways implicated in NDDs necessitates integrating evidence from multiple model systems and human datasets. The Comparative Toxicogenomics Database (CTD) [18], supported by the National Institute of Environmental Health Sciences (NIEHS) of the United States, provides a valuable resource for such analyses. The CTD encompasses millions of curated relationships among genes, chemicals, and diseases, offering an opportunity to characterize the evidence-based chemical exposome and assess pollutant-related gene interactions and functional profiles associated with NDDs. To strengthen the statistical reliability of gene interactions and reduce random associations, we employ gene set enrichment analysis (GSEA) based on hypergeometric testing [19]. Leveraging these resources and methodologies, our study aims to: (1) identify potential pollutant mixtures within the chemical exposome of NDDs; (2) cross-verify and extend associations identified by CTD using complementary datasets; (3) analyze shared biological processes among mixed chemical pollutants; and (4) examine the developmental transcriptome signatures of the identified pollutant–disorder gene sets.

## 2. Materials and Methods

### 2.1. Neurodevelopmental Disorders in the Comparative Toxicogenomics Database

We utilized the CTD to identify NDDs with curated evidence of associations with various chemicals. The CTD compiles chemical–disease associations curated from published literature and infers potential relationships through chemical–gene and gene–disease interactions [18]. A systematic screening of all categories and hierarchical descendants of NDDs was conducted to identify those with detailed information on associated chemicals, genes, phenotypes, and exposure references (https://ctdbase.org, accessed on 31 October 2024), as described in Supplementary Methods Section S1.1. Chemical–disease interactions were collected, including both curated marker/mechanism associations and therapeutic associations. Eligible data were identified for disorders classified under Medical Subject Heading (MeSH) terms, including attention deficit disorder with hyperactivity (MeSH Identifier: D001289), autism spectrum disorder (D000067877), autistic disorder (D001321), developmental disabilities (D002658), intellectual disability (D008607), learning disabilities (D007859), and motor skills disorders (D019957). Language disorders and dyslexia were excluded due to insufficient chemical–disease associations (fewer than three). The strength of chemical–disease associations was quantified using inference scores, calculated as the logarithm of the product of two common-neighbor statistics, which assess the connectivity of the chemical, disease, and genes involved [20] (Supplementary Methods Section S1.2).

### 2.2. Chemical Pollutants from CTD and Independently Curated Datasets

Based on the integrated classification of a MeSH chemical subset, we screened chemical pollutants among the chemicals associated with NDDs in the CTD. Additionally, an evidence-based approach was used to collect meta-analytic pollutant–disorder associations, supplemented by a public-health-oriented strategy to identify additional pollutants potentially associated with NDDs. The two-step selection process resulted in the inclusion of 69 chemical pollutants, with 48 identified based on existing evidence and 21 added as chemicals of public health significance [3,21,22,23] (Supplementary Methods Sections S1.3). These chemical pollutants represent diverse classes, enabling comprehensive analyses, including: (1) air pollutants, such as particulate matter (PM), nitrogen oxides (NOx), sulfur oxides (SOx), carbon monoxide (CO), ozone (O_3_), polycyclic aromatic hydrocarbons (PAHs), phenanthrene, and pyrene; (2) toxic and trace elements, such as lead (Pb), mercury (Hg), cadmium (Cd), manganese (Mn), antimony (Sb), barium (Ba), cobalt (Co), nickel (Ni), zinc (Zn), copper (Cu), iron (Fe), aluminum (Al), lithium (Li), magnesium (Mg), and arsenic (As); (3) pesticides and related compounds, such as organochlorine pesticides, OPs, fungicides, herbicides, insecticides, carbamates, pyrethrins, hexachlorobenzene (HCB), glyphosate, N,N-diethyl-3-methylbenzamide (DEET), 4,4′-dichlorodiphenyltrichloroethane (DDT), dichlorodiphenyl dichloroethylene (DDE), parathion, methyl parathion, dieldrin, aldrin, and triclosan; (4) synthetic organic chemicals, such as per- and poly-fluoroalkyl substances (PFASs), perfluorooctanoic acid (PFOA), perfluorooctane sulfonic acid (PFOS), perfluorodecanoic acid (PFDA), perfluoro-n-nonanoic acid (PFNA), perfluorohexanesulfonic acid (PFHxS), polychlorinated biphenyls (PCBs), halogenated diphenyl ethers (HDPEs), dibutyl phthalate (DBP), diethylhexyl phthalate (DEHP), diethyl phthalate (DEP), bisphenol A (BPA), bisphenol B (BPB), bisphenol S (BPS), oxybenzone, plastics, and microplastics; (5) solvents, such as chloroform, methylene chloride, tetrachloroethylene, trichloroethylene, and carbon tetrachloride. To evaluate the comprehensiveness and consistency of the selection, we assessed the overlap between pollutants identified through CTD chemical–disease interactions and those curated independently of the CTD (Supplementary Methods Section S1.4).

### 2.3. Gene Sets for Neurodevelopmental Disorders and Chemical Pollutants

To generate unique gene sets for NDDs, we retrieved genes with curated associations (marker/mechanism and/or therapeutic) for each disorder: attention deficit disorder with hyperactivity (n = 22), ASD (n = 359), autistic disorder (n = 257), developmental disabilities (n = 31), ID (n = 139), learning disabilities (n = 33), and motor skills disorders (n = 13). For pollutants, we extracted genes with chemical–gene or chemical–protein interaction values exceeding 1% of the maximum interaction value for each chemical. Corresponding gene sets were obtained from the CTD (https://ctdbase.org, accessed on 12 November 2024), as detailed in Supplementary Methods Section S1.5. Genome annotation data for Homo sapiens (GRCh38.p14) were accessed from the NCBI Genome database, with pseudo-genes excluded, yielding a final background reference of 42,854 unique genes. All identified gene sets were then mapped to this background reference to ensure restriction to human-specific genes for downstream analyses (Supplementary Methods Section S1.6).

### 2.4. Enrichment Analysis of Chemical Pollutants in Neurodevelopmental Disorders

Gene set enrichment analysis (GSEA) was performed using 2 × 2 contingency tables for genes associated with each pollutant and each neurodevelopmental disorder. Expected frequencies for each table were estimated to determine the appropriate statistical tests. Standard chi-square tests were applied when expected frequencies in all cells were ≥5; Yates’ continuity correction was used when expected frequencies were <5 but >1; and Fisher’s exact tests were employed when any cell had an expected frequency ≤1. For each neurodevelopmental disorder, 69 multiple comparisons were conducted, and the Bonferroni correction was applied to account for multiple testing. A corrected *p*-value of less than 0.05 was considered statistically significant. To minimize false-positive results, contingency tables with any cell having an expected frequency ≤1 were excluded from further analysis. Fisher’s exact test was used to confirm the pollutant–disorder gene enrichments identified by the Standard chi-square test. Additionally, the proportional reporting ratio (PRR) with 95% confidence interval (95% CI) was calculated to evaluate the direction and magnitude of enrichment (i.e., more overlapping genes than expected by chance) or depletion (i.e., fewer overlapping genes than expected by chance). A PRR > 1 indicates enrichment, and PRR = 1 indicates no deviation from expectation.

### 2.5. Cross-Validation of Enrichment Analysis

To assess the likelihood of false-positive associations in enrichment analyses, we performed cross-validation using randomized testing for each neurodevelopmental disorder. Specifically, 1000 pseudo-chemical gene sets of varying sizes (50, 100, 250, 500, 1000, 2500, 5000, and 10,000 genes) were generated to reflect the range of pollutant-associated gene counts observed in our dataset. Each randomized gene set was tested for enrichment against disorder-specific gene sets using Fisher’s exact test, with false-positive results defined by unadjusted *p*-values < 0.01. The frequency of significant results across these random tests provided an estimate of the probability of false-positive findings occurring by chance. The distribution of observed *p*-values from the randomized tests was further visualized using density plots to characterize patterns of random associations.

### 2.6. Functional Annotation of Pollutant–Disorder Gene Sets

A prioritized list of pollutants was derived from GSEA results, integrating data from independently curated datasets and CTD findings. The intersection of chemical pollutant and disorder gene sets was calculated to generate pollutant–disorder gene sets, excluding NDD genes not present in any chemical pollutant gene sets. The complete list of pollutant–disorder gene sets is provided in Supplementary Methods Section S1.7. To investigate the biological functions of these gene sets, Gene Ontology (GO) term annotation was performed within the *biological process* category, using “Homo sapiens” as the reference background. The overlaps between pollutant–disorder gene sets were visualized using a Sankey diagram to highlight the most significant biological processes. The DisGeNET database was then utilized to assess the associations of the integrated pollutant–disorder gene sets with various diseases, offering insights into their broader health implications [24]. Furthermore, KEGG pathway annotation was conducted to identify key biological pathways potentially affected by the integrated pollutant–disorder gene sets. Functional annotations were performed using DAVID tools [25,26], hosted by the National Institutes of Health, to ensure rigor and reproducibility throughout the analysis.

### 2.7. Developmental Transcriptome Signatures of Pollutant–Disorder Gene Sets

To broadly cover critical windows of human brain development, we utilized the imputed BrainSpan developmental transcriptome dataset by Pei et al. [27], generated from 57 developing and adult postmortem brains of clinically unremarkable donors, spanning ages from 12 postconceptual weeks (pcw) to 40 years (yrs) [28]. The imputed gene expression matrix was downloaded from GitHub (https://github.com/bsml320/BrainSpan, accessed on 20 November 2023). For each gene, we fitted a multivariable linear model (expression ~ age + sex + brain region) to estimate the effect of age on gene expression [29]. Genes were ranked by t-statistics, and the top 5% with the highest expression and the bottom 5% with the lowest expression were selected to define temporal-specific gene groups. Fisher’s exact test was used to assess the association between the pollutant–disorder gene sets and these temporal-specific expression groups, with odds ratios (ORs) > 1 indicating a positive correlation and ORs < 1 indicating a negative correlation. Principal component analysis (PCA) was performed to explore the relationship between pollutant–disorder gene sets and brain development, while trajectory analysis identified clusters of gene expression patterns within the pollutant–disorder gene sets [30]. For the clustered pollutant–disorder genes, GO term annotation was used to characterize the key pollutant-related biological processes associated with temporal-specific expression patterns. Detailed analysis methods can be found in Supplementary Methods Section S1.8.

## 3. Results

### 3.1. Chemical Pollutants in the Chemical Exposome of Neurodevelopmental Disorders

**Figure 1:**
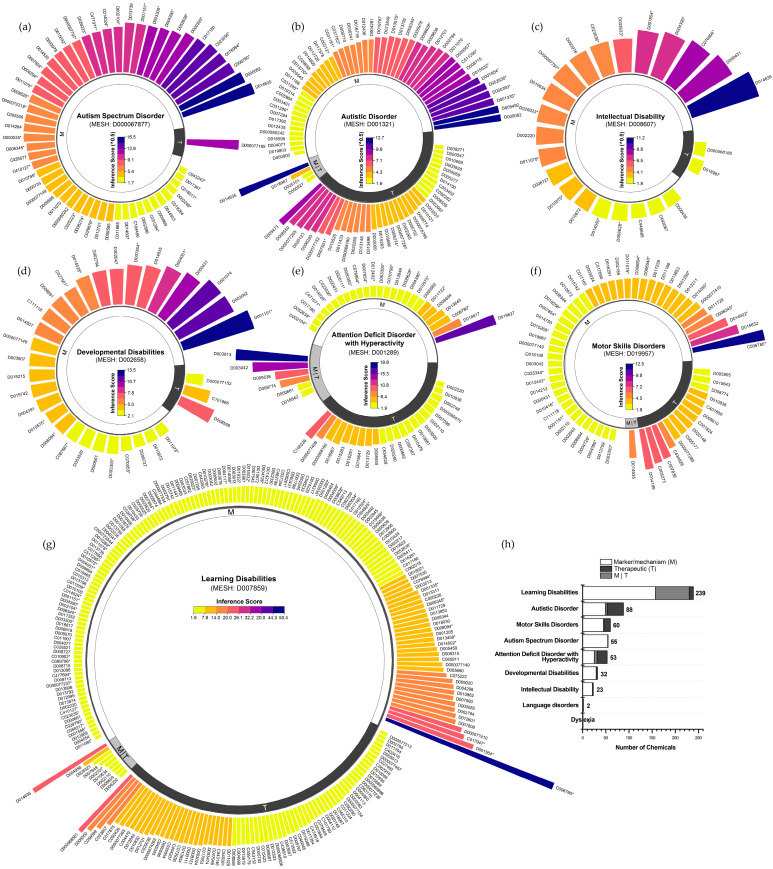
Frameworks for chemical exposome in neurodevelopmental disorders from the Comparative Toxicogenomics Database (CTD). (**a**) Chemicals with curated marker/mechanism and therapeutic associations with autism spectrum disorder, ranked by inference scores. (**b**–**g**) Curated chemicals associated with autism disorder, intellectual disability, developmental disabilities, attention deficit hyperactivity disorder, motor skills disorders, and learning disabilities. (**h**) Proportion of curated marker/mechanism and therapeutic chemicals in neurodevelopmental disorders. **Notes:** Environmental pollutants were denoted with an asterisk (*). To ensure clarity and conciseness, chemical IDs were used in place of full chemical names, which are detailed in Supplementary Methods S1.2.

Based on the CTD, we systematically identified and characterized the chemical exposome associated with NDDs, ranking chemicals by curated marker/mechanism and therapeutic associations using chemical–disease inference scores. ASD and autistic disorder, both descendants of the “Child Development Disorders, Pervasive” hierarchical category, included 55 and 88 chemicals, respectively. ASD chemicals were predominantly linked to markers/mechanisms (inference scores: 2.8–270.1; median 47.2; Figure 1a), whereas autistic disorder encompassed both markers/mechanisms and therapeutic associations (inference scores: 2.5–159.4; median 16.4; Figure 1b). ID and developmental disabilities were associated with fewer chemicals, 23 (inference scores: 3.1–123.6; median 18.7; Figure 1c) and 32 (inference scores: 2.1–13.5; median 4.8; Figure 1d), respectively, with most linked to markers/mechanisms. Similarly, attention deficit disorder with hyperactivity involved 53 chemicals (inference scores: 2.0–19.7; median 3.7; Figure 1e), and motor skills disorders included 60 chemicals (inference scores: 2.0–12.4; median 3.2; Figure 1f). Additionally, learning disabilities were associated with the largest number of chemicals (n = 239), spanning both markers/mechanisms and therapeutic associations (inference scores: 1.9–50.4; median 3.2; Figure 1g). These results reveal distinct variations in chemical exposomes across NDDs, highlighting differences in chemical involvement and the strength of chemical–disease interactions (Figure 1h).

**Figure 2:**
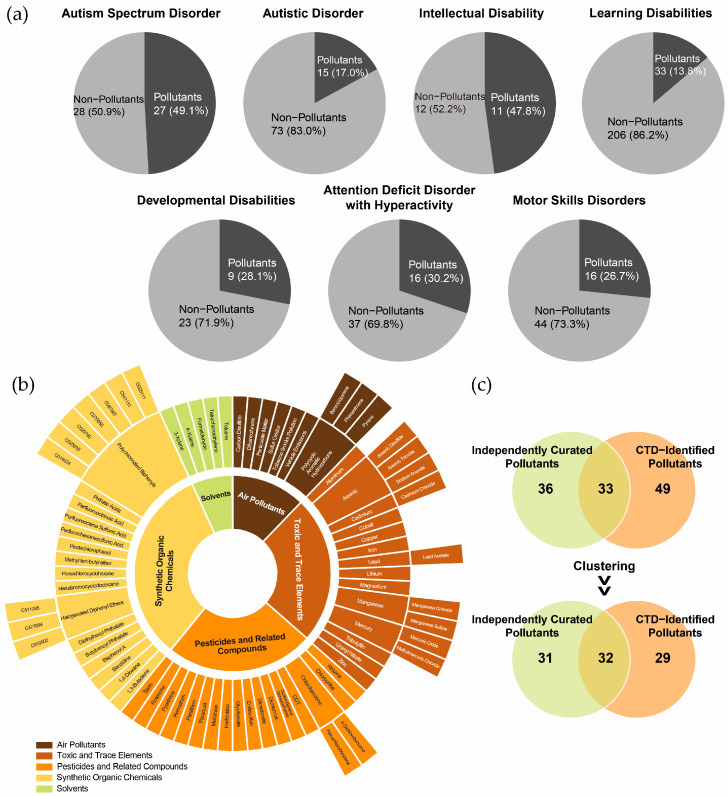
Identification of environmental pollutants in the chemical exposome of neurodevelopmental disorders. (**a**) Proportion of environmental pollutants among chemicals associated with neurodevelopmental disorders. (**b**) Classification of environmental pollutants identified in the Comparative Toxicogenomics Database (CTD). (**c**) Overlap between CTD-identified environmental pollutants and independently curated environmental pollutants, with clustering based on broader substance categories.

Notably, chemical pollutants accounted for 17.0% to 47.8% of the chemicals associated with these NDDs (Figure 2a). Among the chemicals with the highest marker/mechanism inference scores or each disorder, the following pollutants were identified: for ASD and autistic disorder, valproic acid, acetaminophen, air pollutants, and some metal elements; for ID, PFOS, chlorpyrifos, and Pb; for developmental disabilities, As and DEHP; for attention deficit disorder with hyperactivity, BPA; for motor skills disorders, BPA, uranyl nitrate, and Mn; and for learning disabilities, BPA, benzo(a)pyrene, and sodium arsenite. These findings underscore the critical role of chemical pollutants in shaping the chemical exposomes associated with NDDs.

The CTD-identified chemical pollutants were further categorized into air pollutants (n = 8), toxic and trace elements (n = 14), pesticides and related compounds (n = 18), synthetic organic chemicals (n = 16), and solvents (n = 5) (Figure 2b). A comparison of CTD-identified pollutants with independently curated datasets revealed an overlap exceeding 50%, indicating reasonable consistency in pollutant classification (Figure 2c). However, the non-overlapping pollutants suggest that the independently curated list of complementary pollutants could further enhance the comprehensiveness of the CTD-based chemical exposomes.

### 3.2. Enrichment of Chemical-Pollutant-Associated Genes in Neurodevelopmental Disorders

**Figure 3:**
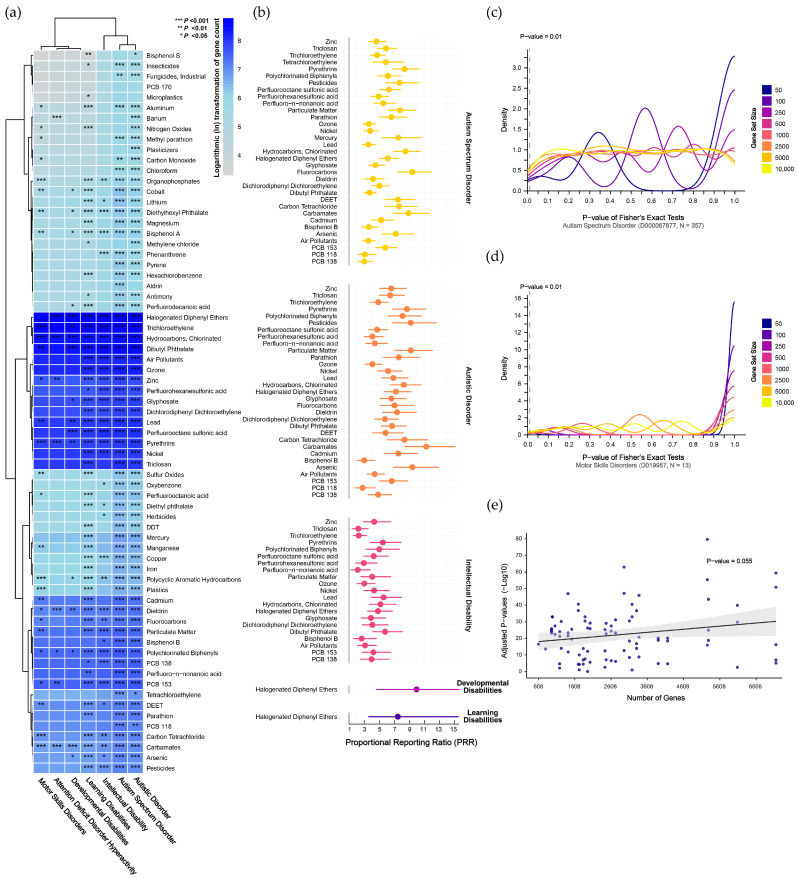
Gene set enrichment analysis revealing genetic associations between chemical pollutants and neurodevelopmental disorders. (**a**) Preliminary analyses of chemical pollutants identified through evidence-based and public-health-oriented approaches. (**b**) Refined analyses excluding contingency tables with any cell having an expected frequency ≤1, indicating the magnitude of enrichment using the proportional reporting ratio. (**c**,**d**) Density plots showing the results of cross-validation testing for autism spectrum disorder, which has the largest gene set, and motor skills disorders, which have the smallest gene set. (**e**) Linear regression indicating no significant relationship between the number of genes and significant enrichments. **Abbreviations:** DDT, dichloro-diphenyl-trichloroethane; DEET, N,N-diethyl-3-methylbenzamide; PCB 118, 2,3′,4,4′,5-pentachlorobiphenyl; PCB 138, 2,2′,3′,4,4′,5-hexachlorobiphenyl; PCB 153, 2,4,5,2′,4′,5′-hexachlorobiphenyl; PCBs, polychlorinated biphenyls; PFHxS, perfluorohexanesulfonic acid; PFNA, perfluoro-n-nonanoic acid; PFOS, perfluorooctane sulfonic acid; PM, particulate matter.

Mapping disorder- and pollutant-associated gene sets to the human genome revealed consistently high mapping rates for disorder gene sets (ranging from 90.9% to 100%, with a median of 99.4%), whereas pollutant-associated gene sets exhibited relatively lower mapping rates (ranging from 64.3% to 100%, with a median of 93.0%). Using the mapped genes, we conducted preliminary GSEA for 69 chemical pollutants across seven NDDs (Supplementary Results Section S2.1). As shown in Figure 3a, widespread pollutant enrichments were observed across disorders (corrected *p* < 0.05). Notably, ASD and autistic disorder, which are distinct diagnostic versions within the same overarching category, exhibited the most similar enrichment patterns. Other NDDs, including learning disabilities and ID, also showed significant pollutant enrichments. In contrast, attention deficit disorder with hyperactivity, developmental disabilities, and motor skills disorders exhibited fewer significant enrichments.

To refine our analysis, we excluded contingency tables with any cells having an expected frequency ≤1 and used Fisher’s exact test to confirm the significant enrichments identified in the preliminary analysis. This approach led to the identification of 84 pollutants that were significantly enriched across the disorders (corrected *p* < 0.05). Specifically, 33 pollutants were significantly enriched in ASD, 31 in autistic disorder, 21 in ID, and 1 each in developmental disabilities and learning disabilities (Supplementary Results Section S2.2). To further quantify the degree of pollutant enrichment within each disorder, we calculated the proportional reporting ratio (PRR), as shown in Figure 3b and Supplementary Results Section S2.2. For ASD, the pollutant enrichment strength ranged from 3.05 (95% CI: 2.23–4.18) for PCB-138 to 9.41 (95% CI: 7.43–11.91) for fluorocarbons, with a median PRR of 5.81. For autistic disorder, the PRR ranged from 2.8 (95% CI: 1.76–4.45) for PCB-118 to 11.27 (95% CI: 8.41–15.12) for carbamates, with a median of 6.68. In ID, the PRR ranged from 2.17 (95% CI: 1.23–3.83) for PFNA to 5.79 (95% CI: 4.15–8.09) for DBP, with a median of 4.1. Both developmental disabilities and learning disabilities were uniquely associated with HDPE, with PRRs of 9.98 (95% CI: 4.67–21.31) and 7.48 (95% CI: 3.61–15.53), respectively.

We generated pseudo-pollutant gene sets through permutation to validate the enrichment results in NDDs. For ASD, with the largest number of disorder-associated genes, results were unstable with smaller pseudo-gene sets but became more stable as set size increased. At a false-positive rate threshold of 10 per 1000 tests, the 250- and 2500-gene sets reached 10 positive hits, but these false-positive rates did not survive Bonferroni correction (Figure 3c). For motor skills disorders, with the fewest disorder-associated genes, results were stable across different pseudo-gene sets, with positive hits ranging from 0 to 8, all below the false-positive threshold (Figure 3d). Validation results for other disorders are presented in Supplementary Results Section S2.3, where none exceeded the adjusted false-positive threshold, indicating stable enrichment findings. Additionally, no trend of increasing enrichment significance with larger gene sets was observed (*p* = 0.055, Figure 3e).

**Figure 4:**
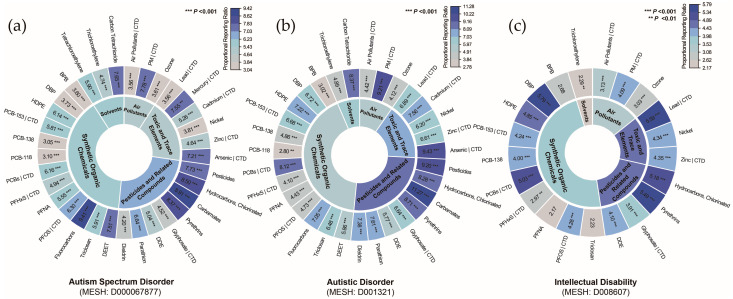
Classification of chemical pollutants identified by gene set enrichment analysis for (**a**) autism spectrum disorder, (**b**) autistic disorder, and (**c**) intellectual disability. **Notes:** Overlap with curated associations from the Comparative Toxicogenomics Database (CTD) is indicated by a vertical bar (|). **Abbreviations:** BPB, bisphenol B; DBP, dibutyl phthalate; DDE, dichlorodiphenyl dichloroethylene; DEET, N,N-diethyl-3-methylbenzamide; HDPE, halogenated diphenyl ethers; PCB-118, 2,3′,4,4′,5-pentachlorobiphenyl; PCB-138, 2,2′,3′,4,4′,5-hexachlorobiphenyl; PCB-153, 2,4,5,2′,4′,5′-hexachlorobiphenyl; PCBs, polychlorinated biphenyls; PFHxS, perfluorohexanesulfonic acid; PFNA, perfluoro-n-nonanoic acid; PFOS, perfluorooctane sulfonic acid; PM, particulate matter.

The significantly enriched pollutants were compared with those associated with NDDs in the CTD, revealing 12 pollutants linked to ASD (Figure 4a), 11 pollutants linked to autistic disorder (Figure 4b), and 9 pollutants linked to ID (Figure 4c). The prioritized list of pollutants encompasses four major categories: air pollutants, toxic and trace elements, pesticides and related compounds, and synthetic organic chemicals, indicating a broad association between NDDs and chemical pollutants. Since the pollutants identified for ASD and autistic disorder were highly consistent, and autistic disorder represents an outdated diagnostic category, it was excluded from subsequent analyses. The pollutant–disorder gene sets for ASD and ID were subsequently extracted for further investigation.

### 3.3. Biological Functions of Shared Genes in Chemical Pollutants and Neurodevelopmental Disorders

**Figure 5:**
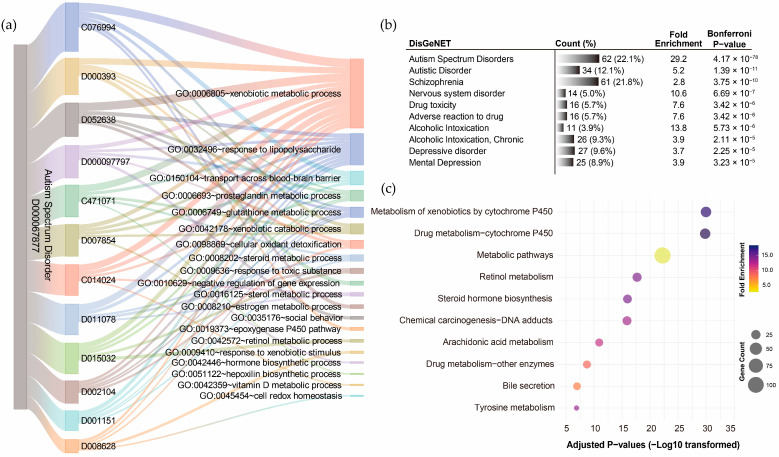
Functional annotation of shared genes among chemical pollutants associated with autism spectrum disorder. (**a**) Gene Ontology (GO) term annotation highlighting the overlaps of the top five enriched biological processes for each pollutant. (**b**) The top ten disease associations of the integrated chemical pollutant gene set. (**c**) KEGG pathway annotation of the integrated chemical pollutant gene set.

GO term annotation of the 12 pollutant–disorder gene sets associated with ASD revealed that the shared genes were enriched in diverse biological processes (Supplementary Results Section S2.4). Using a Sankey diagram, the overlaps among the top five enriched biological processes identified for each pollutant were visualized (Figure 5a). The *xenobiotic metabolic process* emerged as the most frequently overlapping biological process, shared by all 12 pollutants, with corrected *p*-values ranging from 1.63 × 10^−23^ to 1.18 × 10^−4^. The *response to lipopolysaccharide* was the second most frequent (10/12 pollutants), followed by *prostaglandin metabolic process* (5/12), *glutathione metabolic process* (4/12), *xenobiotic catabolic process* (4/12), and *transport across the blood–brain barrier* (4/12). Exploration of disease associations for the integrated ASD pollutant gene sets using DisGeNET further confirmed that this gene set effectively represents ASD-related genetic characteristics, with the highest correlation observed for ASD (corrected *p*-value = 4.17 × 10^−78^). Strong associations were also identified with autistic disorder (corrected *p*-value = 1.39 × 10^−11^) and schizophrenia (corrected *p*-value = 3.75 × 10^−10^) and other neuropsychiatric disorders (Figure 5b). KEGG pathway annotation of the integrated ASD pollutant gene sets revealed significant enrichment in metabolism-related pathways, particularly the *metabolism of xenobiotics by cytochrome P450* (corrected *p*-value = 1.06 × 10^−30^), followed by *drug metabolism–cytochrome P450* (corrected *p*-value = 1.52 × 10^−30^) and *metabolic pathways* (corrected *p*-value = 5.96 × 10^−23^) (Figure 5c). These findings support the significance of the xenobiotic metabolic process/pathway as a critical mechanism through which pollutants may influence the development of ASD.

**Figure 6:**
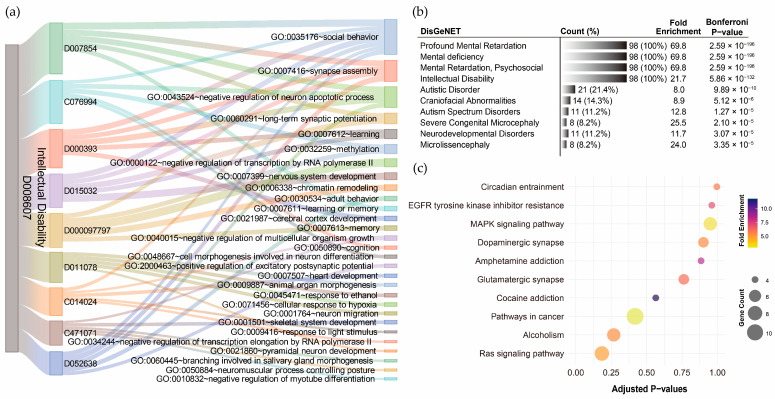
Functional annotation of shared genes among chemical pollutants associated with intellectual disability. (**a**) Gene Ontology (GO) term annotation highlighting the overlaps of the top five enriched biological processes for each pollutant. (**b**) The top ten disease associations of the integrated chemical pollutant gene set. (**c**) KEGG pathway annotation of the integrated chemical pollutant gene set.

For ID, GO term annotation of the nine pollutant–disorder gene sets revealed relatively few biological processes (Supplementary Results Section S2.5). The Sankey diagram showed that *social behavior* was the most frequently overlapping biological process, with seven out of nine pollutants indicating involvement in this pathway (Figure 6a). However, only five out of nine pollutants exhibited statistical significance after Bonferroni correction, with corrected *p*-values ranging from 3.55 × 10^−2^ to 7.61 × 10^−5^. *Synapse assembly* was the second most frequently overlapping process (4/9), with significant enrichment observed for particulate matter (*p*-value = 2.96 × 10^−4^) and lead (*p*-value = 8.50 × 10^−3^). The *negative regulation of neuron apoptotic process* also relatively overlapped (4/9), but none of the pollutants showed statistical significance after Bonferroni correction. DisGeNET analysis revealed that the integrated ID pollutant gene sets were highly associated with profound mental retardation, mental deficiency, mental retardation (psychosocial), and ID, with corrected *p*-values ranging from 2.59 × 10^−196^ to 5.68 × 10^−132^ (Figure 6b). However, no significant KEGG pathways were annotated to the integrated ID pollutant gene sets (Figure 6c). This suggests that the current genetic associations related to ID and pollutants might be limited.

### 3.4. Temporal-Specific Expression Patterns of Pollutant–Disorder Gene Sets

**Figure 7:**
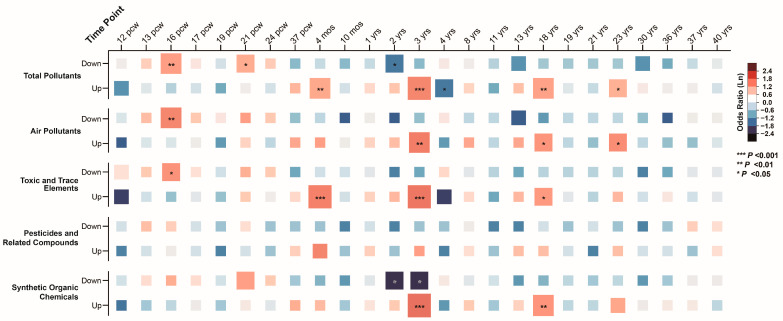
Associations between integrated autism spectrum disorder pollutant genes and high/low-expression genes across brain developmental stages.

In the BrainSpan database, we analyzed gene expression across 25 time points, spanning from 12 postconceptual weeks (pcw) to 40 years (yrs), identifying gene groups with significantly higher or lower expression levels. Association analysis between ASD pollutant gene sets and these high/low expression groups revealed that ASD pollutant gene sets were enriched in low-expression genes during prenatal stages (16 pcw and 21 pcw; corrected *p*-values = 0.002 and 0.027). In contrast, these gene sets were enriched in high-expression genes during postnatal stages (4 months, 3 years, 18 years, and 23 years), with corrected *p*-values ranging from 3.17 × 10^−6^ to 0.046 (Figure 7). Similar associations were observed across air pollutants, toxic and trace elements, and synthetic organic chemicals, highlighting the temporal-specific expression patterns of ASD pollutant gene sets (Supplementary Results Sections S2.6.1–S6.2).

**Figure 8:**
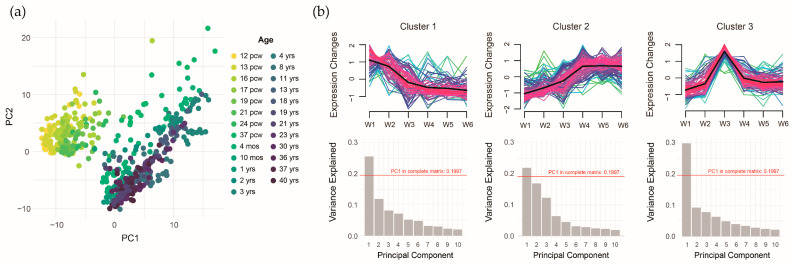
Temporal-specific expression patterns of integrated autism spectrum disorder pollutant genes. (**a**) Distribution of the first/second principal components across developmental time points. (**b**) Three distinct gene expression trajectories with the variance explained by principal components.

To further investigate these associations, we performed principal component analysis (PCA) to extract the expression features of the ASD pollutant gene sets. The scatter plot of the first two principal components (PC1 and PC2) clearly separated prenatal and postnatal samples (Figure 8a). However, the overall explanatory power was low, with the first ten components explaining only 60% of the variance. To address this, we applied trajectory analysis to identify gene clusters with distinct expression patterns, reducing potential heterogeneity in gene expression. The time trajectory model identified three gene expression patterns: 76 genes exhibiting higher expression prenatally (Cluster 1), 113 genes exhibiting higher expression postnatally (Cluster 2), and 86 genes exhibiting higher expression around the perinatal period (Cluster 3). Clustering improved the explanatory power of PC1, suggesting that grouping reduced gene expression heterogeneity within the ASD pollutant gene sets (Figure 8b).

**Figure 9:**
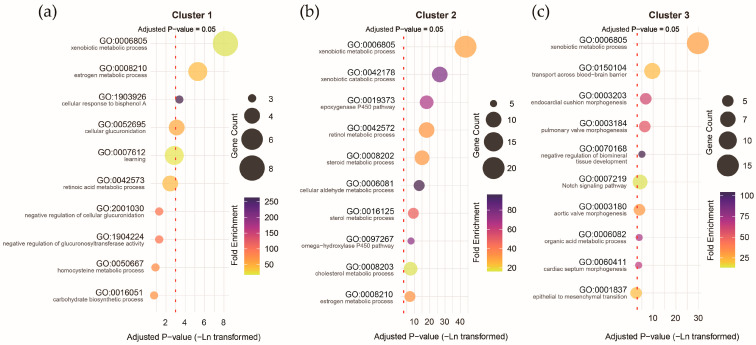
Gene Ontology (GO) term biological process annotation for the genes with different expression trajectories in Cluster 1 (**a**), Cluster 2 (**b**), and Cluster 3 (**c**).

Functional annotation of the clusters revealed that all three were strongly associated with the *xenobiotic metabolic process* (corrected *p*-values: 2.89 × 10^−4^ to 1.37 × 10^−19^), but other biological processes exhibited considerable differences. Genes with higher expression prenatally were more associated with the *estrogen metabolic process* (corrected *p*-value = 0.005; Figure 9a), genes with higher expression postnatally were more associated with the *xenobiotic catabolism* (corrected *p*-value = 2.82 × 10^−12^; Figure 9b), and genes with higher expression around the perinatal period were more associated with *transport across the blood–brain barrier* (corrected *p*-value = 6.87 × 10^−5^; Figure 9c). These results underscore the potential differential impact of pollutant exposure during distinct stages of brain development, with specific biological processes linked to prenatal, postnatal, and perinatal expression profiles of ASD pollutant genes.

Similar differential associations were observed between ID pollutant gene sets and BrainSpan gene expression groups, with transitions occurring during the early postnatal period (4 months–4 years) (Supplementary Results S2.6.3–S6.4). PCA of the ID pollutant gene expression matrix also clearly separated prenatal and postnatal samples. Time trajectory analysis of ID pollutant genes revealed three gene expression patterns, akin to those observed in ASD: 19 genes with higher expression prenatally (Cluster 1), 30 genes with higher expression postnatally (Cluster 2), and 44 genes with higher expression around the perinatal period (Cluster 3). However, functional annotation of the clusters revealed no overlap in biological processes. Genes in Cluster 1 were significantly associated with *membrane depolarization during action potential* (corrected *p*-value = 0.040), Cluster 2 genes were associated with *cognition* (corrected *p*-value = 0.030), and Cluster 3 genes were associated with *positive regulation of DNA-templated transcription* (corrected *p*-value = 0.009). The transcriptome signatures of the ID pollutant gene set further underscore the relationship between brain developmental stages and differential biological processes.

## 4. Discussion

According to the Global Burden of Disease Study 2021, the prevalence of NDDs has significantly increased over the past two decades (since 1990): ASD by 47.5% (95% CI: 45.1–49.4), idiopathic ID by 10.2% (95% CI: 5.7–13.7), and ADHD by 18.8% (95% CI: 14.9–23.2) [31]. Simultaneously, evidence linking chemical exposure to NDDs has rapidly accumulated. In this study, we systematically investigated the mechanisms underlying the association between mixed chemical pollutant exposure and NDDs. Leveraging the CTD’s chemical exposome framework, we identified a broad range of chemical pollutants with curated associations to NDDs, encompassing major categories of pollutants. By cross-verifying and extending these associations with complementary datasets, we identified robust gene interactions related to both ASD and ID, particularly with air pollutants, toxic elements, pesticides, and synthetic organic chemicals. Functional annotation of pollutant–disorder gene sets revealed shared biological mechanisms potentially influenced by multiple pollutants, including metabolic processes for ASD and cognitive processes for ID. Further developmental transcriptomic analysis highlighted the critical role of the *xenobiotic metabolic process* in the association between pollutants and ASD, while also suggesting differences in underlying mechanisms across various developmental windows of exposure.

Research utilizing CTD data to investigate the chemical pollutant exposome of NDDs remains limited. A PubMed search combining CTD and NDDs revealed only a few relevant studies. For instance, Yang et al. identified 131 dyslexia susceptibility genes in the CTD and analyzed their overlap with 95 chemical compounds, including metals, persistent organic pollutants, PAHs, and pesticides, highlighting 35 pollutants that interact with dyslexia-associated genes [32]. In addition to NDDs, studies on other mental disorders have been conducted. Kafle et al. used CTD chemical–gene interaction data to identify five key chemicals—pinosylvin, bromobenzene, clonidine, gabapentin, and melatonin—that are directly associated with insomnia [33]. Furthermore, Živančević et al. explored the role of oxidative stress, particularly involving SOD2, in neurodegeneration induced by toxic metal mixtures (Pb, methylmercury, Cd, and As), offering new insights into shared molecular mechanisms underlying amyotrophic lateral sclerosis (ALS), Parkinson’s disease (PD), and Alzheimer’s disease (AD) [34]. It is increasingly evident that individuals are exposed to complex pollutant mixtures rather than isolated chemicals in real-world environments. While existing studies have elucidated the neurotoxic mechanisms of various pollutants, they typically focus on the potential effects of single exposures. This approach may neglect the synergistic and cumulative effects of multiple pollutants on neurodevelopment, highlighting a critical gap in current understanding. Systematic and integrative analyses of CTD data provide a valuable opportunity to address this gap, enabling a more comprehensive characterization of chemical pollutant interactions and their contributions to NDDs.

Exposure to a wide array of chemical pollutants and their mixtures poses risks of both acute and chronic toxicity [3]. Compared to adults, fetuses and children are particularly sensitive and vulnerable to these pollutants due to their rapidly developing brains and immature detoxification systems [1,2]. Our findings align with meta-analyses of epidemiological studies, which demonstrate extensive associations between chemical pollutant exposures and NDDs. Specifically, exposure to air pollutants (PM_1_, PM_2.5_, O_3_, NO_2_) [10,35,36,37], toxic elements (As, Co, Hg, Mg, Pb) [38,39,40,41], pesticides (organophosphates, pyrethroids) [42,43], synthetic organic pollutants (PCBs, PBDEs, PFASs, DEHPs, BPA) [44,45,46], and solvents [43] has been associated with various NDDs. Our cross-verification further confirmed that these major categories of chemical pollutants exhibit robust gene interactions associated with both ASD and ID. However, not all pollutants identified in meta-analytical associations were incorporated into the gene network. This discrepancy may suggest differences in the comprehensiveness of mechanistic research across chemical pollutants, potentially reflecting variations in their relative impact or weight within the overall gene network. Additionally, based on overlapping genes, we explored the association between chemical pollutants of public health significance, such as microplastics, and NDDs. However, GSEA did not reveal significant associations, indicating the need for further environmental epidemiological and mechanistic investigations to explore potential associations.

Through GO term annotation of chemical pollutant gene sets associated with ASD, we identified the *xenobiotic metabolic process* as a critical biological process shared among these pollutants. The primary function of xenobiotic metabolism is to eliminate physiologically unnecessary compounds, some of which may be harmful [47]. From an evolutionary perspective, this role underpins its designation as a detoxification process. This finding is therefore reasonable in the context of chemical pollutants, as most pollutants—particularly synthetic persistent organic pollutants—are unnecessary compounds for the human body and may even be harmful. In addition to xenobiotic metabolism, other metabolic biological processes were also widely associated with ASD. KEGG pathway annotation further highlighted metabolism-related pathways, such as cytochrome P450, underscoring the impact of pollutants on the metabolic burden of the body. However, these functions differ substantially from the pathogenic pathways suggested by genetic studies of ASD. Nevertheless, our analysis confirmed that these pollutant-related genes are highly associated with ASD, as indicated by the DisGeNET database (Bonferroni-corrected *p*-value = 4.17 × 10^−78^). Supporting this, human studies have identified SNPs in enzymes involved in xenobiotic metabolism, including paraoxonase 1 (PON1), glutathione-S-transferases (GSTM1 and GSTP1), δ-aminolevulinic acid dehydratase (ALAD), solute carrier family 40 member 1 (SLC40A1), and metal regulatory transcription factor 1 (MTF1), which have been associated with increased susceptibility to toxicants and ASD risk [48]. Therefore, the impact of pollutant mixtures on ASD may be mediated by disruptions in various metabolic processes in the body, rather than directly affecting core pathogenic pathways.

The functional annotation results for ID suggested that the impact of chemical pollutant mixtures may primarily target direct neurofunctional processes, including *cognition*, *social behavior*, *synapse assembly*, and *positive regulation of DNA-templated transcription*, among others. Although the overall GO term annotation of chemical pollutant gene sets associated with ID did not reveal highly overlapping shared biological processes, grouping genes based on their developmental expression patterns revealed that this may be due to significant differences in gene functions across distinct developmental windows. Interestingly, the major categories of pollutants associated with ID were similar to those for autism spectrum disorder (ASD), yet their pathways of action were markedly different. This could, to some extent, be explained from a mechanistic perspective, helping to clarify why environmental epidemiological studies report varying strengths of association between the same pollutants and ASD or ID.

The effects of chemical pollutant exposure can vary significantly over time and across different stages of neurodevelopment [11,49]. To explore this, we utilized the BrainSpan database to investigate the developmental transcriptome signatures of the pollutant–disorder gene sets identified by GSEA. Previous studies have suggested that developmental age is the primary source of variation in gene expression across BrainSpan samples [28,50]. We also observed substantial differences in the expression of pollutant–disorder gene sets between prenatal and postnatal periods, with these genes enriched in low-expression categories prenatally and shifting to high-expression categories postnatally. During pregnancy, fetal exposure to pollutants occurs primarily through the placental pathway via maternal circulation, whereas postnatal exposure increases significantly as infants directly encounter pollutants through air, food, and their physical surroundings [51]. Although the BrainSpan database is derived from clinically unremarkable donors, these individuals were likely exposed to various chemicals. Furthermore, previous analyses of BrainSpan data have identified three distinct gene expression trajectories: “rising” genes, “falling” genes, and “non-transitional” genes [52]. Similarly, our study identified three expression patterns among pollutant-related genes: those with higher expression prenatally, those with higher expression postnatally, and those with elevated expression around the perinatal period. Functional annotation of these distinct expression trajectories revealed that genes following different trajectories may participate in distinct biological processes relevant to NDDs. By emphasizing the developmental perspective of pollutant–gene interactions, our findings provide novel insights into the potential mechanisms underlying the relationship between chemical pollutants and NDDs.

Several limitations of this study warrant attention. The enrichment results demonstrate statistical associations between genes impacted by pollutants and those implicated in NDDs. The curated chemical–disease associations in the CTD are derived from published literature curated by CTD biocurators. As such, the enrichment analysis used to cross-verify and extend CTD-curated associations primarily relies on statistical testing. A lack of statistically significant pollutants or pathways does not necessarily indicate the absence of potential biological relevance. Furthermore, this analysis did not account for dose variations in specific pollutant exposures, leading to the formulation of several hypothetical mechanisms. Real-world exposure to pollutant mixtures likely involves more complex interactions, underscoring the need for future studies to validate these findings under diverse environmental conditions. Another limitation is the lack of tissue specificity, as the pollutant-associated genes were derived from studies involving various tissues and species. Nonetheless, mapping these genes to the human genome constrained the analytical background, and the use of BrainSpan transcriptomic data enabled the characterization of their expression patterns in the human brain, which may partially mitigate this limitation. Lastly, as the CTD toxicogenomic data are updated monthly, this study reflects the most current evidence. With ongoing research, the pollutant exposome linked to NDDs will likely become more robust and comprehensive. Nonetheless, this study offers a reproducible framework for exploring broader associations between chemical pollutants and neuropsychiatric disorders.

## 5. Conclusions

Taken together, our results provide valuable insights into the genetic bases, biological mechanisms, and developmental transcriptome signatures associated with mixed chemical pollutant exposure in NDDs. By integrating publicly available databases, we demonstrate the potential of constructing a chemical exposome framework that systematically screens the molecular effects of chemical pollutants on disease-specific responses. This research model offers a viable approach for the systematic assessment of chemical pollutant risks from the perspective of disease molecular mechanisms, while also enhancing the understanding of new entities (chemical pollutants) and their impacts. Furthermore, by deepening our understanding of how chemical pollutants disrupt human health, it supports the transition to sustainable practices for the management of these pollutants.

## Data Availability

All research data are provided in the Supplementary Materials, with sources of public datasets cited in the relevant sections.

## Supplementary Materials

The following supporting information can be downloaded at: https://www.mdpi.com/article/10.3390/toxics13040282/s1, Supplementary Methods Sections S1.1–S1.7: Processing and analysis of CTD data; Supplementary Methods Section S1.8: Processing and analysis of BrainSpan data; Supplementary Results Section S2.1–S2.2: Complete results of gene set enrichment analysis; Supplementary Results Section S2.3: Cross-validation of enrichment results; Supplementary Results Section S2.4–S2.5: Functional annotation; Supplementary Results Section S2.6: Complete results of temporal-specific expression analysis.
