# Supplementary material for: Chemical Pollutant Exposure in Neurodevelopmental Disorders: Integrating Toxicogenomic and Transcriptomic Evidence to Elucidate Shared Biological Mechanisms and Developmental Signatures
Source: Toxics. 2025 Apr 8;13(4):282. doi: 10.3390/toxics13040282 (PMC12031255; doi:10.3390/toxics13040282)
Supplement: Supplementary file 1 [file toxics-13-00282-s001.zip › toxics-3511376-supplementary.pdf]

Supplemental Information for

Chemical Pollutant Exposure in Neurodevelopmental Disorders: Integrating Toxicogenomic and Transcriptomic Evidence to Elucidate Shared Biological Mechanisms and Developmental Signatures

Xuping Gao et, al.

The supplementary materials encompass details on the processing and analysis of CTD data (Sections S1.1–S1.7) and BrainSpan data (Section S1.8), as well as in-depth results from gene set enrichment analysis (Sections S2.1–S2.2), cross-validation of enrichment (Section S2.3), functional annotation (Sections S2.4–S2.5), and temporal-specific expression analysis (Section S2.6).

Figures S1 to S20

Tables S1 to S60

Supplemental References

Content

S1. Supplementary Methods 1

S1.1 Identification of Neurodevelopmental Disorders in Comparative Toxicogenomics Database 1

S1.2 Chemical Exposome of Neurodevelopmental Disorders 4

S1.2.1 Branches of Attention Deficit and Disruptive Behavior Disorders 4

S1.2.2 Branches of Child Development Disorders, Pervasive 6

S1.2.3 Branches of Communication Disorders 11

S1.2.4 Branches of Developmental Disabilities 12

S1.2.5 Branches of Intellectual Disability 13

S1.2.6 Branches of Learning Disabilities 14

S1.2.7 Branches of Motor Skills Disorders 22

S1.3 Chemical Pollutants Curated Independently of the Comparative Toxicogenomics Database 26

S1.4 Overlap Between CTD-Identified Pollutants and Independently Curated Pollutants 30

S1.5 Gene Sets for Neurodevelopmental Disorders and Independently Identified Environmental Pollutants 36

S1.5.1 Gene Sets of Neurodevelopmental Disorders 36

S1.5.2 Gene Sets of Environmental Pollutants 39

S1.6 Mapping Gene Sets onto the Homo sapiens Genome 43

S1.7 Identified Pollutant-Disorder Gene Sets 47

S1.8 Characterizing Developmental Transcriptome Signatures of Pollutant-Disorder Gene Sets 52

S1.8.1 BrainSpan Developmental Transcriptome Dataset 52

S1.8.2 Gene Expression Analysis 52

S1.8.3 Principal Component Analysis 52

S1.8.4 Trajectory Analysis 53

S2. Supplementary Results 54

S2.1 Testing Chemical Enrichment in Neurodevelopmental Disorders 54

S2.1.1 Enrichment Analysis for Attention Deficit Disorder with Hyperactivity (MESH: D001289) 54

S2.1.2 Enrichment Analysis for Autism Spectrum Disorder (MESH: D000067877) 58

S2.1.3 Enrichment Analysis for Autistic Disorder (MESH: D001321) 61
S2.1.4 Enrichment Analysis for Developmental Disabilities (MESH: D002658) 64
S2.1.5 Enrichment Analysis for Intellectual Disability (MESH: D008607) 67
S2.1.6 Enrichment Analysis for Learning Disabilities (MESH: D007859) 70
S2.1.7 Enrichment Analysis for Motor Skills Disorders (MESH: D019957) 73
S2.2 Enriched Chemicals Selection and Pattern Evaluation 76
S2.3 Cross-Validation of Enrichment Results.. 80
S2.4 Functional Annotation for Pollutant-Disorder Gene Sets of Autism Spectrum Disorder 84
S2.4.1 Significant GO Biological Processes for Pollutant-Disorder Gene Sets of Autism Spectrum Disorder 84
S2.4.2 Significant DisGeNET Associations for Pollutant-Disorder Gene Sets of Autism Spectrum Disorder 90
S2.4.3 Significant KEGG Pathways for Pollutant-Disorder Gene Sets of Autism Spectrum Disorder 92
S2.5 Functional Annotation for Pollutant-Disorder Gene Sets of Intellectual Disability 95
S2.5.1 Significant GO Biological Processes for Pollutant-Disorder Gene Sets of Intellectual Disability 95
S2.5.2 Significant DisGeNET Associations for Pollutant-Disorder Gene Sets of Intellectual Disability 98
S2.5.3 Significant KEGG Pathways for Pollutant-Disorder Gene Sets of Intellectual Disability100
S2.6 Temporal-Specific Expression Signatures 102
S2.6.1 Temporal Expression of Pollutant-Disorder Gene Sets for Autism Spectrum Disorder .. 102
S2.6.2 Temporal Trajectories of Pollutant-Disorder Gene Sets for Autism Spectrum Disorder. 106
S2.6.3 Temporal Expression of Pollutant-Disorder Gene Sets for Intellectual Disability 112
S2.6.4 Temporal Trajectories of Pollutant-Disorder Gene Sets for Intellectual Disability 117
Supplemental References 124

S1. Supplementary Methods

S1.1 Identification of Neurodevelopmental Disorders in Comparative Toxicogenomics Database

The Comparative Toxicogenomics Database (CTD) [1] utilizes MEDIC, which is structured as a polyhierarchical tree, allowing a term to serve as a node in multiple branches. We searched a total of 24 branches related to neurodevelopmental disorders and their descendants to collect toxicogenomic data on environmental exposures affecting neurodevelopment. The eligible data included information on associated chemicals, genes, phenotypes, and exposure references (date of download 10/30/2024).

Supplementary Table S1. Selection of Neurodevelopmental Disorders in CTD

MEDIC Categories and Hierarchical Descendants	MeSH® ID	OMIM® IDs	Data Status	Selected
● Anxiety, Separation	D001010	-		
● Attention Deficit and Disruptive Behavior Disorders	D019958	-		√
○ Attention Deficit Disorder with Hyperactivity	D001289	143465; 608903; 608904; 608905; 608906; 612311; 612312; 613003		√
○ Conduct Disorder	D019955	-		√
○ Oppositional Defiant Disorder	D000096865	-	-	
○ Sluggish Cognitive Tempo	D000087346	-	-	
● Bainbridge-Ropers syndrome	C000726367	-	-	
● Child Behavior Disorders	D002653	-		√
○ Abuse dwarfism syndrome	C535569	-	-	
● Child Development Disorders, Pervasive	D002659	-		√
○ Autism Spectrum Disorder	D000067877	-		√
◦ Asperger syndrome	D020817	608631; 608638; 608781; 609954		
◦ Autism, Susceptibility to, X-Linked 1	-	300425		
◦ Autism, Susceptibility to, X-Linked 2	-	300495		
◦ Autistic Disorder	D001321	209850; 300496; 300830; 300847; 300872; 606053; 607373; 608049; 608636; 609378; 610676; 610836; 610838; 610908; 611015; 611016; 611913; 612100;		√

		613410; 613436; 615032; 615091		
○ Helsmoortel-Van der Aa syndrome	C000730394	-	-	
**○** Hyperuricemia, Infantile, with Abnormal Behavior and Normal Hypoxanthine Guanine Phosphoribosyltransferase	C565489	-	-	
● Communication Disorders	D003147	-		√
**○** Childhood-Onset Fluency Disorder	D000067454	-	-	
**○** Language Disorders	D007806	-		√
○ Agraphia	D000381	-		
○ Anomia	D000849	-		
○ Dyslexia	D004410	127700; 300509; 600202; 604254; 606616; 606896; 608995		√
○ Giacheti syndrome	C567864	-	-	
○ Hyperlexia	C565500	-	-	
○ Language Development Disorders	D007805	-		
○ Neurodevelopmental Disorder with Hypotonia, Impaired Language, and Dysmorphic Features	-	616579		
○ Speech Disorders	D013064	-		
**○** Social Communication Disorder	D000067404	-		√
**○** Speech Sound Disorder	D066229	-	-	
● Developmental Disabilities	D002658	-		√
● Helsmoortel-Van der Aa syndrome	C000730394	-	-	
● Intellectual Disability	D008607	-		√
● Learning Disabilities	D007859	-		√
**○** Chromosome 10q26 Deletion syndrome	C567182	-	-	
**○** Chromosome 7q11.23 Deletion syndrome, Distal, 1.2-MB	-	613729	-	
**○** Epilepsy, X-Linked, with Variable Learning Disabilities and Behavior Disorders	C564505	300491		
**○** Giacheti syndrome	C567864	-	-	
**○** NF1 Microdeletion syndrome	C563524	-		
**○** non-verbal learning disabilities	C000726807	-	-	
**○** Riddle syndrome	C567453	611943		
**○** Slavotinek Pike Mills Hurst syndrome	C536672	-	-	

○ Specific Language Disorder	D000080888	-	-	
○ Specific Learning Disorder	D000067559	-		√
○ Agraphia	D000381	-		
○ Dyscalculia	D060705	-	-	
○ Dyslexia	D004410	127700; 300509; 600202; 604254; 606616; 606896; 608995		√
● Motor Skills Disorders	D019957	-		√
● Mutism	D009155	-		
● Neurodevelopmental Disorder with Dysmorphic Features, Spasticity, and Brain Abnormalities	-	615802		
● Neurodevelopmental Disorder with Epilepsy, Cataracts, Feeding Difficulties, and Delayed Brain Myelination	-	617393		
● Neurodevelopmental Disorder with Hypotonia, Seizures, and Absent Language	-	617268		
● Neurodevelopmental Disorder with or without Anomalies of the Brain, Eye, or Heart	-	616975		
● Neurodevelopmental Disorder with Progressive Microcephaly, Spasticity, and Brain Imaging Abnormalities	-	616486		
● Neurodevelopmental Disorder with Progressive Spasticity and Brain White Matter Abnormalities	-	619026		
● Neurodevelopmental Disorder with Spastic Paraplegia and Microcephaly	-	616281		
● Reactive Attachment Disorder	D019962	-		
● Savant syndrome	C000721847		-	
● Schizophrenia, Childhood	D012561	-		
● Stereotypic Movement Disorder	D019956	-		
● Tic Disorders	D013981	-		
○ Tourette Syndrome	D005879	137580		

Notes: indicates the data of associated chemicals, indicates the data of associated genes, indicates the data of associated exposure references, indicates the data of associated phenotypes

Abbreviations: MEDIC, CTD's 'merged disease vocabulary'; MeSH, National Library of Medicine's Medical Subject Headings; OMIM, the Online Mendelian Inheritance in Man.

S1.2 Chemical Exposome of Neurodevelopmental Disorders

We selected chemicals with curated associations to neurodevelopmental disorders (M *marker/mechanism and/or* T *therapeutic*) and inferred scores (date of download 10/31/2024). Eligible data were identified for attention deficit and disruptive behavior disorders (including attention deficit disorder with hyperactivity and conduct disorder), child behavior disorders, pervasive child development disorders (including autism spectrum disorder and autistic disorder), communication disorders (including language disorders, dyslexia, and social communication disorder), developmental disabilities, intellectual disability, learning disabilities (including specific learning disorder), and motor skills disorders. Due to the lack of inferred genes, conduct disorder, child behavior disorders, social communication disorder, specific learning disorder were subsequently excluded.

Inference scores measure the similarity between the CTD chemical–gene–disease networks and a comparable scale-free random network, with higher scores indicating atypical connectivity.[1] Many biological networks, including disease and metabolic networks, have been shown to be scale-free random networks. The inference score is calculated as the log-transformed product of two common-neighbor statistics that assess functional relationships in protein–protein interaction networks.[2] The first statistic evaluates the connectivity of the chemical and disease, along with the number of genes used for inference. The second statistic accounts for the connectivity of each gene involved.

S1.2.1 Branches of Attention Deficit and Disruptive Behavior Disorders

A total of 53 chemicals were identified for attention deficit disorder with hyperactivity, with 25 associated with markers/mechanisms, 22 associated with therapeutics, and six linked to both categories.

Supplementary Table S2. Chemical-Gene Interactions for Attention Deficit and Disruptive Behavior Disorders

Chemical Name	Chemical ID	Direct Evidence	Inference Score
**Attention Deficit Disorder with Hyperactivity (MESH: D001289)**			
Oxidopamine	D016627	M	17.13
N-Methyl-3,4-methylenedioxyamphetamine	D018817	M	8.23
bisphenol A	C006780	M	6.03
Tartrazine	D013645	M	5.62
Methamphetamine	D008694	M	4.47
Pyrethrins	D011722	M	4.26
Acetaminophen	D000082	M	3.78

Pesticides	D010575	M	3.74
Chlorpyrifos	D004390	M	3.65
Mercury	D008628	M	3.37
Thimerosal	D013849	M	3.11
Phthalic Acids	D010795	M	3.07
Copper	D003300	M	3.02
Rotenone	D012402	M	3.01
Lead	D007854	M	2.73
perfluorooctane sulfonic acid	C076994	M	2.73
Air Pollutants	D000393	M	2.61
Chlorodiphenyl (54% Chlorine)	D020111	M	2.55
Ethanol	D000431	M	2.52
perfluorooctanoic acid	C023036	M	2.31
Tobacco Smoke Pollution	D014028	M	2.24
perfluorohexanesulfonic acid	C471071	M	2.19
decamethrin	C017180	M	2.11
Particulate Matter	D052638	M	2.11
Cadmium	D002104	M	1.95
Dextroamphetamine	D003913	M | T	19.71
Cocaine	D003042	M | T	15.11
Nicotine	D009538	M | T	9.12
Methylphenidate	D008774	M | T	8.18
Amphetamine	D000661	M | T	3.79
Bupropion	D016642	M | T	3.49
pozanicline	C108326	T	8.79
Modafinil	D000077408	T	7.16
Aripiprazole	D000068180	T	6.22
Risperidone	D018967	T	6.22
Citalopram	D015283	T	5.03
Dizocilpine Maleate	D016291	T	5.03

2,3,4,5-Tetrahydro-7,8-dihydroxy-1-phenyl-1H-3-benzazepine	D015647	T	4.84
Terpenes	D013729	T	4.60
Melatonin	D008550	T	4.17
SCH 23390	C534628	T	4.09
Sertraline	D020280	T	3.80
Amphetamines	D000662	T	3.73
ginsenoside Rg3	C097367	T	3.70
Pergolide	D010479	T	3.59
Thioridazine	D013881	T	3.27
Clonidine	D003000	T	3.26
Caffeine	D002110	T	3.16
Imipramine	D007099	T	3.08
Venlafaxine Hydrochloride	D000069470	T	3.05
Chlorpromazine	D002746	T	3.01
Plant Extracts	D010936	T	2.37
Carbamazepine	D002220	T	2.16

S1.2.2 Branches of Child Development Disorders, Pervasive

A total of 55 chemicals were identified for autism spectrum disorder, with 54 associated with markers/mechanisms and one associated with therapeutics. For Autistic Disorder, 88 chemicals were identified, with 49 linked to markers/mechanisms, 35 to therapeutics, and four to both categories.

Supplementary Table S3. Chemical-Gene Interactions for Child Development Disorders, Pervasive

Chemical Name	Chemical ID	Direct Evidence	Inference Score
**Autism Spectrum Disorder (MESH: D000067877)**			
Valproic Acid	D014635	M	270.08
Acetaminophen	D000082	M	237.16
bisphenol A	C006780	M	205.93
perfluorooctane sulfonic acid	C076994	M	197.62

perfluorooctanoic acid	C023036	M	193.55
decamethrin	C017180	M	170.79
Air Pollutants	D000393	M	161.40
Particulate Matter	D052638	M	136.28
Chlorpyrifos	D004390	M	126.39
Copper	D003300	M	119.35
Arsenic	D001151	M	116.88
Testosterone	D013739	M	116.78
Cadmium	D002104	M	97.65
2,4,5,2',4',5'-hexachlorobiphenyl	C014024	M	91.56
perfluorohexanesulfonic acid	C471071	M	86.73
Permethrin	D026023	M	84.31
Glyphosate	D000097797	M	79.15
Zinc	D015032	M	78.60
Diazinon	D003976	M	75.41
Urethane	D014520	M	73.09
Lead	D007854	M	66.51
Malathion	D008294	M	56.91
Polychlorinated Biphenyls	D011078	M	55.86
Mercury	D008628	M	51.31
Dibenzofurans	D000072318	M	48.17
propionaldehyde	C005556	M	47.27
Triiodothyronine	D014284	M	47.15
Aluminum	D000535	M	46.10
Manganese	D008345	M	38.59
monobutyl phthalate	C028577	M	37.18
PCB 180	C410127	M	33.37
Phthalic Acids	D010795	M	28.51
Androstenedione	D000735	M	26.73
Sevoflurane	D000077149	M	26.54
Nitric Oxide	D009569	M	21.72

Poly I-C	D011070	M	19.01
8-Hydroxy-2'-Deoxyguanosine	D000080242	M	18.98
1,4-dioxane	C025223	M	18.83
Magnesium	D008274	M	16.79
benzidine	C029876	M	16.63
Serotonin	D012701	M	16.47
Nitrogen Dioxide	D009585	M	15.69
5-hydroxymethylcytosine	C011865	M	12.17
Vitamin D	D014807	M	11.52
25-hydroxyvitamin D	C104450	M	10.88
Cesium	D002586	M	8.86
chlorobenzene	C031294	M	8.67
4,4'-diphenylmethane diisocyanate	C005969	M	8.22
5-Methylcytosine	D044503	M	8.07
avermectin	C019264	M	7.90
Carbon Disulfide	D002246	M	6.15
4-dichlorobenzene	C018511	M	4.06
Selective Serotonin Reuptake Inhibitors	D017367	M	3.52
methyl tert-butyl ether	C043243	M	2.80
Resveratrol	D000077185	T	129.60
**Autistic Disorder (MESH: D001321)**			
Acetaminophen	D000082	M	131.00
Folic Acid	D005492	M	128.07
Vehicle Emissions	D001335	M	117.24
Air Pollutants	D000393	M	112.48
Particulate Matter	D052638	M	110.97
Lead	D007854	M	98.17
Zinc	D015032	M	78.84
Methionine	D008715	M	74.53
2,2',4,4'-tetrabromodiphenyl ether	C511295	M	73.38

Formaldehyde	D005557	M	72.61
Poly I-C	D011070	M	70.00
Choline	D002794	M	66.80
Serotonin	D012701	M	61.32
Norepinephrine	D009638	M	52.20
Mercury	D008628	M	50.97
Glutathione	D005978	M	49.35
Manganese	D008345	M	47.07
Thalidomide	D013792	M	42.89
Pesticides	D010575	M	42.25
Thimerosal	D013849	M	37.65
Linoleic Acid	D019787	M	36.44
Docosahexaenoic Acids	D004281	M	31.17
S-Adenosylmethionine	D012436	M	30.47
Arachidonic Acid	D016718	M	27.36
Adenosine	D000241	M	24.50
Homocysteine	D006710	M	24.22
1,3-butadiene	C031763	M	22.98
phenanthrene	C031181	M	21.28
phosphinothricin	C003121	M	16.41
Quinolinic Acid	D017378	M	15.36
Terbutaline	D013726	M	13.81
Vitamin B 12	D014805	M	11.96
propionic acid	C029658	M	11.94
Tetrachloroethylene	D013750	M	9.98
Cysteine	D003545	M	9.89
Porphyrins	D011166	M	9.38
3-xylene	C031285	M	7.42
Dehydroepiandrosterone Sulfate	D019314	M	7.37
5-methyltetrahydrofolate	C005984	M	7.14
Creatine	D003401	M	7.11

4-xylene	C031286	M	6.79
Inositol	D007294	M	6.23
Thiobarbituric Acid Reactive Substances	D017392	M	5.79
S-Adenosylhomocysteine	D012435	M	5.72
8-Hydroxy-2'-Deoxyguanosine	D000080242	M	5.58
Misoprostol	D016595	M	5.47
Digoxin	D004077	M	5.39
Glutathione Disulfide	D019803	M	5.22
Anti-Bacterial Agents	D000900	M	3.24
Valproic Acid	D014635	M | T	159.35
Risperidone	D018967	M | T	13.21
Vitamin B 6	D025101	M | T	3.51
Anticonvulsants	D000927	M | T	2.60
Fluoxetine	D005473	T	90.63
Melatonin	D008550	T	70.67
Pioglitazone	D000077205	T	64.17
Sirolimus	D020123	T	57.59
Haloperidol	D006220	T	51.78
Olanzapine	D000077152	T	46.88
Iron	D007501	T	43.38
Fatty Acids, Omega-3	D015525	T	27.96
Propranolol	D011433	T	25.94
Aripiprazole	D000068180	T	25.83
Sertraline	D020280	T	23.98
Spironolactone	D013148	T	21.90
Suramin	D013498	T	21.73
Clonidine	D003000	T	18.25
Citalopram	D015283	T	16.4
Fluvoxamine	D016666	T	15.36
Magnesium	D008274	T	15.23

Levetiracetam	D000077287	T	14.17
Buspirone	D002065	T	13.81
Galantamine	D005702	T	13.11
Mirtazapine	D000078785	T	12.49
Methylphenidate	D008774	T	10.61
Oxytocin	D010121	T	9.30
Cyproheptadine	D003533	T	9.16
Clomipramine	D002997	T	8.08
Memantine	D008559	T	7.55
ziprasidone	C092292	T	6.86
sapropterin	C003402	T	6.43
Antipsychotic Agents	D014150	T	4.71
Fenfluramine	D005277	T	3.94
Antihypertensive Agents	D000959	T	3.45
Antidepressive Agents	D000928	T	3.38
Piracetam	D010889	T	2.89
Amantadine	D000547	T	2.79
Naltrexone	D009271	T	2.45

S1.2.3 Branches of Communication Disorders

Two chemicals were identified for language disorders, both associated with markers/mechanisms, while dyslexia has one chemical associated with markers/mechanisms.

Supplementary Table S4. Chemical-Gene Interactions for Communication Disorders

Chemical Name	Chemical ID	Direct Evidence	Inference Score
**Language Disorders (MESH: D007806)**			
Nicotine	D009538	M	3.46
Carbamazepine	D002220	M	3.23
**Dyslexia (MESH: D004410)**			
Manganese	D008345	M	4.38

S1.2.4 Branches of Developmental Disabilities

A total of 32 chemicals were identified for developmental disabilities, with 29 associated with markers/mechanisms and three associated with therapeutics.

Supplementary Table S5. Chemical-Gene Interactions for Developmental Disabilities

Chemical Name	Chemical ID	Direct Evidence	Inference Score
**Developmental Disabilities (MESH: D002658)**			
Arsenic	D001151	M	13.50
Cocaine	D003042	M	11.24
Propoxur	D001074	M	11.10
Ethanol	D000431	M	9.58
Diethylhexyl Phthalate	D004051	M	8.77
Valproic Acid	D014635	M	7.71
Lead	D007854	M	7.27
Buprenorphine	D002047	M	6.46
Choline	D002794	M	6.43
Tobacco Smoke Pollution	D014028	M	6.28
butylbenzyl phthalate	C027561	M	5.28
Methadone	D008691	M	5.28
2',3,3',4',5-pentachloro-4-hydroxybiphenyl	C111118	M	5.18
Vitamin D	D014807	M	5.08
Sevoflurane	D000077149	M	4.85
Dexamethasone	D003907	M	4.66
Zidovudine	D015215	M	4.62
Propofol	D015742	M	4.61
Dust	D004391	M	4.33
Pesticides	D010575	M	3.82
Lithium	D008094	M	3.66

2,3,3',4,4',5-hexachlorobiphenyl	C087667	M	3.48
Cyclophosphamide	D003520	M	3.29
Amphetamine	D000661	M	3.23
Copper	D003300	M	3.23
2,3',4,4',5-pentachlorobiphenyl	C070055	M	2.64
Methotrexate	D008727	M	2.26
Phenytoin	D010672	M	2.13
Polychlorinated Biphenyls	D011078	M	2.11
Memantine	D008559	T	6.51
ezogabine	C101866	T	4.78
Olanzapine	D000077152	T	3.14

S1.2.5 Branches of Intellectual Disability

A total of 23 chemicals were identified for intellectual disability, with 21 associated with markers/mechanisms and two associated with therapeutics.

Supplementary Table S6. Chemical-Gene Interactions for Intellectual Disability

Chemical Name	Chemical ID	Direct Evidence	Inference Score
**Intellectual Disability (MESH: D008607)**			
Valproic Acid	D014635	M	123.55
Ethanol	D000431	M	76.85
perfluorooctane sulfonic acid	C076994	M	63.36
Chlorpyrifos	D004390	M	53.00
Lead	D007854	M	44.62
Dichlorodiphenyl Dichloroethylene	D003633	M	28.18
perfluorooctanoic acid	C023036	M	27.39
Diazinon	D003976	M	25.65
Glyphosate	D000097797	M	25.02
Phenobarbital	D010634	M	21.94

Permethrin	D026023	M	19.96
Carbamazepine	D002220	M	18.73
Polychlorinated Biphenyls	D011078	M	16.90
Methotrexate	D008727	M	13.89
Pesticides	D010575	M	10.45
Phenytoin	D010672	M	8.63
Toluene	D014050	M	8.45
Mercury	D008628	M	7.33
systhane	C446685	M	5.09
diphenylarsinic acid	C493087	M	5.04
Alcohols	D000438	M	4.74
Risperidone	D018967	T	3.74
Aripiprazole	D000068180	T	3.13

S1.2.6 Branches of Learning Disabilities

A total of 239 chemicals were identified for learning disabilities, with 157 associated with markers/mechanisms, 73 associated with therapeutics, and nine linked to both categories.

Supplementary Table S7. Chemical-Gene Interactions for Learning Disabilities

Chemical Name	Chemical ID	Direct Evidence	Inference Score
**Learning Disabilities (MESH: D007859)**			
bisphenol A	C006780	M	50.38
Benzo(a)pyrene	D001564	M	25.62
sodium arsenite	C017947	M	22.25
Aluminum Chloride	D000077410	M	20.99
Kainic Acid	D007608	M	18.75
Scopolamine	D012601	M	18.36
Cholesterol	D002784	M	17.69

Acetaminophen	D000082	M	17.44
Levodopa	D007980	M	16.63
Pilocarpine	D010862	M	16.63
Dopamine	D004298	M	16.25
Morphine	D009020	M	15.81
amyloid beta-protein (1-42)	C075222	M	13.99
Galactose	D005690	M	13.07
Sevoflurane	D000077149	M	12.66
2-(4-morpholinyl)-8-phenyl-4H-1-benzopyran-4-one	C085911	M	11.74
Malondialdehyde	D008315	M	11.70
Fluorides	D005459	M	11.23
Uranyl Nitrate	D014502	M	10.69
Sulfur Dioxide	D013458	M	10.59
Ascorbic Acid	D001205	M	10.56
Lithium	D008094	M	10.29
Silver Compounds	D018030	M	10.14
Maneb	D008344	M	10.01
Thioacetamide	D013853	M	9.95
Pyridostigmine Bromide	D011729	M	9.82
Manganese	D008345	M	9.71
aristolochic acid I	C000228	M	9.60
Streptozocin	D013311	M	9.52
Vehicle Emissions	D001335	M	9.46
Cycloheximide	D003513	M	8.28
perfluorooctane sulfonic acid	C076994	M	8.25
Isoflurane	D007530	M	8.08
Lithium Chloride	D018021	M	8.03
HU 211	C062018	M	7.84
decamethrin	C017180	M	7.83
Dizocilpine Maleate	D016291	M	7.76

Flame Retardants	D005411	M	7.59
Particulate Matter	D052638	M	7.52
Phencyclidine	D010622	M	7.40
Carbachol	D002217	M	7.38
Pentobarbital	D010424	M	7.38
lipoteichoic acid	C009900	M	7.36
Smoke	D012906	M	7.35
Amitriptyline	D000639	M	7.33
Nicotine	D009538	M	7.33
mercuric oxide	C019468	M	7.32
Cisplatin	D002945	M	7.26
Folic Acid	D005492	M	7.06
methylmercuric chloride	C004925	M	6.92
Sarin	D012524	M	6.90
cypermethrin	C017160	M	6.75
DDT	D003634	M	6.58
imidacloprid	C082359	M	6.52
isocarbophos	C549713	M	6.28
Mercury	D008628	M	6.12
Dichlorvos	D004006	M	6.07
Mecamylamine	D008464	M	6.06
Atrazine	D001280	M	5.99
Cobalt	D003035	M	5.57
Doxorubicin	D004317	M	5.55
Triazolam	D014229	M	5.33
mephedrone	C548233	M	5.2
Carbon Dioxide	D002245	M	5.16
Blood Glucose	D001786	M	5.08
Ethanol	D000431	M	4.88
lead acetate	C008261	M	4.87
cyhalothrin	C037304	M	4.79

lipopolysaccharide, E coli O55-B5	C482199	M	4.79
Carmustine	D002330	M	4.78
Tretinoin	D014212	M	4.73
Endosulfan	D004726	M	4.68
Phenytoin	D010672	M	4.67
Isoproterenol	D007545	M	4.65
Zinc	D015032	M	4.64
Dexamethasone	D003907	M	4.57
Copper Sulfate	D019327	M	4.42
Dextroamphetamine	D003913	M	4.33
Toluene	D014050	M	4.27
Dimethoate	D004117	M	4.26
Colchicine	D003078	M	4.24
Histamine	D006632	M	4.22
Cocaine	D003042	M	4.14
Parathion	D010278	M	4.11
gallium arsenide	C043055	M	3.95
Atropine	D001285	M	3.85
Permethrin	D026023	M	3.81
cyanoginosin LR	C057862	M	3.74
Cyproheptadine	D003533	M	3.71
Nitrogen Dioxide	D009585	M	3.70
Propylthiouracil	D011441	M	3.69
Propofol	D015742	M	3.68
Cholesterol, Dietary	D002791	M	3.66
Zolpidem	D000077334	M	3.66
ethylcholine aziridinium	C044894	M	3.65
Midazolam	D008874	M	3.65
Lead	D007854	M	3.54
Cyclophosphamide	D003520	M	3.51
Alprazolam	D000525	M	3.49

saikosaponin D	C025759	M	3.44
Pentylenetetrazole	D010433	M	3.40
manganese sulfate	C039798	M	3.38
Penicillins	D010406	M	3.37
Diazepam	D003975	M	3.30
Apomorphine	D001058	M	3.28
Dronabinol	D013759	M	3.26
Carbofuran	D002235	M	3.25
Dietary Fats	D004041	M	3.25
LPM4870108	C000723544	M	3.21
Paraquat	D010269	M	3.21
Polychlorinated Biphenyls	D011078	M	3.20
Testosterone	D013739	M	3.17
fenvalerate	C017690	M	2.97
2,2',4,4'-tetrabromodiphenyl ether	C511295	M	2.96
Pesticides	D010575	M	2.96
Diethylhexyl Phthalate	D004051	M	2.93
Methamphetamine	D008694	M	2.89
bioallethrin	C018465	M	2.80
pseudocumene	C010313	M	2.80
Dihydrotestosterone	D013196	M	2.77
tributyltin	C011559	M	2.77
Oxygen	D010100	M	2.74
2,4,5,2',4',5'-hexachlorobiphenyl	C014024	M	2.73
Arsenic	D001151	M	2.71
Chlorpyrifos	D004390	M	2.66
Cadmium	D002104	M	2.60
Herbicides	D006540	M	2.60
Procyclidine	D011352	M	2.55
Copper	D003300	M	2.53

N-Methyl-3,4-methylenedioxyamphetamine	D018817	M	2.52
Hydroxyurea	D006918	M	2.50
Lipopolysaccharides	D008070	M	2.43
melamine	C011907	M	2.43
Digoxin	D004077	M	2.41
lanthanum chloride	C028521	M	2.37
Methotrexate	D008727	M	2.35
decabromobiphenyl ether	C010902	M	2.34
hexabromocyclododecane	C089796	M	2.34
Methionine	D008715	M	2.34
Spermine	D013096	M	2.33
2,2',4,4',5-brominated diphenyl ether	C477694	M	2.31
Methimazole	D008713	M	2.31
Arsenic Trioxide	D000077237	M	2.30
Theophylline	D013806	M	2.30
Thalidomide	D013792	M	2.27
Soman	D012999	M	2.19
Thiopental	D013874	M	2.17
Carbamazepine	D002220	M	2.16
PCB 180	C410127	M	2.14
3,4,5,3',4'-pentachlorobiphenyl	C023035	M	2.13
Amphetamine	D000661	M	2.11
2,2',3',4,4',5-hexachlorobiphenyl	C029790	M	2.08
arsenic disulfide	C058317	M	2.08
Hexachlorocyclohexane	D001556	M	2.08
Sodium Fluoride	D012969	M	2.07
Diethylstilbestrol	D004054	M	2.02
Polycyclic Aromatic Hydrocarbons	D011084	M	1.90
Valproic Acid	D014635	M | T	24.15
Estradiol	D004958	M | T	8.43

Plant Preparations	D028321	M | T	6.04
Ketamine	D007649	M | T	4.70
Chlordiazepoxide	D002707	M | T	3.70
Phenobarbital	D010634	M | T	3.25
Caffeine	D002110	M | T	3.10
Nitrous Oxide	D009609	M | T	2.95
Haloperidol	D006220	M | T	2.61
Tadalafil	D000068581	T	21.31
Melatonin	D008550	T	19.37
icariin	C056599	T	17.29
puerarin	C033607	T	14.68
fisetin	C017875	T	13.07
huperzine A	C050426	T	12.79
Donepezil	D000077265	T	12.14
pimagedine	C004479	T	11.89
Selegiline	D012642	T	11.82
Physostigmine	D010830	T	11.7
Serotonin	D012701	T	11.27
crocin	C029036	T	10.55
Coconut Oil	D000074263	T	10.00
gastrodin	C045345	T	9.89
Amino Acids	D000596	T	9.82
ginsenoside Rg1	C035054	T	9.55
1-(3-(4-(3-chlorophenyl)-1-piperazinyl)propyl)-3,4-dihydro-5-methoxy-2(1H)-quinolinone	C404207	T	9.52
notoginsenoside R1	C072936	T	9.52
Taurine	D013654	T	9.32
Acetylcysteine	D000111	T	9.25
Methylene Blue	D008751	T	9.14
Clozapine	D003024	T	8.87
tetramethylpyrazine	C017953	T	8.38

Curcumin	D003474	T	8.16
1'-acetoxychavicol acetate	C047948	T	8.14
timosaponin AIII	C543146	T	8.11
lomatin	C540381	T	8.04
Propolis	D011429	T	8.02
Memantine	D008559	T	7.86
ferulic acid	C004999	T	7.56
Tacrine	D013619	T	7.46
4-(5-benzo(1,3)dioxol-5-yl-4-pyridin-2-yl-1H-imidazol-2-yl)benzamide	C459179	T	7.45
2-(4-((dimethylamino)methyl)benzylidene)-5,6-dimethoxy-2,3-dihydroinden-1-one	C543150	T	7.43
Galantamine	D005702	T	7.34
peoniflorin	C015423	T	7.29
Metformin	D008687	T	7.23
Saponins	D012503	T	7.12
Rivastigmine	D000068836	T	7.08
albicanol	C438910	T	7.02
Dehydroepiandrosterone	D003687	T	6.91
mangiferin	C013592	T	6.49
Metyrapone	D008797	T	6.42
caffeic acid	C040048	T	6.27
Alendronate	D019386	T	5.78
Ondansetron	D017294	T	5.75
gypenoside	C474918	T	5.58
hyperforin	C001654	T	5.06
4-(benzodioxan-5-yl)-1-(indan-2-yl)piperazine	C079549	T	4.85
SA 4503	C101789	T	4.76
Dimercaprol	D004112	T	4.70
Butyric Acid	D020148	T	4.69

Risperidone	D018967	T	4.67
igmesine	C065310	T	4.56
spiro(imidazo-(1,2-a)pyridine-3,2-indan)-2(3H)-one	C511224	T	4.51
phenserine	C092280	T	4.3
Rosiglitazone	D000077154	T	3.93
Butylated Hydroxyanisole	D002083	T	3.88
Succimer	D004113	T	3.62
Ergothioneine	D004880	T	3.47
Ginkgo biloba extract	C063170	T	3.45
Topiramate	D000077236	T	3.44
Canagliflozin	D000068896	T	3.30
Piracetam	D010889	T	3.25
Plant Extracts	D010936	T	3.17
Oxotremorine	D010095	T	3.15
protopanaxadiol	C062916	T	3.12
Huang Qi	C027492	T	3.03
Pramipexole	D000077487	T	2.92
Ethosuximide	D005013	T	2.87
HU 308	C402416	T	2.65
Quercetin	D011794	T	2.37
Choline	D002794	T	2.25
Lamotrigine	D000077213	T	2.22

S1.2.7 Branches of Motor Skills Disorders

A total of 60 chemicals were identified for motor skills disorders, with 45 associated with markers/mechanisms, 14 associated with therapeutics, and one linked to both categories.

Supplementary Table S8. Chemical-Gene Interactions for Motor Skills Disorders

Chemical Name	Chemical ID	Direct Evidence	Inference Score
**Motor Skills Disorders (MESH: D019957)**			
bisphenol A	C006780	M	12.41
1-Methyl-4-phenyl-1,2,3,6-tetrahydropyridine	D015632	M	8.41
Uranyl Nitrate	D014502	M	7.07
Manganese	D008345	M	5.27
Pyridostigmine Bromide	D011729	M	4.79
Aluminum Chloride	D000077410	M	4.50
Toluene	D014050	M	4.01
Streptozocin	D013311	M	3.87
Atrazine	D001280	M	3.8
Thioacetamide	D013853	M	3.78
Porphyrins	D011166	M	3.76
Paclitaxel	D017239	M	3.70
Herbicides	D006540	M	3.58
Lithium	D008094	M	3.49
Polychlorinated Biphenyls	D011078	M	3.36
Cannabidiol	D002185	M	3.27
Dizocilpine Maleate	D016291	M	3.27
fenvalerate	C017690	M	3.24
Clozapine	D003024	M	3.23
cypermethrin	C017160	M	3.23
Propofol	D015742	M	3.17
Phenytoin	D010672	M	3.05
Maneb	D008344	M	3.03
Cadmium Chloride	D019256	M	2.97
Lead	D007854	M	2.85
Vincristine	D014750	M	2.75
Paraquat	D010269	M	2.68
Risperidone	D018967	M	2.64

Sevoflurane	D000077149	M	2.58
Carboplatin	D016190	M	2.56
Cocaine	D003042	M	2.42
manganese chloride	C025340	M	2.40
Rotenone	D012402	M	2.40
Tretinoin	D014212	M	2.40
Ethanol	D000431	M	2.34
Pentachlorophenol	D010416	M	2.30
2',3,3',4',5-pentachloro-4-hydroxybiphenyl	C111118	M	2.18
Arsenic	D001151	M	2.15
Caffeine	D002110	M	2.15
Cisplatin	D002945	M	2.15
Methamphetamine	D008694	M	2.03
Endosulfan	D004726	M	1.96
Chlorpyrifos	D004390	M	1.95
Dronabinol	D013759	M	1.93
Copper	D003300	M	1.91
Valproic Acid	D014635	M | T	4.66
Trehalose	D014199	T	6.89
naringenin	C005273	T	6.23
geraniol	C007836	T	5.98
N-(oxo-5,6-dihydrophenanthridin-2-yl)-N,N-dimethylacetamide hydrochloride	C434926	T	4.73
Rimonabant	D000077285	T	3.88
benserazide, levodopa drug combination	C005177	T	3.80
Butyric Acid	D020148	T	3.77
beta-N-methylamino-L-alanine	C001824	T	3.67
Menthol	D008610	T	3.66
temsirolimus	C401859	T	3.37
Plant Extracts	D010936	T	3.29

Methylphenidate	D008774	T	3.09
6-hydroxy-2,5,7,8-tetramethylchroman-2-carboxylic acid	C010643	T	2.97
Glyburide	D005905	T	2.79

S1.3 Chemical Pollutants Curated Independently of the Comparative Toxicogenomics Database

To identify target chemical pollutants, we adopted two paradigms: 1) an evidence-based approach to select pollutants with established meta-analytic associations with neurodevelopmental disorders, aimed at validating the reliability of pollutant-disease associations in the CTD, and 2) a public health-focused approach to explore broader potential associations between chemical pollutants and neurodevelopmental disorders.

Evidence-Based Chemical Pollutants

We conducted an umbrella systematic review to identify meta-analyses on the association between chemical pollutants and neurodevelopmental disorders.[3] Comprehensive searches were conducted in PubMed, Embase, PsycINFO, and the Cochrane Database of Systematic Reviews, covering publications from the inception of the databases to December 2023. A combination of free-text terms with truncation and subject headings, including *environmental pollutants*, *neurodevelopmental disorders*, and *meta-analyses*, was used. When multiple meta-analyses focused on the same research topic, the most recent meta-analysis with the largest number of individual studies was included. We identified meta-analyses with/without systematic review addressing exposures to chemical pollutants across key developmental windows, from preconception to childhood, in relation to neurodevelopmental disorders.[3] Pollutants identified through this approach were designated as E (*evidence from the meta-analyses*).

Chemical Pollutants That Constitute Public Health Concerns

To identify pollutants posing current public health threats, we focused on review articles examining chemical pollutants and their effects on human health. This approach extended beyond chemicals linked to neurodevelopmental disorders.[4-6] Given the longer follow-up periods required for neurodevelopmental studies compared to reproductive and pregnancy-related research, a lack of evidence in neurodevelopmental contexts does not imply an absence of potential effects. Instead, pollutants implicated in other health-related studies may also pose risks to fetal and childhood health. Pollutants identified through this approach were designated as P (*public health concerns*).

Supplementary Table S9. Data Availability for Identified Chemical Pollutants

Chemicals	MeSH® ID	Identification Approach	Data Status
Air Pollutants	D000393	E | P	
Particulate Matter	D052638	E | P	
Nitrogen Oxides	D009589	E | P	
Sulfur Oxides	D013461	E | P	

Carbon Monoxide	D002248	E | P
Ozone	D010126	E | P
Polycyclic Aromatic Hydrocarbons	D011084	E | P
Phenanthrene	C031181	P
Pyrene	C030984	P
Lead	D007854	E | P
Mercury	D008628	E | P
Cadmium	D002104	E | P
Manganese	D008345	E | P
Antimony	D000965	E | P
Barium	D001464	E | P
Cobalt	D003035	E | P
Nickel	D009532	E | P
Zinc	D015032	E | P
Copper	D003300	E | P
Iron	D007501	E | P
Aluminum	D000535	E | P
Lithium	D008094	E | P
Magnesium	D008274	E | P
Arsenic	D001151	E | P
Pesticides	D010575	E | P
Fungicides, Industrial	D010575	E | P
Herbicides	D006540	E | P
Insecticides	D007306	E | P
Organophosphates	D010755	E | P
Hydrocarbons, Chlorinated	D006843	E | P
Carbamates	D002219	E | P
Pyrethrins	D011722	E | P

Hexachlorobenzene	D006581	E | P
Glyphosate	D000097797	P
DEET	D003671	P
DDT	D003634	P
Dichlorodiphenyl Dichloroethylene	D003633	P
Parathion	D010278	P
Methyl parathion	D008743	P
Dieldrin	D004026	P
Aldrin	D000452	P
Triclosan	D014260	P
Fluorocarbons	D005466	E | P
perfluorooctanoic acid	C023036	E | P
perfluorooctane sulfonic acid	C076994	E | P
perfluorodecanoic acid	C036567	E | P
perfluoro-n-nonanoic acid	C101816	E | P
perfluorohexanesulfonic acid	C471071	E | P
Polychlorinated Biphenyls	D011078	E | P
2,3',4,4',5-pentachlorobiphenyl	C070055	E | P
2,2',3',4,4',5-hexachlorobiphenyl	C029790	E | P
2,4,5,2',4',5'-hexachlorobiphenyl	C014024	E | P
2,2',3,3',4,4',5-heptachlorobiphenyl	C541131	E | P
Halogenated Diphenyl Ethers	D055768	E | P
Dibutyl Phthalate	D003993	E | P
Diethylhexyl Phthalate	D004051	E | P
diethyl phthalate	C007379	E | P
Plasticizers	D010968	E | P
bisphenol A	C006780	E | P
bisphenol B	C492482	P

bisphenol S	C543008	P	
oxybenzone	C005290	P	
Plastics	D010969	P	
Microplastics	D000080545	P	
Chloroform	D002725	P	
Methylene chloride	D008752	P	
Tetrachloroethylene	D013750	P	
Trichloroethylene	D014241	P	
Carbon Tetrachloride	D002251	P	

Note: *indicates the data of associated genes,* *indicates the data of associated diseases,* *indicates the data of associated phenotypes, and* *indicates the data of associated exposure references.* [a] The chemical-gene interaction value for the APP gene associated with aluminum was exceedingly high at 1821. As a result, the 1% threshold included only two genes. Given that the second-highest gene value was 21.0 and the third-highest was 18.0, we adopted a more stringent threshold of 0.01% for aluminum.

S1.4 Overlap Between CTD-Identified Pollutants and Independently Curated Pollutants

Based on chemical-disease interactions for seven neurodevelopmental disorders, 385 unique chemicals were identified from CTD, including 82 classified as pollutants. Of these, 33 chemical pollutants overlapped with the 69 pollutants curated independently of the CTD. To address differences in classification granularity—where common chemical pollutants represent broader substance categories and CTD pollutants primarily consist of specific congeners—CTD pollutants were consolidated into broader categories (e.g., PBC128 and PBC180 were grouped under PBCs, and manganese chloride under manganese ions). After clustering, CTD pollutants encompassed 61 substances, while common environmental pollutants included 63 substances, resulting in an overlap rate exceeding 50%.

Supplementary Figure S1. Overlap of Chemicals between the CTD-Identified Pollutants and Independently Curated Chemical Pollutants

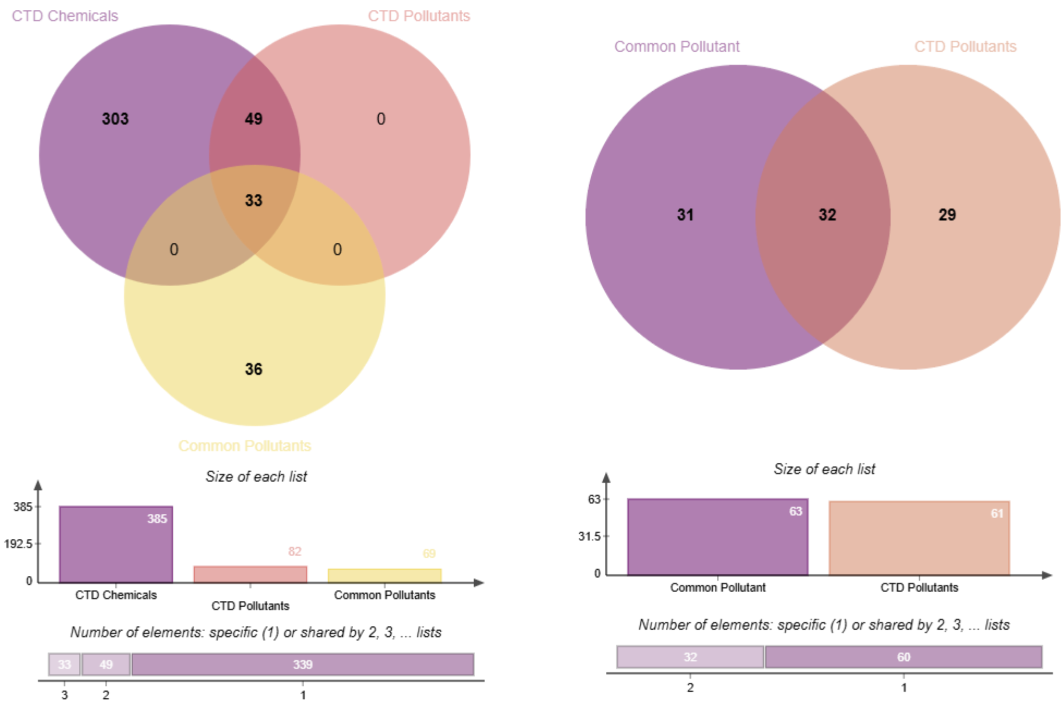

Supplementary Table S10. Overlap and Reclassification of Chemical Pollutants

Gene Sets	MeSH® ID	Chemical–Gene–Disease Networks	Common Environmental Pollutants	Clustering
**Air Pollutants**				
Air Pollutants	D000393	√	√	Included
Carbon Monoxide	D002248		√	Included
Carbon Disulfide	D002246	√		Included
Dibenzofurans	D000072318	√		Included
Nitrogen Oxides	D009589		√	Included
Ozone	D010126		√	Included
Particulate Matter	D052638	√	√	Included
Sulfur Oxides	D013461		√	Included
Sulfur Dioxide	D013458	√		-
Tobacco Smoke Pollution	D014028	√		Included
Vehicle Emissions	D001335	√		Included
Polycyclic Aromatic Hydrocarbons	D011084	√	√	Included
Phenanthrene	C031181	√	√	-
Pyrene	C030984		√	-
Benzo(a)pyrene	D001564	√		-
**Toxic and Trace Elements**				
Aluminum	D000535	√	√	Included
Antimony	D000965		√	Included
Arsenic	D001151	√	√	Included
Sodium Arsenite	C017947	√		-
Arsenic Disulfide	C058317	√		-
Arsenic Trioxide	D000077237	√		-
Barium	D001464		√	Included

Cadmium	D002104	√	√	Included
Cadmium Chloride	D019256	√		-
Cobalt	D003035	√	√	Included
Copper	D003300	√	√	Included
Iron	D007501	√	√	Included
Lead	D007854	√	√	Included
Lead Acetate	C008261	√		-
Lithium	D008094	√	√	Included
Magnesium	D008274	√	√	Included
Manganese	D008345	√	√	Included
Manganese Chloride	C025340	√		-
Manganese Sulfate	C039798	√		-
Mercury	D008628	√	√	Included
Methylmercuric Chloride	C004925	√		-
Mercuric Oxide	C019468	√		-
Nickel	D009532		√	Included
Tributyltin	C011559	√		Included
Uranyl Nitrate	D014502	√		Included
Zinc	D015032	√	√	Included
**Pesticides and Related Compounds**				
Pesticides	D010575	√	√	Included
Aldrin	D000452		√	Included
Atrazine	D001280	√		Included
Carbamates	D002219		√	Included
Chlorpyrifos	D004390	√		Included
Chlorobenzene	C031294	√		Included
4-Dichlorobenzene	C018511	√		-
Hexachlorobenzene	D006581		√	-

DDT	D003634	√	√	Included
Dieldrin	D004026		√	Included
Dichlorodiphenyl Dichloroethylene	D003633	√	√	Included
Dichlorvos	D004006	√		Included
Dimethoate	D004117	√		Included
Endosulfan	D004726	√		Included
Fungicides, Industrial	D005659		√	Included
Glyphosate	D000097797	√	√	Included
Herbicides	D006540	√	√	Included
Hydrocarbons, Chlorinated	D006843		√	Included
Insecticides	D007306		√	Included
Malathion	D008294	√		Included
Methyl Parathion	D008743		√	Included
Organophosphates	D010755		√	Included
Paraquat	D010269	√		Included
Parathion	D010278	√	√	Included
Permethrin	D026023	√		Included
Pyrethrins	D011722	√	√	Included
Rotenone	D012402	√		Included
Sarin	D012524	√		Included
**Synthetic Organic Chemicals**				
1,3-Butadiene	C031763	√		Included
1,4-Dioxane	C025223	√		Included
Benzidine	C029876	√		Included
Bisphenol A	C006780	√	√	Included
Bisphenol B	C492482		√	Included
Bisphenol S	C543008		√	Included
Butylbenzyl Phthalate	C027561	√		Included

DEET	D003671		√	Included
Dibutyl Phthalate	D003993		√	Included
Diethyl Phthalate	C007379		√	Included
Diethylhexyl Phthalate	D004051	√	√	Included
Fluorocarbons	D005466		√	Included
Halogenated Diphenyl Ethers	D055768		√	Included
2,2',4,4',5-Brominated Diphenyl Ether	C477694	√		-
2,2',4,4'-Tetrabromodiphenyl Ether	C511295	√		-
Decabromobiphenyl Ether	C010902	√		-
Hexabromocyclododecane	C089796	√		Included
Hexachlorocyclohexane	D001556	√		Included
Methyl tert-butyl ether	C043243	√		Included
Microplastics	D000080545		√	Included
Oxybenzone	C005290		√	Included
PCB 180	C410127	√		-
Pentachlorophenol	D010416	√		Included
Perfluoro-n-Nonanoic Acid	C101816		√	Included
Perfluorodecanoic Acid	C036567		√	Included
Perfluorohexanesulfonic Acid	C471071	√	√	Included
Perfluorooctane Sulfonic Acid	C076994	√	√	Included
Perfluorooctanoic Acid	C023036	√	√	Included
Phthalic Acids	D010795	√		Included
Plasticizers	D010968		√	Included
Plastics	D010969		√	Included
Polychlorinated Biphenyls	D011078	√	√	Included
Chlorodiphenyl (54% chlorine)	D020111	√		-
2,2',3,3',4,4',5-Heptachlorobiphenyl	C541131		√	-
2,2',3',4,4',5-Hexachlorobiphenyl	C029790	√	√	-

2,3',4,4',5-Pentachlorobiphenyl	C070055	√	√	-
2,3,3',4,4',5-Hexachlorobiphenyl	C087667	√		-
2,4,5,2',4',5'-Hexachlorobiphenyl	C014024	√	√	-
3,4,5,3',4'-Pentachlorobiphenyl	C023035	√		-
Triclosan	D014260		√	Included
**Solvents**				
3-Xylene	C031285	√		Included
4-Xylene	C031286	√		Included
Carbon Tetrachloride	D002251		√	Included
Chloroform	D002725		√	Included
Formaldehyde	D005557	√		Included
Methylene Chloride	D008752		√	Included
Tetrachloroethylene	D013750	√	√	Included
Toluene	D014050	√		Included
Trichloroethylene	D014241		√	Included

S1.5 Gene Sets for Neurodevelopmental Disorders and Independently Identified Chemical Pollutants

S1.5.1 Gene Sets of Neurodevelopmental Disorders

The CTD utilizes a cross-species gene vocabulary, including symbols, names, and synonyms, derived from the National Center for Biotechnology Information (NCBI) gene database. We selected genes with curated associations (M *marker/mechanism and/or* T *therapeutic*) to neurodevelopmental disorders as unique gene sets for each disorder (date of download 10/31/2024).

Supplementary Table S11. Gene Sets for Neurodevelopmental Disorders

Neurodevelopmental Disorders	MeSH® ID	Number of Curated Genes	Curated Associations	Gene Sets
Attention Deficit Disorder with Hyperactivity	D001289	22	M	DRD2|SLC6A3|COMT|GRM5|DRD4|CNR1|GRM1|TPH2|CHRNA4|DRD5|GRM7|CIC|AS3MT|ADGRL3|CHRNB2|FGD1|GIT1|GRM8|PTPRD|STS|TACR1|CALY
Autism Spectrum Disorder	D000067877	359	M	AOX1|DNMT3A|DNMT1|CYP11A1|GSTM1|CYP2R1|DNMT3B|NOS1|FLT1|BDNF|KIFC1|HSD3B1|NRXN2|CYP7A1|EPHX1|ATP1B2|CYP27B1|FMO2|GFAP|CBS|ALDH1A1|ABCA2|SLC6A4|ALDH2|SCNN1A|CIRBP|GSTO1|TFRC|SOX9|MET|GSTA2|TJP1|MTTP|GSTP1|TET1|SLC2A3|TXNRD1|NOS2|TPMT|DHRS1|HSD17B1|GPX2|HFE|GALNS|CRYZ|PTGS1|CP|NOTCH1|AOC2|GRIN2B|HGF|TET3|SCN1A|SLC3A2|SLCO2A1|NQO1|IQGAP3|DLG4|ALDH3B1|INSR|COMT|AVPR1A|GSTT2|FOXP1|ALDH3A2|LRP1|MTR|AKR1C3|ITPR3|RAI1|OXTR|SHANK3|ALAD|CYP24A1|NRXN1|SLC16A2|ARSB|MEF2C|DIPK2A|DPP6|CYP19A1|SLC25A20|KIF17|SLC25A25|ARSI|DRD4|SULF2|CIC|LAMC3|SULT1B1|HEY1|B3GAT2|CYP26B1|PAOX|AQP4|CHAT|GPX6|ALDH1A3|CYP2U1|LRRN3|UPP2|ALDH4A1|ITGB3|SULF1|FMO3|NOTCH4|CYP4B1|SLC19A1|EBI3|SLC22A5|ADH6|CGN|DIO2|AQP9|ALDH5A1|ALDH6A1|CHST6|CYP4F2|GSTM5|CGNL1|SLC1A4|LOXL2|BCHE|AKR1B10|FMO4|VCL|SYN1|CHST12|CYP2S1|CA2|PON2|VWF|AKR1C2|GSTA3|CYP27A1|AHR|UGT1A1|PAPPA2|CYP1A1|ADSL|ACHE|ANKRD11|CNTNAP2|DNAAF2|CHD2|PTGIS|ALDH3A1|NTSR1|PCDH9|PTGES2|DIO3|NOTCH2|CFTR|MIF|FMO1|ALOX15|SIN3A|CBR3|SOX5|LOXL4|SLCO1C1|ABCG2|EPHX2|

<table>
<tr><td></td><td></td><td></td><td></td><td>ODAD2|TPST2|DHRS2|SLC1A1|ABCC3|FBN2|MAGEA8|CYP3A7|SULT2B1|RELN|FOXP2|CYP3A5|GAL3ST3|IL1RL1|ATP1A3|ABCB1|UGT2B10|PTGR1|ALDH3B2|CHST4|ADH1B|APC|SULT1C2|GAL3ST1|JAM3|FOLR2|CYP1A2|POMT1|SLC2A14|CYP2D6|NQO2|HTRA4|PROK1|NOS3|SLC13A4|ADRB2|PTGES|PRKCZ|ADH4|DPP10|UGT1A6|MTHFR|SLC16A1|CHST8|CHST13|CYP2C9|NAT2|PRG2|MPDZ|RARB|DHCR24|CYP2J2|LOXL1|ALOX12|ADA|JAM2|CYP39A1|TJP3|PON3|ABCA8|GNGT1|ALPP|GTF2I|PARD6B|TP53I3|CES2|NSUN2|CYP4X1|DDHD1|CYP21A2|CYP2B6|SFSWAP|CYP11B2|RYR2|JARID2|RSPH4A|CYP2C19|ALDH1L1|CHST11|DNAH7|SLC12A7|CEP41|TSHZ3|UGT2B7|AKR1C4|IL1RAPL1|TXNRD2|GPR32|CLDN3|SCNN1B|SUOX|ALOXE3|NOTCH3|TJP2|SNTG2|GPX5|ARSK|AKR1A1|CHST14|UGT2A3|CBR1|CHST10|USH2A|ADH5|CHST3|NOTUM|PSG4|UNC80|NEMF|GABRB3|ARSG|DIP2A|PTGES3|TPST1|ALOX12B|UGT2B11|DNAI1|PAH|SCNN1G|CTTNBP2|PARD3|CYP4F22|TAC3|ROGDI|LRRTM3|DPYD|JAG1|CHST5|CYP4F12|DLX1|UGT2A1|DIPK2B|AOC1|CCDC39|EN2|DNAI2|ADAMTS18|CYP4A11|DNAAF1|PSG9|CYP3A43|DNAH5|CYP4A22|MTNR1A|UGT2B15|ODAD1|IFT74|GUSB|POMT2|AKR7A3|CHD8|UGT2B4|GPR50|PTCHD1|GABRA5|GH2|STS|AFDN|CGB1|CES3|CYP2C8|SGF29|UGT1A8|ADH1A|DNAH11|CDH10|PSG1|AKR7A2|CYP27C1|ALOX15B|ARSE|CCDC40|ATP1A4|MTNR1B|UGT1A10|PSG7|ARSH|CSH1|NLGN4X|FEV|CYP4F8|TBL1X|CYP2A7|CYP2F1|SULT1C3|ARSF|PSG6|LGALS13|LGALS16|XAGE3|PSG3|MIR486-1|CYP2A13|LGALS14|MIR181B-1|TRIM64B</td></tr>
<tr><td>Autistic Disorder</td><td>D001321</td><td>257</td><td>M | T</td><td>CAT|BDNF|PRL|IL10|HTR2A|IFNG|BCL2|IL6|AQP4|PTGS2|GPX1|POMC|MAOA|IGF1|DRD1|GSTM1|GAD1|NOS2|SLC6A4|PON1|GRIN2A|NTRK2|MAPK3|IL4|SERPINE1|IL2|DHCR7|LEP|GJA1|MAOB|MTHFR|DLGAP2|CALCA|PRLR|GABBR2|VLDLR|PTEN|GSTP1|IL13|PLA2G4A|PDE4A|DRD3|IGF2|PRKCB|COMT|NRXN1|ADM|TDO2|DAB1|MECP2|AVP|RELN|EIF4E|FOXP2|SND1|NLGN3|MIF|CACNA1C|PDE4B|MET|SLC19A1|MEF2C|CP|IGFBP3|CHUK|HRAS|HTR1B|ITGA4|JAKMIP1|EGF|PRKN|SLC40A1|EGR2|C4B|PLAUR|SEZ6L2|PER1|GHR|JMJD1C|T</td></tr>
</table>

<table>
<tr><td></td><td></td><td></td><td></td><td>AF1C|ST8SIA2|GABRA1|TPH2|ADRB2|PRF1|NAV3|XPC|TNFRSF1B|IL5|ITPR3|XDH|SCAMP5|TF|PAX6|PIK3CG|NPAS2|DLX2|ABAT|PECAM1|GRIK2|ADA|MCC|EIF4G1|KIF1A|SHANK3|NRP2|UBE2H|NF1|WDFY4|GPR155|ROBO2|FOXP1|DOCK4|EXT1|MTF1|DLX1|LAMB1|TCN2|GABRA5|RBFOX1|ATP10A|SLC1A3|MARK1|CACNA1H|GRIA1|GZMB|FBXO40|AR|CHRNA7|ADSL|AGAP1|PASK|RORA|CNTNAP2|IL1RN|LZTR1|SLC9A9|NTF3|ACE|APC|SLC6A8|TSC2|AVPR1A|ROBO3|KDM5C|STK39|HNRNPUL2|MAGEL2|RPL10|RNF8|GABRA3|ACADSB|SCN7A|HTR5A|ZMYND11|HTR1D|VWA8|SHANK1|CDH10|NSD1|HTN1|NLGN1|PCDH10|OXTR|ASTN2|MARK2|GABRB3|MBD3|CADM1|SHANK2|INPP1|ITGB3|LASP1|NRCAM|SLC25A12|DPYD|PRKCI|GLO1|FARP2|CHRNA4|CHD4|UBE3A|SCN2A|PLD5|GABRA2|TSC1|DHFR|UPB1|SCAF1|ITSN1|SEMA5A|RAB11FIP5|HOXA1|CYP19A1|RIMS1|GABRB1|ASIC2|FMR1|GABBR1|REEP3|PTCHD1|BTD|EN2|HLA-A|STXBP6|TBR1|NTF4|KCNMA1|MIR106B|VIP|CADPS2|ADNP|IL15|MIR23A|NBEA|HDLBP|CUL7|CHRNB2|RAB39B|TMLHE|ROBO1|ROBO4|SYNGAP1|IMMP2L|SNTG2|WNT2|SNRPN|DIXDC1|HLA-DRB1|DAO|GABRA4|CSMD3|MYO1D|HTR3A|DIPK2A|CNTN3|CNTN4|IL1RAPL1|MACROD2|GRM8|AHI1|ASMT|NLGN4X|MBD4|STX1A|TYK2|COP1|CDH9|PITX1|CHD8|DISC1|POU6F2|HOXD11|HTR3C|STATH|OXT|SCT</td></tr>
<tr><td>Developmental Disabilities</td><td>D002658</td><td>31</td><td>M</td><td>MAPK3|SLC2A1|NTRK2|PTEN|DRD2|KCNQ2|SLC6A8|PMP22|SHANK3|CHRNA4|MECP2|SLC4A4|PNKP|TBCD|KIF1A|CBL|AS3MT|WDFY4|KCNT1|NANS|CHD4|LRP2|HSPB9|SLC33A1|DOCK8|CNTN4|HSPB11|CAMKMT|MSL3|STAMBP|ARFGAP1</td></tr>
<tr><td>Intellectual Disability</td><td>D008607</td><td>139</td><td>M</td><td>BDNF|DNMT3A|FASN|MED13L|PAX6|SC5D|NRXN1|ALDH5A1|ADK|SHANK2|SCN1A|KDM5A|GNAS|SIN3A|TH|SLC2A1|PRKCG|CIC|NAA15|CCNA2|FRY|RABL6|CACNA1G|HDAC4|AHI1|NSD1|FOLR1|PTEN|FGFR2|GAMT|PMM2|CASP2|GRIN2B|MEF2C|SRGAP3|PHIP|COL18A1|CAPN10|FMR1|UROC1|KDM5C|DYNC1H1|MECP2|SYNGAP1|MFSD2A|PEX6|SCAPER|LARP7|ENTPD1|EEF1B2|STRA6|PECR|DOCK8|PARP1|TAF2|CA8|AP4E1|TSEN54|WDR45B|PRRT2|COQ5|HY</td></tr>
</table>

				CC1|TMCO1|ELP2|RGS7|SLC31A1|CHD8|INPP4A|LETM1|KCNA2|FOXG1|DISC1|WDR62|CCBE1|VRK1|PRKRA|NDST1|TBCD|ASCC3|SCN8A|RAI1|H4C3|APC|SHANK3|SETBP1|HEXA|DCC|ARL14EP|PTCHD1|KDM6B|METTL23|KANSL1|ZCCHC8|CALCA|H3-4|SNX14|CNDP1|GON4L|CHL1|POLR3B|L2HGDH|TMEM135|SURF1|TRMT1|ZMYM2|PDHX|SRD5A3|NAGLU|RALGDS|MCC|ZNF526|TTI2|H4C2|ACBD6|C12ORF57|MAN1B1|KIFBP|INPP5E|NTF4|TSEN34|AP4M1|SLC4A10|UBR7|CNKSR1|ERLIN2|ZBTB40|RAB39B|DEAF1|KIF7|TSEN2|STAG1|ADRA2B|LAMA1|ASCL1|VIP|YY1|NF1|LINS1|BBS7
Learning Disabilities	D007859	33	M | T	BCL2|ACHE|IL1B|HMOX1|APP|TLR4|TH|MAPT|MT1|SYP|MT2|POR|GRIA1|PRKN|SLC17A7|PSEN1|SLC17A6|KL|MECP2|HTR1A|HTR7|APOD|NF1|PDE1B|PNOC|MICU1|CAMKMT|IGF1|VEGFA|TRH|IL1RN|SIGMAR1|MIR124A-3
Motor Skills Disorders	D019957	13	M | T	SOD1|SQSTM1|PTEN|OGG1|NDUFS4|FGFR2|SHANK1|DISC1|AKAP5|CLN6|CAMKMT|CNR1|RPTOR

S1.5.2 Gene Sets of Chemical Pollutants

Integrating the identified pollutants, we categorized all pollutants using the MeSH-based chemical classification and extracted their associated gene sets from CTD. To enhance analytical precision and mitigate biases from disproportionately large gene sets, we excluded genes with chemical–gene or chemical–protein interaction values below 1% of the maximum interaction value for each chemical. The CTD infers binary chemical–gene and chemical–protein interactions from published literature, encompassing both vertebrates and invertebrates.

Supplementary Table S12. Gene Sets for Identified Chemical Pollutants

Chemicals	MeSH® ID	Number of Genes	Interaction Cutoff Values
**Air Pollutants**			
Air Pollutants	D000393	4115	1.06
Particulate Matter	D052638	1731	5.97
Nitrogen Oxides	D009589	80	3.08
Sulfur Oxides	D013461	299	0.15
Carbon Monoxide	D002248	112	0.38

Ozone	D010126	4374	1.62
Polycyclic Aromatic Hydrocarbons	D011084	476	29.30
Phenanthrene	C031181	214	0.16
Pyrene	C030984	297	0.29
**Toxic and Trace Elements**			
Lead	D007854	3448	0.59
Mercury	D008628	655	0.41
Cadmium	D002104	1566	1.59
Manganese	D008345	583	0.43
Antimony	D000965	221	0.13
Barium	D001464	82	0.23
Cobalt	D003035	251	3.90
Nickel	D009532	3192	1.36
Zinc	D015032	2702	0.88
Copper	D003300	441	3.74
Iron	D007501	458	0.60
Aluminum	D000535	85	1.82 [a]
Lithium	D008094	256	0.24
Magnesium	D008274	232	0.11
Arsenic	D001151	991	1.01
**Pesticides and Related Compounds**			
Pesticides	D010575	1098	0.39
Fungicides, Industrial	D010575	30	0.02
Herbicides	D006540	413	0.09
Insecticides	D007306	45	0.11
Organophosphates	D010755	157	13.43
Hydrocarbons, Chlorinated	D006843	5748	4.95
Carbamates	D002219	1029	2.77

Pyrethrins	D011722	3115	1.11
Hexachlorobenzene	D006581	232	0.30
Glyphosate	D000097797	3144	0.32
DEET	D003671	1405	0.10
DDT	D003634	348	1.07
Dichlorodiphenyl Dichloroethylene	D003633	2769	0.51
Parathion	D010278	1274	0.82
Methyl parathion	D008743	101	0.53
Dieldrin	D004026	1584	0.23
Aldrin	D000452	225	0.06
Triclosan	D014260	3427	0.22
**Synthetic Organic Chemicals**			
Fluorocarbons	D005466	1525	5.97
perfluorooctanoic acid	C023036	381	3.35
perfluorooctane sulfonic acid	C076994	3777	1.03
perfluorodecanoic acid	C036567	252	0.38
perfluoro-n-nonanoic acid	C101816	2237	0.35
perfluorohexanesulfonic acid	C471071	2716	0.19
Polychlorinated Biphenyls	D011078	2147	3.73
2,3',4,4',5-pentachlorobiphenyl	C070055	1331	0.22
2,2',3',4,4',5-hexachlorobiphenyl	C029790	2107	0.09
2,4,5,2',4',5'-hexachlorobiphenyl	C014024	2382	0.56
2,2',3,3',4,4',5-heptachlorobiphenyl	C541131	31	0.02
Halogenated Diphenyl Ethers	D055768	8499	0.55
Dibutyl Phthalate	D003993	6081	0.58
Diethylhexyl Phthalate	D004051	252	5.46
diethyl phthalate	C007379	341	0.25
Plasticizers	D010968	98	0.02

bisphenol A	C006780	238	19.76
bisphenol B	C492482	1758	0.23
bisphenol S	C543008	19	9.96
oxybenzone	C005290	336	0.13
Plastics	D010969	512	0.40
Microplastics	D000080545	35	0.04
**Solvents**			
Chloroform	D002725	155	0.09
Methylene chloride	D008752	220	0.04
Tetrachloroethylene	D013750	867	0.04
Trichloroethylene	D014241	6820	0.83
Carbon Tetrachloride	D002251	1122	3.27

S1.6 Mapping Gene Sets onto the Homo sapiens Genome

To ensure that the generated genes are derived exclusively from the human genome, we mapped all obtained gene sets, including those associated with diseases and pollutants. Since some gene evidence related to chemicals may originate from multiple species, such as mice and rats, we ensured that subsequent enrichment analyses were based on the same human genome background, allowing for comparability of enrichment results across chemicals.

Background Reference Genes

We accessed the NCBI GENOME database to obtain genome annotation data for Homo sapiens (human), using version GRCh38.p14 (GCF_000001405.40) with annotation name GCF_000001405.40-RS_2024_08 (dated August 23, 2024). The annotated gene types included protein-coding, non-coding, tRNA, rRNA, pseudogene, and other. Pseudogenes were excluded, resulting in a total of 49,002 gene annotations. After removing duplicates, 42,854 unique genes were retained as background reference genes. Established neurodevelopmental disorder gene sets were cross-referenced with the Homo sapiens genome.

Supplementary Table S13. Gene Mapping of Gene Sets Associated with Neurodevelopmental Disorders and Identified Pollutants

Gene Sets	MeSH® ID	Number of Total Genes	Number of Mapped Genes	Percentage of Mapped Genes (%)
Attention Deficit Disorder with Hyperactivity	D001289	22	22	100
Autism Spectrum Disorder	D000067877	359	357	99.4
Autistic Disorder	D001321	257	257	100
Developmental Disabilities	D002658	31	30	96.8
Intellectual Disability	D008607	139	138	99.3
Learning Disabilities	D007859	33	30	90.9
Motor Skills Disorders	D019957	13	13	100
Air Pollutants	D000393	4115	3908	95.0
Particulate Matter	D052638	1731	1705	98.5
Nitrogen Oxides	D009589	80	78	97.5
Sulfur Oxides	D013461	299	288	96.3
Carbon Monoxide	D002248	112	110	98.2
Ozone	D010126	4374	4168	95.3
Polycyclic Aromatic Hydrocarbons	D011084	476	467	98.1

Phenanthrene	C031181	214	191	89.3
Pyrene	C030984	297	191	64.3
Lead	D007854	3448	3311	96.0
Mercury	D008628	655	608	92.8
Cadmium	D002104	1566	1524	97.3
Manganese	D008345	583	552	94.7
Antimony	D000965	221	219	99.1
Barium	D001464	82	80	97.6
Cobalt	D003035	251	248	98.8
Nickel	D009532	3192	3113	97.5
Zinc	D015032	2702	2471	91.5
Copper	D003300	441	422	95.7
Iron	D007501	458	418	91.3
Aluminum	D000535	85	84	98.8
Lithium	D008094	256	253	98.8
Magnesium	D008274	232	226	97.4
Arsenic	D001151	991	968	97.7
Pesticides	D010575	1098	966	88.0
Fungicides, Industrial	D005659	30	28	93.3
Herbicides	D006540	413	318	77.0
Insecticides	D007306	45	42	93.3
Organophosphates	D010755	157	152	96.8
Hydrocarbons, Chlorinated	D006843	5748	5263	91.6
Carbamates	D002219	1029	988	96.0
Pyrethrins	D011722	3115	2966	95.2
Hexachlorobenzene	D006581	232	204	87.9
Glyphosate	D000097797	3144	2731	86.9
DEET	D003671	1405	1233	87.8

DDT	D003634	348	314	90.2
Dichlorodiphenyl Dichloroethylene	D003633	2769	2610	94.3
Parathion	D010278	1274	1193	93.6
Methyl parathion	D008743	101	77	76.2
Dieldrin	D004026	1584	1424	89.9
Aldrin	D000452	225	208	92.4
Triclosan	D014260	3427	3187	93.0
Fluorocarbons	D005466	1525	1419	93.0
perfluorooctanoic acid	C023036	381	353	92.7
perfluorooctane sulfonic acid	C076994	3777	3376	89.4
perfluorodecanoic acid	C036567	252	214	84.9
perfluoro-n-nonanoic acid	C101816	2237	1960	87.6
perfluorohexanesulfonic acid	C471071	2716	2440	89.8
Polychlorinated Biphenyls	D011078	2147	1889	88.0
2,3',4,4',5-pentachlorobiphenyl	C070055	1331	1188	89.3
2,2',3',4,4',5-hexachlorobiphenyl	C029790	2107	1840	87.3
2,4,5,2',4',5'-hexachlorobiphenyl	C014024	2382	2028	85.1
2,2',3,3',4,4',5-heptachlorobiphenyl	C541131	31	31	100
Halogenated Diphenyl Ethers	D055768	8499	7155	84.2
Dibutyl Phthalate	D003993	6081	5292	87.0
Diethylhexyl Phthalate	D004051	252	239	94.8
diethyl phthalate	C007379	341	326	95.6
Plasticizers	D010968	98	97	99.0
bisphenol A	C006780	238	232	97.5
bisphenol B	C492482	1758	1731	98.5
bisphenol S	C543008	19	18	94.7
oxybenzone	C005290	336	282	83.9
Plastics	D010969	512	475	92.8

Microplastics	D000080545	35	32	91.4
Chloroform	D002725	155	115	74.2
Methylene chloride	D008752	220	181	82.3
Tetrachloroethylene	D013750	867	800	92.3
Trichloroethylene	D014241	6820	6091	89.3
Carbon Tetrachloride	D002251	1122	1060	94.5

By mapping disorder- and pollutant-associated gene sets to the human genome, we found that the mapping rates for disorder gene sets were consistently high, ranging from 90.9% to 100%, with a median of 99.4%. In comparison, the mapping rates for pollutant-associated gene sets were relatively lower, ranging from 64.3% to 100%, with a median of 93.0%. These findings indicate that the disease gene sets provided by CTD are broadly represented across the human genome, but the chemical gene sets may have potential species-related biases.

S1.7 Identified Pollutant-Disorder Gene Sets

Based on the enrichment analysis results, five neurodevelopmental disorders were found to be significantly enriched with pollutants, including autism spectrum disorder, autistic disorder, learning disabilities, intellectual disability, and developmental disabilities. The intersection of pollutant gene sets and neurodevelopmental disorder gene sets was calculated, excluding disorder genes not present in any pollutant gene set. A comparison of the significantly enriched pollutants with those identified by the CTD revealed that autism spectrum disorder, autistic disorder, and intellectual disability shared common pollutants. However, since autistic disorder is an outdated diagnostic category, it was excluded from subsequent analyses.

Supplementary Table S14. Pollutant-Disorder Gene Sets for Autism Spectrum Disorder (MESH: D000067877)

Chemical Name	Chemical ID	Gene Sets
Total Pollutants	-	ABCA2|ABCB1|ABCC3|ABCG2|ACHE|ADA|ADH1A|ADH1B|ADH4|ADH5|ADH6|ADRB2|ADSL|AFDN|AHR|AKR1A1|AKR1B10|AKR1C2|AKR1C3|AKR7A2|AKR7A3|ALAD|ALDH1A1|ALDH1A3|ALDH1L1|ALDH2|ALDH3A1|ALDH3A2|ALDH3B1|ALDH3B2|ALDH4A1|ALDH5A1|ALDH6A1|ALOX12|ALOX12B|ALOX15|ANKRD11|AOC1|AOC2|AOX1|APC|AQP4|AQP9|ARSG|ARSI|ARSK|ATP1A3|ATP1B2|AVPR1A|B3GAT2|BCHE|BDNF|CA2|CBR1|CBR3|CBS|CEP41|CES2|CES3|CFTR|CGN|CGNL1|CHAT|CHD2|CHD8|CHST10|CHST11|CHST3|CHST6|CHST8|CIC|CIRBP|CLDN3|CNTNAP2|COMT|CP|CRYZ|CTTNBP2|CYP11A1|CYP11B2|CYP19A1|CYP1A1|CYP1A2|CYP21A2|CYP24A1|CYP26B1|CYP27A1|CYP27B1|CYP27C1|CYP2B6|CYP2C19|CYP2C8|CYP2C9|CYP2D6|CYP2F1|CYP2J2|CYP2R1|CYP2S1|CYP2U1|CYP39A1|CYP3A5|CYP3A7|CYP4A11|CYP4A22|CYP4B1|CYP4X1|CYP7A1|DHCR24|DHRS1|DHRS2|DIO2|DIO3|DIP2A|DLG4|DNAAF2|DNAH11|DNAH7|DNAI2|DNMT1|DNMT3A|DNMT3B|DPP10|DPP6|DPYD|DRD4|EBI3|EN2|EPHX1|EPHX2|FLT1|FMO1|FMO2|FMO3|FMO4|FOLR2|FOXP1|FOXP2|GABRB3|GAL3ST1|GALNS|GFAP|GNGT1|GPX2|GPX5|GPX6|GRIN2B|GSTA2|GSTA3|GSTM1|GSTM5|GSTO1|GSTP1|GSTT2|GTF2I|GUSB|HEY1|HFE|HGF|HSD17B1|HSD3B1|HTRA4|IFT74|IL1RAPL1|IL1RL1|INSR|IQGAP3|ITGB3|ITPR3|JAG1|JAM2|JAM3|JARID2|KIF17|KIFC1|LAMC3|LOXL1|LOXL2|LOXL4|LRP1|LRRN3|LRRTM3|MAGEA8|MEF2C|MET|MIF|MPDZ|MTHFR|MTR|MTTP|NOS1|NOS2|NOS3|NOTCH1|NOTCH2|NOTCH3|NOTCH4|NQO1|NQO2|NRXN1|NRXN2|NSUN2|NTSR1|ODAD2|OXTR|PAH|PAPPA2|PARD3|PARD6B|PCDH9|PON2|PON3|PRG2|PRKCZ|PROK1|PSG1|PSG6|PTCHD1|PTGES|PTGES2|PTGIS|PTGR1|PTGS1|RAI1|RARB|RELN|ROGDI|RSPH4A|RYR2|SCN1A|SCNN1A|SCNN1G|SFSWAP|SHANK3|SLC12A7|SLC13A4|SLC16A1|SLC16A2|SLC19A1|SLC1A1|SLC1A4|SLC22A5|SLC25A20|SLC25A25|SLC2A3|SLC3A2|SLC6A4|SLCO1C1|SLCO2A1|SOX5|SOX9|STS|SULF1|SULF2|SULT1B1|SULT1C2|SULT1C3|SULT2B1|SUOX|SYN1|TET1|TET3|TFRC|TJP1|TJP2|TP53I3|TPMT|TPST2|TXNRD1|TXNRD2|UGT1A1|UGT1A10|UGT1A6|UGT2A3|UGT2B10|UGT2B7|UNC80|UPP2|USH2A|VCL|VWF

Air Pollutants	D000393	ABCC3|ABCG2|ADRB2|AFDN|AHR|AKR1C3|AKR7A2|ALDH1A1|ALDH2|ALDH3A1|ALDH5A1|ALDH6A1|ALOX12|ANKRD11|AOX1|AQP9|BDNF|CBR3|CBS|CEP41|CFTR|CHD2|CHST10|CIC|CIRBP|CLDN3|CP|CTTNBP2|CYP1A1|CYP27A1|CYP2D6|CYP4B1|DHCR24|DNMT1|DNMT3B|DPYD|EPHX1|EPHX2|FLT1|FMO1|FMO2|FOXP1|GALNS|GFAP|GPX2|GSTA2|GSTA3|GSTM1|GSTO1|GSTP1|GUSB|HEY1|HFE|IL1RL1|IQGAP3|KIFC1|LAMC3|LOXL2|LRP1|LRRN3|MEF2C|MIF|MPDZ|MTHFR|MTTP|NOS1|NOS2|NOS3|NOTCH1|NOTCH2|NOTCH3|NOTCH4|NQO1|NRXN1|NRXN2|PARD6B|PCDH9|PRG2|PTGES|PTGIS|PTGR1|PTGS1|SHANK3|SLC13A4|SLC2A3|SLC3A2|SULT1B1|SYN1|TET3|TFRC|TJP2|TPMT|TXNRD1|VWF
Particulate Matter	D052638	ABCB1|ABCC3|ABCG2|ADRB2|AHR|AKR1B10|AKR1C2|AKR1C3|ALDH1A1|ALDH1A3|ALDH2|ALDH3A1|ALDH3B1|AOX1|APC|AQP4|BDNF|CBR1|CBR3|CFTR|CGN|CIRBP|COMT|CP|CYP11A1|CYP1A1|CYP1A2|CYP26B1|CYP2B6|CYP2S1|CYP39A1|CYP3A5|CYP4B1|DHCR24|DIO2|DLG4|DNAI2|DNMT1|DNMT3A|DNMT3B|EPHX1|EPHX2|FLT1|FMO2|GABRB3|GPX2|GSTA2|GSTA3|GSTM1|GSTO1|GSTP1|IL1RL1|ITGB3|JAG1|JARID2|LOXL1|LOXL2|MEF2C|MET|MIF|NOS1|NOS2|NOS3|NOTCH1|NOTCH2|NOTCH3|NOTCH4|NQO1|NRXN1|PTGES|PTGR1|PTGS1|SCNN1A|SHANK3|SLC1A4|SLC2A3|SLC3A2|SYN1|TET1|TFRC|TJP1|TXNRD1|TXNRD2|UGT1A1|UGT1A6|VCL|VWF
Lead	D007854	ABCA2|ABCB1|ABCC3|ACHE|ADH1B|ADH4|ADSL|AHR|AKR1B10|AKR1C2|ALAD|ALDH2|ALDH3A2|ALDH5A1|AQP4|ATP1A3|ATP1B2|B3GAT2|BDNF|CA2|CES2|CHST11|CHST3|CHST6|CHST8|CP|CRYZ|CYP11A1|CYP1A1|CYP1A2|CYP2C9|CYP4X1|CYP7A1|DIO2|DIP2A|DLG4|DNMT1|DNMT3A|DNMT3B|DPP6|EN2|EPHX1|FLT1|GFAP|GPX2|GRIN2B|GSTA2|GSTM1|GSTO1|GSTP1|HFE|ITGB3|JAM3|KIFC1|LAMC3|LRP1|LRRN3|MEF2C|MET|MTR|MTTP|NOS1|NOS2|NOS3|NOTCH2|NQO1|NRXN1|NRXN2|NSUN2|PSG6|PTCHD1|PTGR1|SLC1A1|SLC2A3|SYN1|TET1|TFRC|TJP1|TJP2|TP53I3|TXNRD1|UNC80
Mercury	D008628	ABCB1|ABCG2|ACHE|AHR|AKR7A3|ALAD|ALDH5A1|ALDH6A1|AQP4|AQP9|BDNF|CA2|CFTR|CHAT|COMT|CP|CRYZ|CYP1A1|CYP1A2|CYP2U1|CYP7A1|DRD4|GNGT1|GSTA2|GSTM1|GSTP1|MTR|NOS2|NQO1|RELN|SLC3A2|SLC6A4|TJP1|TXNRD1|TXNRD2
Cadmium	D002104	ABCB1|ABCG2|ACHE|ADH5|ADH6|AHR|AKR1B10|AKR1C2|AKR1C3|ALAD|ALDH1A1|ALDH2|ALDH3A2|ALDH4A1|ALDH6A1|BDNF|CBS|CFTR|CHAT|CIRBP|COMT|CYP11A1|CYP19A1|CYP1A1|DNMT1|DNMT3B|DRD4|EPHX1|FLT1|GPX2|GPX5|GSTA3|GSTM1|GSTO1|GSTP1|IL1RL1|ITPR3|MET|MIF|MTTP|NOS1|NOS2|NOS3|NOTCH2|NOTCH3|NQO1|NTSR1|PTGS1|RELN|SCNN1A|SLC16A1|SLC25A20|SLC2A3|SLC3A2|TET1|TFRC|TJP1|TXNRD1
Zinc	D015032	ABCB1|ACHE|ADA|ADH4|ADH5|ADH6|ADRB2|AHR|AKR1A1|AKR1C3|ALAD|ALDH1A1|ALDH2|ALDH3B1|ALDH6A1|ALOX15|ANKRD11|AOC2|AOX1|APC|AQP4|ATP1A3|BDNF|CA2|CFTR|CHD2|CHST10|CIC|CP|CRYZ|CYP1A2|CYP24A1|CYP27A1|CYP27C1|CYP2D6|CYP2F1|CYP2R1|CYP3A5|CYP4B1|CYP7A1|DHRS2|DNMT3A|DRD4|EPHX1|FLT1|FMO2|FMO4|GRIN2B|HFE|HGF|INSR|JARID2|LRRN3|MTTP|NOS2|NOS3|NOTCH1|NQO1|NQO2|PSG1|PTGES|PTGES2|PTGS1|SCN1A|SHANK3|SLC1A1|SLC1A4|SLC25A25|SLC2A3|SLC3A2|SLC6A4|SLCO1C1|STS|SULF1|TFRC|TJP1|TPST2|TXNRD1|UGT1A1

Arsenic	D001151	ABCB1|ABCC3|ACHE|AHR|AKR1B10|AKR1C2|AKR1C3|ALAD|ALDH3B2|AQP9|BCHE|BDNF|CBS|CFTR|CNTNAP2|CYP11A1|CYP19A1|CYP1A1|CYP26B1|CYP2U1|DLG4|DNMT1|DNMT3A|DNMT3B|FMO2|FOXP1|GPX2|GSTM1|GSTO1|GSTP1|HGF|HSD3B1|IL1RL1|INSR|MEF2C|MTHFR|MTR|NOS2|NOS3|NOTCH1|NQO1|SLC22A5|SLC3A2|SLCO2A1|SOX9|SULT2B1|TXNRD1|UGT1A1|UGT1A6|VCL|VWF
Glyphosate	D000097797	ABCA2|ABCB1|ABCG2|ACHE|AHR|ALDH1A3|ALDH3B1|ALDH4A1|AOC1|AVPR1A|B3GAT2|BCHE|BDNF|CBS|CGN|CHAT|CHD8|CIRBP|CRYZ|CYP11A1|CYP19A1|CYP1A1|CYP2C9|CYP2S1|DHRS1|DIO2|DIO3|DNAH11|DNAH7|DNMT1|DNMT3A|DNMT3B|DPP10|DPP6|DRD4|EPHX2|FLT1|FMO1|FMO3|FOLR2|GFAP|GPX2|GSTA2|GSTM1|GSTO1|GTF2I|HGF|HSD3B1|IL1RL1|ITPR3|LAMC3|LOXL2|MET|MIF|MTTP|NOS2|NOTCH1|NOTCH4|NQO1|NRXN1|NRXN2|ODAD2|OXTR|PAH|PARD3|PTGS1|RARB|RELN|SCNN1A|SHANK3|SLC16A2|SLC22A5|SLC6A4|SLCO1C1|SOX5|SOX9|SULF1|SULF2|TET3|TJP1|TP53I3|TPMT|UGT1A10|USH2A
perfluorooctane sulfonic acid	C076994	ABCA2|ABCC3|ABCG2|ACHE|ADH1A|ADH4|AKR1A1|ALAD|ALDH1A1|ALDH1L1|ALDH2|ALDH3A2|ALDH6A1|AOX1|AQP9|ARSG|ARSI|ARSK|AVPR1A|BDNF|CA2|CBR1|CBR3|CBS|CES2|CFTR|CGN|CGNL1|CIC|COMT|CP|CRYZ|CYP11A1|CYP11B2|CYP19A1|CYP1A1|CYP1A2|CYP27A1|CYP2B6|CYP2C19|CYP2C8|CYP2D6|CYP2J2|CYP39A1|CYP3A7|CYP4A11|CYP4A22|CYP7A1|DHCR24|DHRS1|DNMT1|DNMT3A|DNMT3B|EPHX1|EPHX2|FLT1|GAL3ST1|GALNS|GFAP|GPX2|GRIN2B|GSTA2|GSTA3|GSTM1|GSTM5|GSTO1|GSTP1|GSTT2|HEY1|HGF|HSD17B1|HSD3B1|HTRA4|INSR|IQGAP3|LOXL4|LRP1|MEF2C|MET|MIF|MPDZ|MTHFR|MTTP|NOS2|NOS3|NOTCH1|NQO1|NQO2|NRXN2|PON2|PON3|PROK1|PTGES|PTGIS|PTGR1|RELN|ROGDI|RSPH4A|SCNN1A|SFSWAP|SLC16A1|SLC16A2|SLC19A1|SLC1A1|SLC1A4|SLC22A5|SLC25A20|SLC25A25|SLC3A2|SLCO1C1|SLCO2A1|SOX9|SULF2|SULT1B1|SULT1C2|TET3|TFRC|TJP1|TPMT|TXNRD1|UGT1A1|UGT2A3|UGT2B10|UGT2B7|UPP2
perfluorohexanesulfonic acid	C471071	ABCA2|ABCG2|ADA|AKR1B10|ALDH3A2|ALDH4A1|ALOX12|ALOX12B|AOC2|AOX1|AVPR1A|B3GAT2|BDNF|CBR1|CHD2|CIRBP|CYP11A1|CYP19A1|CYP24A1|CYP27A1|CYP2B6|CYP2C19|CYP2D6|CYP2S1|CYP2U1|CYP3A5|CYP3A7|CYP7A1|DIO2|DIO3|DNAAF2|DNAH11|EBI3|FLT1|FMO1|FMO2|GFAP|GSTM1|GSTP1|GSTT2|GTF2I|HEY1|HFE|HGF|HSD17B1|IFT74|ITPR3|JAM2|JAM3|JARID2|KIFC1|LOXL1|MAGEA8|MTTP|NOS1|NOS2|NOS3|NQO1|NSUN2|ODAD2|OXTR|PAPPA2|PON2|PON3|PRKCZ|PTGR1|RAI1|SCN1A|SCNN1A|SCNN1G|SLC12A7|SLC16A2|SLC19A1|SLC22A5|SLC25A20|SLC3A2|SLCO2A1|SOX5|SULT1C3|SUOX|TPMT|UGT1A1
Polychlorinated Biphenyls	D011078	ABCC3|ACHE|AHR|AKR1C2|ALDH1A1|ALDH1A3|ALDH3A1|ALDH6A1|AOC1|AOX1|B3GAT2|BDNF|CA2|CES3|CNTNAP2|COMT|CP|CYP11A1|CYP11B2|CYP19A1|CYP1A1|CYP1A2|CYP21A2|CYP27B1|CYP2B6|CYP2R1|CYP7A1|DLG4|DNMT1|DNMT3A|DNMT3B|DPP10|EPHX1|FLT1|FMO1|FMO3|FOXP1|FOXP2|GALNS|GPX2|GPX6|GRIN2B|GSTA2|GSTA3|GSTM1|GSTP1|GSTT2|HSD17B1|HSD3B1|IL1RAPL1|IQGAP3|ITPR3|KIF17|KIFC1|LRRN3|LRRTM3|MEF2C|MET|MTHFR|MTTP|NOS2|NOTCH1|NQO1|NTSR1|PCDH9|PON3|RAI1|RYR2|SCN1A|SCNN1A|SLC25A20|SLC3A2|SLC6A4|SULT1B1|SULT2B1|TJP1|UGT1A1|UGT1A6|UNC80

2,4,5,2',4',5'-hexachlorobiphenyl	C014024	ABCA2|ABCC3|AHR|AKR1C2|AKR1C3|ALDH1A1|ALDH3A2|ALDH3B2|AOC2|AOX1|B3GAT2|CA2|CES2|CES3|CNTNAP2|COMT|CYP11A1|CYP11B2|CYP19A1|CYP1A1|CYP1A2|CYP27B1|CYP2R1|CYP2U1|CYP39A1|CYP7A1|DIO2|DLG4|DNMT3B|DPP10|EPHX1|EPHX2|FLT1|FMO1|FMO2|FMO3|FOXP1|FOXP2|GALNS|GPX2|GPX6|GRIN2B|GSTA2|GSTM1|GSTM5|GSTT2|HSD17B1|HSD3B1|IL1RAPL1|INSR|IQGAP3|ITPR3|KIF17|KIFC1|LRRN3|LRRTM3|MET|MTHFR|MTTP|NOS2|NOTCH1|NQO1|NQO2|NTSR1|PCDH9|PTGR1|RAI1|RARB|SCN1A|SCNN1A|SLC1A4|SLC25A20|SLC6A4|SULT1B1|SULT2B1|TJP1|TPMT|TXNRD1|UGT1A1|UNC80

Supplementary Table S15. Pollutant-Disorder Gene Sets for Intellectual Disability (MESH: D008607)

Chemical Name	Chemical ID	Gene Sets
Total Pollutants	-	ADK|ADRA2B|ALDH5A1|AP4E1|APC|ARL14EP|BBS7|BDNF|CA8|CACNA1G|CALCA|CAPN10|CASP2|CCBE1|CCNA2|CHD8|CHL1|CIC|COL18A1|COQ5|DCC|DISC1|DNMT3A|DOCK8|DYNC1H1|EEF1B2|ENTPD1|FASN|FGFR2|FMR1|FOLR1|FRY|GAMT|GNAS|GON4L|GRIN2B|H3-4|H4C3|HDAC4|HEXA|HYCC1|INPP4A|KCNA2|KDM5A|KDM5C|KDM6B|KIF7|L2HGDH|LAMA1|LARP7|LETM1|MCC|MECP2|MED13L|MEF2C|METTL23|MFSD2A|NAA15|NAGLU|NF1|NRXN1|NSD1|NTF4|PARP1|PAX6|PDHX|PEX6|PHIP|POLR3B|PRKCG|PRKRA|PTCHD1|PTEN|RABL6|RAI1|RALGDS|RGS7|SC5D|SCAPER|SCN1A|SCN8A|SETBP1|SHANK2|SHANK3|SLC2A1|SLC31A1|SLC4A10|SRGAP3|SYNGAP1|TAF2|TBCD|TH|TMCO1|TSEN54|WDR45B|WDR62|YY1|ZBTB40
Air Pollutants	D000393	ALDH5A1|BDNF|CACNA1G|CALCA|CCNA2|CHL1|CIC|DYNC1H1|ENTPD1|FASN|GNAS|GON4L|INPP4A|KCNA2|KDM6B|LAMA1|MCC|MECP2|MEF2C|MFSD2A|NAGLU|NRXN1|PARP1|PDHX|PRKCG|RALGDS|SC5D|SCAPER|SHANK3|SLC2A1|SRGAP3|TH|ZBTB40
Particulate Matter	D052638	ADRA2B|APC|BDNF|CALCA|CCBE1|CCNA2|CHL1|COL18A1|DNMT3A|FASN|FGFR2|LAMA1|MECP2|MEF2C|NAGLU|NRXN1|PARP1|PTEN|SHANK3|SLC2A1
Lead	D007854	ADK|ALDH5A1|AP4E1|BDNF|CA8|CCNA2|CHL1|DCC|DNMT3A|EEF1B2|ENTPD1|FASN|GAMT|GNAS|GRIN2B|HDAC4|HYCC1|INPP4A|MECP2|MEF2C|NAA15|NRXN1|NSD1|PARP1|PAX6|PDHX|PEX6|POLR3B|PRKCG|PTCHD1|PTEN|RABL6|RALGDS|RGS7|SETBP1|SHANK2|SLC2A1|SLC31A1|TAF2|TH|WDR45B|WDR62|YY1|ZBTB40
Zinc	D015032	APC|ARL14EP|BDNF|CALCA|CCNA2|CIC|COL18A1|DNMT3A|DYNC1H1|FASN|FMR1|GRIN2B|HDAC4|INPP4A|KDM6B|L2HGDH|MED13L|NF1|PARP1|PAX6|PRKCG|PTEN|RALGDS|SCN1A|SHANK2|SHANK3|SLC2A1|SLC31A1|TH
Glyphosate	D000097797	AP4E1|BDNF|CALCA|CASP2|CCNA2|CHD8|DNMT3A|DYNC1H1|FASN|FOLR1|FRY|GAMT|HDAC4|KCNA2|KDM5A|MED13L|MFSD2A|NRXN1|NSD1|NTF4|PARP1|PAX6|PRKRA|SC5D|SCAPER|SHANK3|SLC2A1|SYNGAP1|TH

perfluorooctane sulfonic acid	C076994	BDNF|CAPN10|CIC|COQ5|DNMT3A|DOCK8|DYNC1H1|EEF1B2|FASN|FGFR2|GNAS|GRIN2B|HEXA|HYCC1|KDM5C|KIF7|LARP7|LETM1|MCC|MECP2|MEF2C|METTL23|MFSD2A|PARP1|POLR3B|PRKCG|SC5D|SCN8A|SETBP1|SLC2A1|SLC31A1|SRGAP3|TAF2|TBCD|TH|TMCO1|TSEN54
perfluorohexanesulfonic acid	C471071	BBS7|BDNF|CAPN10|FASN|FGFR2|GNAS|GON4L|H3-4|H4C3|HDAC4|HEXA|LAMA1|LETM1|PRKRA|PTEN|RABL6|RAI1|RALGDS|SCN1A|SETBP1|TSEN54
Polychlorinated Biphenyls	D011078	AP4E1|BDNF|CCNA2|COL18A1|DISC1|DNMT3A|FASN|FGFR2|FOLR1|FRY|GNAS|GRIN2B|INPP4A|KDM6B|LARP7|MEF2C|PARP1|PHIP|RAI1|SCN1A|SETBP1|SHANK2|SLC2A1|SLC4A10|TH|WDR45B
2,4,5,2',4',5'-hexachlorobiphenyl	C014024	AP4E1|CCNA2|DISC1|FASN|FOLR1|FRY|GNAS|GRIN2B|INPP4A|KDM6B|LARP7|MFSD2A|PARP1|PHIP|PTEN|RAI1|SC5D|SCN1A|SETBP1|SHANK2|SLC2A1|SLC4A10|TH|WDR45B

S1.8 Characterizing Developmental Transcriptome Signatures of Pollutant-Disorder Gene Sets

S1.8.1 BrainSpan Developmental Transcriptome Dataset

The Allen Institute BrainSpan Atlas provides RNA-seq data for 16 human brain regions, including the cerebellar cortex, thalamus, striatum, amygdala, hippocampus, and 11 areas of the neocortex. [7] The dataset, derived from 57 post-mortem brains of clinically unremarkable donors (ages 8 post-conceptual weeks to 40 years), has a missing data rate of approximately 52%.[8] To address this, Pei et al. developed a tensor-based approach to impute missing gene expression data, completing the temporal-spatial transcriptomic grid.[8] Excluding individuals or brain regions with more than 50% missing data, the resulting tensor includes 18,911 genes across 35 individuals and 16 regions, with 478 measured and 82 imputed transcriptomes. The imputation method was validated using leave-one-out (LOO) cross-validation. Human developmental periods were categorized as early prenatal (12 pcw), intermediate prenatal (13–24 pcw), late prenatal (37 pcw), early postnatal (4 months–4 years), intermediate postnatal (8–13 years), and late postnatal (18–40 years). The imputed BrainSpan dataset was downloaded from GitHub (accessed on 11/20/2023, https://github.com/bsml320/BrainSpan).

S1.8.2 Gene Expression Analysis

To evaluate the influence of age on gene expression, we fit a multivariable linear model for each gene: expression ~ age + sex + brain region. This model was implemented using the *lmFit()* and *eBayes()* functions from the **limma** R package (v3.62.1).[9] This approach generated t-statistics (one for each temporal dimension) along with two-sided P-values. Genes were ranked based on their t-statistics, and the top 5% of genes with the highest expression levels and the bottom 5% with the lowest expression levels were selected to construct temporal-specific groups of significantly highly and lowly expressed genes. Fisher's exact test was then used to assess the association between the pollutant-disorder gene sets and these temporal-specific expression groups. Odds ratios (ORs) and adjusted P-values (using Bonferroni correction for 25 multiple time comparisons) were calculated for each test. An OR > 1 indicates a positive correlation, an OR < 1 indicates a negative correlation, and adjusted P-values < 0.05 were considered statistically significant.

S1.8.3 Principal Component Analysis

The gene expression matrix was filtered based on the pollutant-disorder gene sets, narrowing the analysis to genes mapped to the BrainSpan database. Principal component analysis (PCA) was then performed to extract the expression features of pollutant-disorder genes across 560 samples. A scatter plot was constructed using the first and second principal components (PC1 and PC2), with each sample labeled by its corresponding time point, to assess the relationship between the pollutant-disorder gene sets and brain development stages. The proportion of variance explained by the top 10 principal components was calculated, and a cumulative explained variance plot for the first 10 components was generated. The same procedure was applied to the gene clusters identified by the time-series analysis.

S1.8.4 Trajectory Analysis

The filtered expression data were first standardized using the *standardize()* function from the **Mfuzz** package (version 2.66.0)[10] to normalize the data by centering and scaling the expression levels across genes. Genes were then clustered based on their expression profiles over time using the *mfuzz()* function, with the number of clusters set to 3 based on biological relevance. Each gene was assigned to one of the three clusters according to its expression trend, reflecting patterns of gene expression across the analyzed time points. After clustering, the biological functions of each gene cluster were assessed through Gene Ontology (GO) annotation to characterize the biological processes associated with each cluster. This analysis provided insights into key biological processes related to the expression patterns of pollutant-disorder genes and their potential association with different neurodevelopmental stages.

S2. Supplementary Results

S2.1 Testing Chemical Enrichment in Neurodevelopmental Disorders

We generated 2×2 contingency tables for genes associated with each chemical and genes associated with each neurodevelopmental disorder. For each contingency table, we calculated the expected frequencies and selected the appropriate analysis based on the size of the expected frequencies in the cells, as follows:

- When the expected frequency in each cell is greater than or equal to 5, a standard Chi-square test was used to assess the independence of chemical-gene enrichment.
- When the expected frequency in each cell is less than 5 but greater than 1, the Chi-square test with Yates' continuity correction was applied.
- Fisher's exact test was applied if the expected frequency in any cell was less than or equal to 1.

2×2 Contingency Tables	Not Disease Gene	Disease Gene
Not Chemical Gene	A	B
Chemical Gene	C	D

For each neurodevelopmental disorder, 69 multiple comparisons were performed, and Bonferroni correction was applied to account for multiple testing. A corrected P-value less than 0.05 was considered statistically significant. P-values equal to 1 were not presented in the results.

S2.1.1 Enrichment Analysis for Attention Deficit Disorder with Hyperactivity (MESH: D001289)

Supplementary Table S16. Results of Enrichment Analysis for Attention Deficit Disorder with Hyperactivity

Gene Sets	MeSH® ID	Statistical Method	Crude P-value	Corrected P-value
Air Pollutants	D000393	Fisher's Exact Test	0.045	-
Particulate Matter	D052638	Fisher's Exact Test	0.217	-
Nitrogen Oxides	D009589	Fisher's Exact Test	0.039	-
Sulfur Oxides	D013461	Fisher's Exact Test	0.138	-
Carbon Monoxide	D002248	Fisher's Exact Test	-	-
Ozone	D010126	Fisher's Exact Test	-	-
Polycyclic Aromatic Hydrocarbons	D011084	Fisher's Exact Test	0.002	0.117
Phenanthrene	C031181	Fisher's Exact Test	0.094	-

Pyrene	C030984	Fisher's Exact Test	0.094	-
Lead	D007854	Fisher's Exact Test	0.239	-
Mercury	D008628	Fisher's Exact Test	0.038	-
Cadmium	D002104	Fisher's Exact Test	0.183	-
Manganese	D008345	Fisher's Exact Test	0.003	0.188
Antimony	D000965	Fisher's Exact Test	0.006	0.387
Barium	D001464	Fisher's Exact Test	<0.001	0.001 [a]
Cobalt	D003035	Fisher's Exact Test	-	-
Nickel	D009532	Fisher's Exact Test	0.071	-
Zinc	D015032	Fisher's Exact Test	<0.001	0.001 [a]
Copper	D003300	Fisher's Exact Test	-	-
Iron	D007501	Fisher's Exact Test	0.019	-
Aluminum	D000535	Fisher's Exact Test	-	-
Lithium	D008094	Fisher's Exact Test	0.122	-
Magnesium	D008274	Fisher's Exact Test	-	-
Arsenic	D001151	Fisher's Exact Test	0.395	-
Pesticides	D010575	Fisher's Exact Test	0.001	0.094
Fungicides, Industrial	D005659	Fisher's Exact Test	-	-
Herbicides	D006540	Fisher's Exact Test	0.151	-
Insecticides	D007306	Fisher's Exact Test	-	-
Organophosphates	D010755	Fisher's Exact Test	0.075	-
Hydrocarbons, Chlorinated	D006843	Fisher's Exact Test	<0.001	<0.001 [a]
Carbamates	D002219	Fisher's Exact Test	<0.001	<0.001 [a]
Pyrethrins	D011722	Fisher's Exact Test	<0.001	<0.001 [a]
Hexachlorobenzene	D006581	Fisher's Exact Test	0.100	-
Glyphosate	D000097797	Fisher's Exact Test	0.162	-
DEET	D003671	Fisher's Exact Test	0.003	0.227
DDT	D003634	Fisher's Exact Test	-	-

Dichlorodiphenyl Dichloroethylene	D003633	Fisher's Exact Test	0.147	-
Parathion	D010278	Fisher's Exact Test	0.124	-
Methyl parathion	D008743	Fisher's Exact Test	-	-
Dieldrin	D004026	Fisher's Exact Test	<0.001	<0.001 [a]
Aldrin	D000452	Fisher's Exact Test	0.102	-
Triclosan	D014260	Fisher's Exact Test	0.076	-
Fluorocarbons	D005466	Fisher's Exact Test	0.164	-
perfluorooctanoic acid	C023036	Fisher's Exact Test	0.166	-
perfluorooctane sulfonic acid	C076994	Fisher's Exact Test	0.006	0.407
perfluorodecanoic acid	C036567	Fisher's Exact Test	0.104	-
perfluoro-n-nonanoic acid	C101816	Fisher's Exact Test	0.077	-
perfluorohexanesulfonic acid	C471071	Fisher's Exact Test	0.034	-
Polychlorinated Biphenyls	D011078	Fisher's Exact Test	<0.001	0.020 [a]
2,3',4,4',5-pentachlorobiphenyl	C070055	Fisher's Exact Test	0.461	-
2,2',3',4,4',5-hexachlorobiphenyl	C029790	Fisher's Exact Test	0.066	-
2,4,5,2',4',5'-hexachlorobiphenyl	C014024	Fisher's Exact Test	<0.001	0.003 [a]
2,2',3,3',4,4',5-heptachlorobiphenyl	C541131	Fisher's Exact Test	-	-
Halogenated Diphenyl Ethers	D055768	Fisher's Exact Test	<0.001	<0.001 [a]
Dibutyl Phthalate	D003993	Fisher's Exact Test	0.001	0.050
Diethylhexyl Phthalate	D004051	Fisher's Exact Test	-	-
diethyl phthalate	C007379	Fisher's Exact Test	-	-
Plasticizers	D010968	Fisher's Exact Test	-	-
bisphenol A	C006780	Fisher's Exact Test	-	-
bisphenol B	C492482	Fisher's Exact Test	0.596	-
bisphenol S	C543008	Fisher's Exact Test	-	-
oxybenzone	C005290	Fisher's Exact Test	-	-
Plastics	D010969	Fisher's Exact Test	-	-
Microplastics	D000080545	Fisher's Exact Test	-	-

Chloroform	D002725	Fisher's Exact Test	-	-
Methylene chloride	D008752	Fisher's Exact Test	-	-
Tetrachloroethylene	D013750	Fisher's Exact Test	0.063	-
Trichloroethylene	D014241	Fisher's Exact Test	0.002	0.139
Carbon Tetrachloride	D002251	Fisher's Exact Test	0.102	-

Note: [a] Statistically significant.

S2.1.2 Enrichment Analysis for Autism Spectrum Disorder (MESH: D000067877)

Supplementary Table S17. Results of Enrichment Analysis for Autism Spectrum Disorder

Gene Sets	MeSH® ID	Statistical Method	Crude P-value	Corrected P-value
Air Pollutants	D000393	Standard Chi-Square	<0.001	<0.001 [a]
Particulate Matter	D052638	Standard Chi-Square	<0.001	<0.001 [a]
Nitrogen Oxides	D009589	Fisher's Exact Test	0.004	0.287
Sulfur Oxides	D013461	Fisher's Exact Test	<0.001	<0.001 [a]
Carbon Monoxide	D002248	Fisher's Exact Test	<0.001	0.003 [a]
Ozone	D010126	Standard Chi-Square	<0.001	<0.001 [a]
Polycyclic Aromatic Hydrocarbons	D011084	Fisher's Exact Test	<0.001	<0.001 [a]
Phenanthrene	C031181	Fisher's Exact Test	<0.001	<0.001 [a]
Pyrene	C030984	Fisher's Exact Test	<0.001	<0.001 [a]
Lead	D007854	Standard Chi-Square	<0.001	<0.001 [a]
Mercury	D008628	Standard Chi-Square	<0.001	<0.001 [a]
Cadmium	D002104	Standard Chi-Square	<0.001	<0.001 [a]
Manganese	D008345	Fisher's Exact Test	<0.001	<0.001 [a]
Antimony	D000965	Fisher's Exact Test	<0.001	<0.001 [a]
Barium	D001464	Fisher's Exact Test	0.029	-
Cobalt	D003035	Fisher's Exact Test	<0.001	<0.001 [a]
Nickel	D009532	Standard Chi-Square	<0.001	<0.001 [a]
Zinc	D015032	Standard Chi-Square	<0.001	<0.001 [a]
Copper	D003300	Fisher's Exact Test	<0.001	<0.001 [a]
Iron	D007501	Fisher's Exact Test	<0.001	<0.001 [a]
Aluminum	D000535	Fisher's Exact Test	<0.001	<0.001 [a]
Lithium	D008094	Fisher's Exact Test	<0.001	<0.001 [a]
Magnesium	D008274	Fisher's Exact Test	<0.001	<0.001 [a]
Arsenic	D001151	Standard Chi-Square	<0.001	<0.001 [a]

Pesticides	D010575	Standard Chi-Square	<0.001	<0.001 [a]
Fungicides, Industrial	D005659	Fisher's Exact Test	<0.001	0.006 [a]
Herbicides	D006540	Fisher's Exact Test	<0.001	<0.001 [a]
Insecticides	D007306	Fisher's Exact Test	<0.001	<0.001 [a]
Organophosphates	D010755	Fisher's Exact Test	<0.001	<0.001 [a]
Hydrocarbons, Chlorinated	D006843	Standard Chi-Square	<0.001	<0.001 [a]
Carbamates	D002219	Standard Chi-Square	<0.001	<0.001 [a]
Pyrethrins	D011722	Standard Chi-Square	<0.001	<0.001 [a]
Hexachlorobenzene	D006581	Fisher's Exact Test	<0.001	<0.001 [a]
Glyphosate	D000097797	Standard Chi-Square	<0.001	<0.001 [a]
DEET	D003671	Standard Chi-Square	<0.001	<0.001 [a]
DDT	D003634	Fisher's Exact Test	<0.001	<0.001 [a]
Dichlorodiphenyl Dichloroethylene	D003633	Standard Chi-Square	<0.001	<0.001 [a]
Parathion	D010278	Standard Chi-Square	<0.001	<0.001 [a]
Methyl parathion	D008743	Fisher's Exact Test	<0.001	<0.001 [a]
Dieldrin	D004026	Standard Chi-Square	<0.001	<0.001 [a]
Aldrin	D000452	Fisher's Exact Test	<0.001	<0.001 [a]
Triclosan	D014260	Standard Chi-Square	<0.001	<0.001 [a]
Fluorocarbons	D005466	Standard Chi-Square	<0.001	<0.001 [a]
perfluorooctanoic acid	C023036	Fisher's Exact Test	<0.001	<0.001 [a]
perfluorooctane sulfonic acid	C076994	Standard Chi-Square	<0.001	<0.001 [a]
perfluorodecanoic acid	C036567	Fisher's Exact Test	<0.001	<0.001 [a]
perfluoro-n-nonanoic acid	C101816	Standard Chi-Square	<0.001	<0.001 [a]
perfluorohexanesulfonic acid	C471071	Standard Chi-Square	<0.001	<0.001 [a]
Polychlorinated Biphenyls	D011078	Standard Chi-Square	<0.001	<0.001 [a]
2,3',4,4',5-pentachlorobiphenyl	C070055	Standard Chi-Square	<0.001	<0.001 [a]
2,2',3',4,4',5-hexachlorobiphenyl	C029790	Standard Chi-Square	<0.001	<0.001 [a]
2,4,5,2',4',5'-hexachlorobiphenyl	C014024	Standard Chi-Square	<0.001	<0.001 [a]

2,2',3,3',4,4',5-heptachlorobiphenyl	C541131	Fisher's Exact Test	0.002	<0.001 [a]
Halogenated Diphenyl Ethers	D055768	Standard Chi-Square	<0.001	<0.001 [a]
Dibutyl Phthalate	D003993	Standard Chi-Square	<0.001	<0.001 [a]
Diethylhexyl Phthalate	D004051	Fisher's Exact Test	<0.001	<0.001 [a]
diethyl phthalate	C007379	Fisher's Exact Test	<0.001	<0.001 [a]
Plasticizers	D010968	Fisher's Exact Test	0.009	0.617
bisphenol A	C006780	Fisher's Exact Test	<0.001	<0.001 [a]
bisphenol B	C492482	Standard Chi-Square	<0.001	<0.001 [a]
bisphenol S	C543008	Fisher's Exact Test	0.010	0.669
oxybenzone	C005290	Fisher's Exact Test	<0.001	<0.001 [a]
Plastics	D010969	Fisher's Exact Test	<0.001	<0.001 [a]
Microplastics	D000080545	Fisher's Exact Test	0.002	0.164
Chloroform	D002725	Fisher's Exact Test	<0.001	<0.001 [a]
Methylene chloride	D008752	Fisher's Exact Test	0.018	-
Tetrachloroethylene	D013750	Standard Chi-Square	<0.001	<0.001 [a]
Trichloroethylene	D014241	Standard Chi-Square	<0.001	<0.001 [a]
Carbon Tetrachloride	D002251	Standard Chi-Square	<0.001	<0.001 [a]

Note: [a] Statistically significant.

S2.1.3 Enrichment Analysis for Autistic Disorder (MESH: D001321)

Supplementary Table S18. Results of Enrichment Analysis for Autistic Disorder

Gene Sets	MeSH® ID	Statistical Method	Crude P-value	Corrected P-value
Air Pollutants	D000393	Standard Chi-Square	<0.001	<0.001 [a]
Particulate Matter	D052638	Standard Chi-Square	<0.001	<0.001 [a]
Nitrogen Oxides	D009589	Fisher's Exact Test	<0.001	<0.001 [a]
Sulfur Oxides	D013461	Fisher's Exact Test	<0.001	<0.001 [a]
Carbon Monoxide	D002248	Fisher's Exact Test	<0.001	<0.001 [a]
Ozone	D010126	Standard Chi-Square	<0.001	<0.001 [a]
Polycyclic Aromatic Hydrocarbons	D011084	Fisher's Exact Test	<0.001	<0.001 [a]
Phenanthrene	C031181	Fisher's Exact Test	<0.001	<0.001 [a]
Pyrene	C030984	Fisher's Exact Test	<0.001	<0.001 [a]
Lead	D007854	Standard Chi-Square	<0.001	<0.001 [a]
Mercury	D008628	Fisher's Exact Test	<0.001	<0.001 [a]
Cadmium	D002104	Standard Chi-Square	<0.001	<0.001 [a]
Manganese	D008345	Fisher's Exact Test	<0.001	<0.001 [a]
Antimony	D000965	Fisher's Exact Test	<0.001	<0.001 [a]
Barium	D001464	Fisher's Exact Test	<0.001	<0.001 [a]
Cobalt	D003035	Fisher's Exact Test	<0.001	<0.001 [a]
Nickel	D009532	Standard Chi-Square	<0.001	<0.001 [a]
Zinc	D015032	Standard Chi-Square	<0.001	<0.001 [a]
Copper	D003300	Fisher's Exact Test	<0.001	<0.001 [a]
Iron	D007501	Fisher's Exact Test	<0.001	<0.001 [a]
Aluminum	D000535	Fisher's Exact Test	<0.001	<0.001 [a]
Lithium	D008094	Fisher's Exact Test	<0.001	<0.001 [a]
Magnesium	D008274	Fisher's Exact Test	<0.001	<0.001 [a]
Arsenic	D001151	Standard Chi-Square	<0.001	<0.001 [a]

Pesticides	D010575	Standard Chi-Square	<0.001	<0.001 [a]
Fungicides, Industrial	D005659	Fisher's Exact Test	<0.001	<0.001 [a]
Herbicides	D006540	Fisher's Exact Test	<0.001	<0.001 [a]
Insecticides	D007306	Fisher's Exact Test	<0.001	<0.001 [a]
Organophosphates	D010755	Fisher's Exact Test	<0.001	<0.001 [a]
Hydrocarbons, Chlorinated	D006843	Standard Chi-Square	<0.001	<0.001 [a]
Carbamates	D002219	Standard Chi-Square	<0.001	<0.001 [a]
Pyrethrins	D011722	Standard Chi-Square	<0.001	<0.001 [a]
Hexachlorobenzene	D006581	Fisher's Exact Test	<0.001	<0.001 [a]
Glyphosate	D000097797	Standard Chi-Square	<0.001	<0.001 [a]
DEET	D003671	Standard Chi-Square	<0.001	<0.001 [a]
DDT	D003634	Fisher's Exact Test	<0.001	<0.001 [a]
Dichlorodiphenyl Dichloroethylene	D003633	Standard Chi-Square	<0.001	<0.001 [a]
Parathion	D010278	Standard Chi-Square	<0.001	<0.001 [a]
Methyl parathion	D008743	Fisher's Exact Test	<0.001	<0.001 [a]
Dieldrin	D004026	Standard Chi-Square	<0.001	<0.001 [a]
Aldrin	D000452	Fisher's Exact Test	0.037	-
Triclosan	D014260	Standard Chi-Square	<0.001	<0.001 [a]
Fluorocarbons	D005466	Standard Chi-Square	<0.001	<0.001 [a]
perfluorooctanoic acid	C023036	Fisher's Exact Test	<0.001	<0.001 [a]
perfluorooctane sulfonic acid	C076994	Standard Chi-Square	<0.001	<0.001 [a]
perfluorodecanoic acid	C036567	Fisher's Exact Test	<0.001	<0.001 [a]
perfluoro-n-nonanoic acid	C101816	Standard Chi-Square	<0.001	<0.001 [a]
perfluorohexanesulfonic acid	C471071	Standard Chi-Square	<0.001	<0.001 [a]
Polychlorinated Biphenyls	D011078	Standard Chi-Square	<0.001	<0.001 [a]
2,3',4,4',5-pentachlorobiphenyl	C070055	Standard Chi-Square	<0.001	0.001
2,2',3',4,4',5-hexachlorobiphenyl	C029790	Standard Chi-Square	<0.001	<0.001 [a]
2,4,5,2',4',5'-hexachlorobiphenyl	C014024	Standard Chi-Square	<0.001	<0.001 [a]

2,2',3,3',4,4',5-heptachlorobiphenyl	C541131	Fisher's Exact Test	0.001	0.058
Halogenated Diphenyl Ethers	D055768	Standard Chi-Square	<0.001	<0.001 [a]
Dibutyl Phthalate	D003993	Standard Chi-Square	<0.001	<0.001 [a]
Diethylhexyl Phthalate	D004051	Fisher's Exact Test	<0.001	<0.001 [a]
diethyl phthalate	C007379	Fisher's Exact Test	<0.001	<0.001 [a]
Plasticizers	D010968	Fisher's Exact Test	<0.001	<0.001 [a]
bisphenol A	C006780	Fisher's Exact Test	<0.001	<0.001 [a]
bisphenol B	C492482	Standard Chi-Square	<0.001	<0.001 [a]
bisphenol S	C543008	Fisher's Exact Test	<0.001	0.011 [a]
oxybenzone	C005290	Fisher's Exact Test	<0.001	<0.001 [a]
Plastics	D010969	Fisher's Exact Test	<0.001	<0.001 [a]
Microplastics	D000080545	Fisher's Exact Test	0.016	-
Chloroform	D002725	Fisher's Exact Test	<0.001	<0.001 [a]
Methylene chloride	D008752	Fisher's Exact Test	<0.001	<0.001 [a]
Tetrachloroethylene	D013750	Fisher's Exact Test	<0.001	0.026 [a]
Trichloroethylene	D014241	Standard Chi-Square	<0.001	<0.001 [a]
Carbon Tetrachloride	D002251	Standard Chi-Square	<0.001	<0.001 [a]

Note: [a] Statistically significant.

S2.1.4 Enrichment Analysis for Developmental Disabilities (MESH: D002658)

Supplementary Table S19. Results of Enrichment Analysis for Developmental Disabilities

Gene Sets	MeSH® ID	Statistical Method	Crude P-value	Corrected P-value
Air Pollutants	D000393	Fisher's Exact Test	0.016	-
Particulate Matter	D052638	Fisher's Exact Test	0.001	0.071
Nitrogen Oxides	D009589	Fisher's Exact Test	0.053	-
Sulfur Oxides	D013461	Fisher's Exact Test	-	-
Carbon Monoxide	D002248	Fisher's Exact Test	0.074	-
Ozone	D010126	Fisher's Exact Test	0.002	0.114
Polycyclic Aromatic Hydrocarbons	D011084	Fisher's Exact Test	<0.001	0.021 [a]
Phenanthrene	C031181	Fisher's Exact Test	0.125	-
Pyrene	C030984	Fisher's Exact Test	-	-
Lead	D007854	Fisher's Exact Test	<0.001	0.004 [a]
Mercury	D008628	Fisher's Exact Test	0.067	-
Cadmium	D002104	Fisher's Exact Test	0.021	-
Manganese	D008345	Fisher's Exact Test	0.057	-
Antimony	D000965	Fisher's Exact Test	0.010	0.710
Barium	D001464	Fisher's Exact Test	0.001	0.100
Cobalt	D003035	Fisher's Exact Test	0.001	0.048
Nickel	D009532	Fisher's Exact Test	0.019	-
Zinc	D015032	Fisher's Exact Test	0.001	0.091
Copper	D003300	Fisher's Exact Test	0.003	0.218
Iron	D007501	Fisher's Exact Test	0.003	0.212
Aluminum	D000535	Fisher's Exact Test	-	-
Lithium	D008094	Fisher's Exact Test	0.014	0.935
Magnesium	D008274	Fisher's Exact Test	-	-
Arsenic	D001151	Fisher's Exact Test	0.001	0.036 [a]

Pesticides	D010575	Fisher's Exact Test	0.030	-
Fungicides, Industrial	D005659	Fisher's Exact Test	-	-
Herbicides	D006540	Fisher's Exact Test	0.200	-
Insecticides	D007306	Fisher's Exact Test	-	-
Organophosphates	D010755	Fisher's Exact Test	0.005	0.351
Hydrocarbons, Chlorinated	D006843	Fisher's Exact Test	<0.001	<0.001 [a]
Carbamates	D002219	Fisher's Exact Test	<0.001	<0.001 [a]
Pyrethrins	D011722	Fisher's Exact Test	<0.001	0.001 [a]
Hexachlorobenzene	D006581	Fisher's Exact Test	0.133	-
Glyphosate	D000097797	Fisher's Exact Test	<0.001	0.031 [a]
DEET	D003671	Fisher's Exact Test	0.010	0.711
DDT	D003634	Fisher's Exact Test	0.020	-
Dichlorodiphenyl Dichloroethylene	D003633	Fisher's Exact Test	0.107	-
Parathion	D010278	Fisher's Exact Test	0.571	-
Methyl parathion	D008743	Fisher's Exact Test	-	-
Dieldrin	D004026	Fisher's Exact Test	<0.001	0.003 [a]
Aldrin	D000452	Fisher's Exact Test	0.009	0.644
Triclosan	D014260	Fisher's Exact Test	0.001	0.085
Fluorocarbons	D005466	Fisher's Exact Test	0.003	0.195
perfluorooctanoic acid	C023036	Fisher's Exact Test	0.025	-
perfluorooctane sulfonic acid	C076994	Fisher's Exact Test	<0.001	0.001 [a]
perfluorodecanoic acid	C036567	Fisher's Exact Test	<0.001	0.031 [a]
perfluoro-n-nonanoic acid	C101816	Fisher's Exact Test	0.046	-
perfluorohexanesulfonic acid	C471071	Fisher's Exact Test	0.243	-
Polychlorinated Biphenyls	D011078	Fisher's Exact Test	<0.001	0.018 [a]
2,3',4,4',5-pentachlorobiphenyl	C070055	Fisher's Exact Test	0.202	-
2,2',3',4,4',5-hexachlorobiphenyl	C029790	Fisher's Exact Test	0.008	0.583
2,4,5,2',4',5'-hexachlorobiphenyl	C014024	Fisher's Exact Test	0.002	0.171

2,2',3,3',4,4',5-heptachlorobiphenyl	C541131	Fisher's Exact Test	0.021	-
Halogenated Diphenyl Ethers	D055768	Standard Chi-Square	<0.001	<0.001 [a]
Dibutyl Phthalate	D003993	Fisher's Exact Test	<0.001	<0.001 [a]
Diethylhexyl Phthalate	D004051	Fisher's Exact Test	0.001	0.043 [a]
diethyl phthalate	C007379	Fisher's Exact Test	0.022	-
Plasticizers	D010968	Fisher's Exact Test	-	-
bisphenol A	C006780	Fisher's Exact Test	0.001	0.039 [a]
bisphenol B	C492482	Fisher's Exact Test	0.032	-
bisphenol S	C543008	Fisher's Exact Test	-	-
oxybenzone	C005290	Fisher's Exact Test	0.180	-
Plastics	D010969	Fisher's Exact Test	0.043	-
Microplastics	D000080545	Fisher's Exact Test	-	-
Chloroform	D002725	Fisher's Exact Test	0.077	-
Methylene chloride	D008752	Fisher's Exact Test	-	-
Tetrachloroethylene	D013750	Fisher's Exact Test	0.018	-
Trichloroethylene	D014241	Fisher's Exact Test	<0.001	0.007 [a]
Carbon Tetrachloride	D002251	Fisher's Exact Test	0.001	0.054

Note: [a] Statistically significant.

S2.1.5 Enrichment Analysis for Intellectual Disability (MESH: D008607)

Supplementary Table S20. Results of Enrichment Analysis for Intellectual Disability

Gene Sets	MeSH® ID	Statistical Method	Crude P-value	Corrected P-value
Air Pollutants	D000393	Standard Chi-Square	<0.001	<0.001 [a]
Particulate Matter	D052638	Standard Chi-Square	<0.001	<0.001 [a]
Nitrogen Oxides	D009589	Fisher's Exact Test	0.223	-
Sulfur Oxides	D013461	Fisher's Exact Test	0.237	-
Carbon Monoxide	D002248	Fisher's Exact Test	0.049	-
Ozone	D010126	Standard Chi-Square	<0.001	<0.001 [a]
Polycyclic Aromatic Hydrocarbons	D011084	Fisher's Exact Test	<0.001	0.002 [a]
Phenanthrene	C031181	Fisher's Exact Test	<0.001	<0.001 [a]
Pyrene	C030984	Fisher's Exact Test	0.024	-
Lead	D007854	Standard Chi-Square	<0.001	<0.001 [a]
Mercury	D008628	Fisher's Exact Test	0.004	0.253
Cadmium	D002104	Fisher's Exact Test	0.001	0.092
Manganese	D008345	Fisher's Exact Test	0.009	0.632
Antimony	D000965	Fisher's Exact Test	0.034	-
Barium	D001464	Fisher's Exact Test	0.228	-
Cobalt	D003035	Fisher's Exact Test	0.009	0.597
Nickel	D009532	Standard Chi-Square	<0.001	<0.001 [a]
Zinc	D015032	Standard Chi-Square	<0.001	<0.001 [a]
Copper	D003300	Fisher's Exact Test	<0.001	0.001 [a]
Iron	D007501	Fisher's Exact Test	0.047	-
Aluminum	D000535	Fisher's Exact Test	-	-
Lithium	D008094	Fisher's Exact Test	<0.001	0.012 [a]
Magnesium	D008274	Fisher's Exact Test	0.165	-
Arsenic	D001151	Fisher's Exact Test	<0.001	0.022 [a]

Pesticides	D010575	Fisher's Exact Test	<0.001	<0.001 [a]
Fungicides, Industrial	D005659	Fisher's Exact Test	0.004	0.254
Herbicides	D006540	Fisher's Exact Test	0.001	0.042 [a]
Insecticides	D007306	Fisher's Exact Test	0.127	-
Organophosphates	D010755	Fisher's Exact Test	<0.001	0.010 [a]
Hydrocarbons, Chlorinated	D006843	Standard Chi-Square	<0.001	<0.001 [a]
Carbamates	D002219	Fisher's Exact Test	<0.001	0.006 [a]
Pyrethrins	D011722	Standard Chi-Square	<0.001	<0.001 [a]
Hexachlorobenzene	D006581	Fisher's Exact Test	-	-
Glyphosate	D000097797	Standard Chi-Square	<0.001	<0.001 [a]
DEET	D003671	Fisher's Exact Test	0.001	0.046 [a]
DDT	D003634	Fisher's Exact Test	0.004	0.248
Dichlorodiphenyl Dichloroethylene	D003633	Standard Chi-Square	<0.001	<0.001 [a]
Parathion	D010278	Fisher's Exact Test	-	-
Methyl parathion	D008743	Fisher's Exact Test	0.220	-
Dieldrin	D004026	Fisher's Exact Test	<0.001	<0.001 [a]
Aldrin	D000452	Fisher's Exact Test	0.145	-
Triclosan	D014260	Standard Chi-Square	0.001	0.061
Fluorocarbons	D005466	Fisher's Exact Test	<0.001	0.004 [a]
perfluorooctanoic acid	C023036	Fisher's Exact Test	0.028	-
perfluorooctane sulfonic acid	C076994	Standard Chi-Square	<0.001	<0.001 [a]
perfluorodecanoic acid	C036567	Fisher's Exact Test	0.032	-
perfluoro-n-nonanoic acid	C101816	Standard Chi-Square	0.012	0.797
perfluorohexanesulfonic acid	C471071	Standard Chi-Square	<0.001	<0.001 [a]
Polychlorinated Biphenyls	D011078	Standard Chi-Square	<0.001	<0.001 [a]
2,3',4,4',5-pentachlorobiphenyl	C070055	Fisher's Exact Test	0.002	0.115
2,2',3',4,4',5-hexachlorobiphenyl	C029790	Standard Chi-Square	<0.001	<0.001 [a]
2,4,5,2',4',5'-hexachlorobiphenyl	C014024	Standard Chi-Square	<0.001	<0.001 [a]

2,2',3,3',4,4',5-heptachlorobiphenyl	C541131	Fisher's Exact Test	-	-
Halogenated Diphenyl Ethers	D055768	Standard Chi-Square	<0.001	<0.001 [a]
Dibutyl Phthalate	D003993	Standard Chi-Square	<0.001	<0.001 [a]
Diethylhexyl Phthalate	D004051	Fisher's Exact Test	<0.001	0.001 [a]
diethyl phthalate	C007379	Fisher's Exact Test	0.001	0.047 [a]
Plasticizers	D010968	Fisher's Exact Test	0.039	-
bisphenol A	C006780	Fisher's Exact Test	<0.001	<0.001 [a]
bisphenol B	C492482	Standard Chi-Square	0.001	0.041 [a]
bisphenol S	C543008	Fisher's Exact Test	0.056	-
oxybenzone	C005290	Fisher's Exact Test	<0.001	0.022 [a]
Plastics	D010969	Fisher's Exact Test	0.068	-
Microplastics	D000080545	Fisher's Exact Test	-	-
Chloroform	D002725	Fisher's Exact Test	0.310	-
Methylene chloride	D008752	Fisher's Exact Test	-	-
Tetrachloroethylene	D013750	Fisher's Exact Test	0.746	-
Trichloroethylene	D014241	Standard Chi-Square	<0.001	0.001 [a]
Carbon Tetrachloride	D002251	Fisher's Exact Test	<0.001	0.003 [a]

Note: [a] Statistically significant.

S2.1.6 Enrichment Analysis for Learning Disabilities (MESH: D007859)

Supplementary Table S21. Results of Enrichment Analysis for Learning Disabilities

Gene Sets	MeSH® ID	Statistical Method	Crude P-value	Corrected P-value
Air Pollutants	D000393	Fisher's Exact Test	<0.001	<0.001 [a]
Particulate Matter	D052638	Fisher's Exact Test	<0.001	<0.001 [a]
Nitrogen Oxides	D009589	Fisher's Exact Test	<0.001	<0.001 [a]
Sulfur Oxides	D013461	Fisher's Exact Test	<0.001	<0.001 [a]
Carbon Monoxide	D002248	Fisher's Exact Test	0.003	0.187
Ozone	D010126	Fisher's Exact Test	<0.001	<0.001 [a]
Polycyclic Aromatic Hydrocarbons	D011084	Fisher's Exact Test	<0.001	<0.001 [a]
Phenanthrene	C031181	Fisher's Exact Test	0.125	-
Pyrene	C030984	Fisher's Exact Test	0.125	-
Lead	D007854	Fisher's Exact Test	<0.001	<0.001 [a]
Mercury	D008628	Fisher's Exact Test	<0.001	<0.001 [a]
Cadmium	D002104	Fisher's Exact Test	<0.001	<0.001 [a]
Manganese	D008345	Fisher's Exact Test	<0.001	<0.001 [a]
Antimony	D000965	Fisher's Exact Test	<0.001	0.033 [a]
Barium	D001464	Fisher's Exact Test	0.055	-
Cobalt	D003035	Fisher's Exact Test	<0.001	<0.001 [a]
Nickel	D009532	Fisher's Exact Test	<0.001	<0.001 [a]
Zinc	D015032	Fisher's Exact Test	<0.001	<0.001 [a]
Copper	D003300	Fisher's Exact Test	<0.001	<0.001 [a]
Iron	D007501	Fisher's Exact Test	<0.001	<0.001 [a]
Aluminum	D000535	Fisher's Exact Test	<0.001	<0.001 [a]
Lithium	D008094	Fisher's Exact Test	<0.001	<0.001 [a]
Magnesium	D008274	Fisher's Exact Test	<0.001	<0.001 [a]
Arsenic	D001151	Fisher's Exact Test	<0.001	<0.001 [a]

Pesticides	D010575	Fisher's Exact Test	<0.001	<0.001 [a]
Fungicides, Industrial	D005659	Fisher's Exact Test	0.019	-
Herbicides	D006540	Fisher's Exact Test	0.001	0.098
Insecticides	D007306	Fisher's Exact Test	<0.001	0.028 [a]
Organophosphates	D010755	Fisher's Exact Test	<0.001	<0.001 [a]
Hydrocarbons, Chlorinated	D006843	Fisher's Exact Test	<0.001	<0.001 [a]
Carbamates	D002219	Fisher's Exact Test	<0.001	<0.001 [a]
Pyrethrins	D011722	Fisher's Exact Test	<0.001	<0.001 [a]
Hexachlorobenzene	D006581	Fisher's Exact Test	<0.001	<0.001 [a]
Glyphosate	D000097797	Fisher's Exact Test	<0.001	<0.001 [a]
DEET	D003671	Fisher's Exact Test	<0.001	<0.001 [a]
DDT	D003634	Fisher's Exact Test	<0.001	<0.001 [a]
Dichlorodiphenyl Dichloroethylene	D003633	Fisher's Exact Test	<0.001	<0.001 [a]
Parathion	D010278	Fisher's Exact Test	<0.001	<0.001 [a]
Methyl parathion	D008743	Fisher's Exact Test	0.001	0.093
Dieldrin	D004026	Fisher's Exact Test	<0.001	<0.001 [a]
Aldrin	D000452	Fisher's Exact Test	0.009	0.644
Triclosan	D014260	Fisher's Exact Test	<0.001	<0.001 [a]
Fluorocarbons	D005466	Fisher's Exact Test	<0.001	<0.001 [a]
perfluorooctanoic acid	C023036	Fisher's Exact Test	<0.001	<0.001 [a]
perfluorooctane sulfonic acid	C076994	Fisher's Exact Test	<0.001	<0.001 [a]
perfluorodecanoic acid	C036567	Fisher's Exact Test	<0.001	<0.001 [a]
perfluoro-n-nonanoic acid	C101816	Fisher's Exact Test	<0.001	0.003 [a]
perfluorohexanesulfonic acid	C471071	Fisher's Exact Test	<0.001	0.014 [a]
Polychlorinated Biphenyls	D011078	Fisher's Exact Test	<0.001	<0.001 [a]
2,3',4,4',5-pentachlorobiphenyl	C070055	Fisher's Exact Test	-	-
2,2',3',4,4',5-hexachlorobiphenyl	C029790	Fisher's Exact Test	<0.001	0.016 [a]
2,4,5,2',4',5'-hexachlorobiphenyl	C014024	Fisher's Exact Test	<0.001	<0.001 [a]

2,2',3,3',4,4',5-heptachlorobiphenyl	C541131	Fisher's Exact Test	-	-
Halogenated Diphenyl Ethers	D055768	Standard Chi-Square	<0.001	<0.001 [a]
Dibutyl Phthalate	D003993	Fisher's Exact Test	<0.001	<0.001 [a]
Diethylhexyl Phthalate	D004051	Fisher's Exact Test	<0.001	<0.001 [a]
diethyl phthalate	C007379	Fisher's Exact Test	<0.001	<0.001 [a]
Plasticizers	D010968	Fisher's Exact Test	0.066	-
bisphenol A	C006780	Fisher's Exact Test	<0.001	<0.001 [a]
bisphenol B	C492482	Fisher's Exact Test	0.119	-
bisphenol S	C543008	Fisher's Exact Test	<0.001	0.005 [a]
oxybenzone	C005290	Fisher's Exact Test	0.017	-
Plastics	D010969	Fisher's Exact Test	<0.001	<0.001 [a]
Microplastics	D000080545	Fisher's Exact Test	<0.001	0.016 [a]
Chloroform	D002725	Fisher's Exact Test	0.003	0.204
Methylene chloride	D008752	Fisher's Exact Test	<0.001	0.019 [a]
Tetrachloroethylene	D013750	Fisher's Exact Test	0.108	-
Trichloroethylene	D014241	Fisher's Exact Test	<0.001	<0.001 [a]
Carbon Tetrachloride	D002251	Fisher's Exact Test	<0.001	<0.001 [a]

Note: [a] Statistically significant.

S2.1.7 Enrichment Analysis for Motor Skills Disorders (MESH: D019957)

Supplementary Table S22. Results of Enrichment Analysis for Motor Skills Disorders

Gene Sets	MeSH® ID	Statistical Method	Crude P-value	Corrected P-value
Air Pollutants	D000393	Fisher's Exact Test	0.025	-
Particulate Matter	D052638	Fisher's Exact Test	<0.001	0.007 [a]
Nitrogen Oxides	D009589	Fisher's Exact Test	<0.001	0.017
Sulfur Oxides	D013461	Fisher's Exact Test	<0.001	0.006 [a]
Carbon Monoxide	D002248	Fisher's Exact Test	<0.001	0.034 [a]
Ozone	D010126	Fisher's Exact Test	0.031	-
Polycyclic Aromatic Hydrocarbons	D011084	Fisher's Exact Test	<0.001	0.001 [a]
Phenanthrene	C031181	Fisher's Exact Test	0.056	-
Pyrene	C030984	Fisher's Exact Test	-	-
Lead	D007854	Fisher's Exact Test	<0.001	0.001 [a]
Mercury	D008628	Fisher's Exact Test	0.001	0.050 [a]
Cadmium	D002104	Fisher's Exact Test	<0.001	0.004 [a]
Manganese	D008345	Fisher's Exact Test	<0.001	0.001 [a]
Antimony	D000965	Fisher's Exact Test	0.002	0.135
Barium	D001464	Fisher's Exact Test	0.024	-
Cobalt	D003035	Fisher's Exact Test	<0.001	0.004 [a]
Nickel	D009532	Fisher's Exact Test	0.012	0.805
Zinc	D015032	Fisher's Exact Test	0.001	0.038 [a]
Copper	D003300	Fisher's Exact Test	0.007	0.485
Iron	D007501	Fisher's Exact Test	0.007	0.476
Aluminum	D000535	Fisher's Exact Test	<0.001	0.020 [a]
Lithium	D008094	Fisher's Exact Test	0.003	0.179
Magnesium	D008274	Fisher's Exact Test	0.002	0.143
Arsenic	D001151	Fisher's Exact Test	0.003	0.191

Pesticides	D010575	Fisher's Exact Test	0.003	0.190
Fungicides, Industrial	D005659	Fisher's Exact Test	-	-
Herbicides	D006540	Fisher's Exact Test	0.092	-
Insecticides	D007306	Fisher's Exact Test	-	-
Organophosphates	D010755	Fisher's Exact Test	<0.001	<0.001 [a]
Hydrocarbons, Chlorinated	D006843	Fisher's Exact Test	<0.001	<0.001 [a]
Carbamates	D002219	Fisher's Exact Test	<0.001	<0.001 [a]
Pyrethrins	D011722	Fisher's Exact Test	<0.001	<0.001 [a]
Hexachlorobenzene	D006581	Fisher's Exact Test	0.060	-
Glyphosate	D000097797	Fisher's Exact Test	0.007	0.510
DEET	D003671	Fisher's Exact Test	<0.001	0.001 [a]
DDT	D003634	Fisher's Exact Test	0.004	0.273
Dichlorodiphenyl Dichloroethylene	D003633	Fisher's Exact Test	0.041	-
Parathion	D010278	Fisher's Exact Test	0.049	-
Methyl parathion	D008743	Fisher's Exact Test	<0.001	0.017 [a]
Dieldrin	D004026	Fisher's Exact Test	0.001	0.047 [a]
Aldrin	D000452	Fisher's Exact Test	-	-
Triclosan	D014260	Fisher's Exact Test	0.013	0.873
Fluorocarbons	D005466	Fisher's Exact Test	0.001	0.046 [a]
perfluorooctanoic acid	C023036	Fisher's Exact Test	<0.001	0.010 [a]
perfluorooctane sulfonic acid	C076994	Fisher's Exact Test	0.015	-
perfluorodecanoic acid	C036567	Fisher's Exact Test	0.063	-
perfluoro-n-nonanoic acid	C101816	Fisher's Exact Test	0.456	-
perfluorohexanesulfonic acid	C471071	Fisher's Exact Test	0.005	0.341
Polychlorinated Biphenyls	D011078	Fisher's Exact Test	<0.001	0.011 [a]
2,3',4,4',5-pentachlorobiphenyl	C070055	Fisher's Exact Test	0.005	0.341
2,2',3',4,4',5-hexachlorobiphenyl	C029790	Fisher's Exact Test	0.002	0.122
2,4,5,2',4',5'-hexachlorobiphenyl	C014024	Fisher's Exact Test	<0.001	0.015 [a]

2,2',3,3',4,4',5-heptachlorobiphenyl	C541131	Fisher's Exact Test	-	-
Halogenated Diphenyl Ethers	D055768	Fisher's Exact Test	0.002	0.167
Dibutyl Phthalate	D003993	Fisher's Exact Test	<0.001	0.003 [a]
Diethylhexyl Phthalate	D004051	Fisher's Exact Test	<0.001	0.003 [a]
diethyl phthalate	C007379	Fisher's Exact Test	0.095	-
Plasticizers	D010968	Fisher's Exact Test	-	-
bisphenol A	C006780	Fisher's Exact Test	<0.001	0.003 [a]
bisphenol B	C492482	Fisher's Exact Test	0.095	-
bisphenol S	C543008	Fisher's Exact Test	0.005	0.376
oxybenzone	C005290	Fisher's Exact Test	0.003	0.221
Plastics	D010969	Fisher's Exact Test	<0.001	0.001 [a]
Microplastics	D000080545	Fisher's Exact Test	0.010	0.667
Chloroform	D002725	Fisher's Exact Test	-	-
Methylene chloride	D008752	Fisher's Exact Test	0.054	-
Tetrachloroethylene	D013750	Fisher's Exact Test	0.217	-
Trichloroethylene	D014241	Fisher's Exact Test	<0.001	0.001 [a]
Carbon Tetrachloride	D002251	Fisher's Exact Test	<0.001	0.001 [a]

Note: [a] Statistically significant.

S2.2 Enriched Chemicals Selection and Pattern Evaluation

To reduce false-positive enrichment, contingency tables with expected gene frequencies ≤ 1 in any cell were excluded, and Fisher's exact tests were used to confirm chemical-disease gene enrichment associations identified by the standard Chi-square test in the primary analyses. For chemicals exhibiting significant associations with neurodevelopmental disorders through gene overlap, the proportional reporting ratio (PRR) was calculated to evaluate the direction and magnitude of enrichment (i.e., more overlapping genes than expected by chance) or depletion (i.e., fewer overlapping genes than expected by chance). A PRR > 1 indicates enrichment, a PRR < 1 indicates depletion, and PRR = 1 indicates no deviation from expectation.

2×2 Contingency Tables	Disease Gene	Not Disease Gene	Total Gene
Chemical Gene	A	B	A+B
Not Chemical Gene	C	D	C+D

$$Proportional\ reporting\ ratio\ (PRR) = \frac{(A/(A+B))}{(C/(C+D))}$$

Supplementary Table S23. Enriched Chemicals with Pattern Evaluation

Gene Sets	Proportional Reporting Ratio	95% Confidence Interval	Corrected P-value of Fisher's Exact Tests
**Autism Spectrum Disorder (MESH: D000067877)**			
Air Pollutants	3.56	(2.82, 4.50)	<0.001
Particulate Matter	7.78	(6.14, 9.85)	<0.001
Ozone	3.61	(2.87, 4.54)	<0.001
Lead	3.56	(2.79, 4.55)	<0.001
Mercury	7.55	(5.38, 10.61)	<0.001
Cadmium	5.26	(3.99, 6.94)	<0.001
Nickel	3.81	(2.98, 4.86)	<0.001
Zinc	4.64	(3.63, 5.94)	<0.001
Arsenic	7.21	(5.40, 9.63)	<0.001
Pesticides	7.73	(5.83, 10.25)	<0.001
Hydrocarbons, Chlorinated	8.50	(6.92, 10.45)	<0.001

Carbamates	8.91	(6.82, 11.63)	<0.001
Pyrethrins	8.37	(6.79, 10.33)	<0.001
Glyphosate	4.52	(3.55, 5.76)	<0.001
DEET	7.51	(5.78, 9.77)	<0.001
Dichlorodiphenyl Dichloroethylene	5.04	(3.98, 6.40)	<0.001
Parathion	6.64	(5.03, 8.76)	<0.001
Dieldrin	4.20	(3.08, 5.71)	<0.001
Triclosan	5.91	(4.75, 7.36)	<0.001
Fluorocarbons	9.41	(7.43, 11.91)	<0.001
perfluorooctane sulfonic acid	6.30	(5.08, 7.81)	<0.001
perfluoro-n-nonanoic acid	5.55	(4.32, 7.13)	<0.001
perfluorohexanesulfonic acid	4.94	(3.87, 6.30)	<0.001
Polychlorinated Biphenyls	6.16	(4.82, 7.88)	<0.001
2,3',4,4',5-pentachlorobiphenyl	3.10	(2.13, 4.51)	<0.001
2,2',3',4,4',5-hexachlorobiphenyl	3.05	(2.23, 4.18)	<0.001
2,4,5,2',4',5'-hexachlorobiphenyl	5.81	(4.55, 7.43)	<0.001
Halogenated Diphenyl Ethers	6.14	(4.99, 7.56)	<0.001
Dibutyl Phthalate	3.73	(3.00, 4.63)	<0.001
bisphenol B	3.60	(2.66, 4.88)	<0.001
Tetrachloroethylene	5.90	(4.21, 8.26)	<0.001
Trichloroethylene	4.74	(3.85, 5.83)	<0.001
Carbon Tetrachloride	7.65	(5.81, 10.06)	<0.001
**Autistic Disorder (MESH: D001321)**			
Air Pollutants	4.42	(3.40, 5.75)	<0.001
Particulate Matter	9.21	(7.04, 12.06)	<0.001
Ozone	4.12	(3.17, 5.36)	<0.001
Lead	6.89	(5.36, 8.86)	<0.001
Cadmium	7.56	(5.64, 10.11)	<0.001

Nickel	6.20	(4.79, 8.02)	<0.001
Zinc	6.61	(5.06, 8.63)	<0.001
Arsenic	9.43	(6.90, 12.89)	<0.001
Pesticides	9.20	(6.72, 12.62)	<0.001
Hydrocarbons, Chlorinated	8.28	(6.49, 10.57)	<0.001
Carbamates	11.27	(8.41, 15.12)	<0.001
Pyrethrins	8.71	(6.80, 11.15)	<0.001
Glyphosate	6.64	(5.11, 8.62)	<0.001
DEET	5.86	(4.17, 8.23)	<0.001
Dichlorodiphenyl Dichloroethylene	5.77	(4.40, 7.57)	<0.001
Parathion	7.61	(5.56, 10.42)	<0.001
Dieldrin	7.38	(5.47, 9.96)	<0.001
Triclosan	6.48	(5.02, 8.36)	<0.001
Fluorocarbons	7.05	(5.20, 9.56)	<0.001
perfluorooctane sulfonic acid	4.73	(3.62, 6.18)	<0.001
perfluoro-n-nonanoic acid	4.43	(3.22, 6.09)	<0.001
perfluorohexanesulfonic acid	4.10	(3.03, 5.56)	<0.001
Polychlorinated Biphenyls	8.12	(6.19, 10.64)	<0.001
2,3',4,4',5-pentachlorobiphenyl	2.80	(1.76, 4.45)	0.008
2,2',3',4,4',5-hexachlorobiphenyl	4.86	(3.54, 6.66)	<0.001
2,4,5,2',4',5'-hexachlorobiphenyl	6.68	(5.05, 8.83)	<0.001
Halogenated Diphenyl Ethers	7.22	(5.64, 9.25)	<0.001
Dibutyl Phthalate	6.72	(5.27, 8.57)	<0.001
bisphenol B	3.02	(2.06, 4.43)	<0.001
Trichloroethylene	4.89	(3.83, 6.24)	<0.001
Carbon Tetrachloride	8.37	(6.10, 11.48)	<0.001
**Intellectual Disability (MESH: D008607)**			
Air Pollutants	3.13	(2.12, 4.62)	<0.001

Particulate Matter	4.09	(2.55, 6.55)	<0.001
Ozone	3.03	(2.06, 4.46)	<0.001
Lead	5.59	(3.91, 7.98)	<0.001
Nickel	4.34	(2.96, 6.35)	<0.001
Zinc	4.35	(2.89, 6.54)	<0.001
Hydrocarbons, Chlorinated	5.18	(3.70, 7.25)	<0.001
Pyrethrins	5.49	(3.81, 7.91)	<0.001
Glyphosate	3.91	(2.60, 5.88)	<0.001
Dichlorodiphenyl Dichloroethylene	4.10	(2.73, 6.17)	<0.001
Triclosan	2.23	(1.41, 3.55)	0.112
perfluorooctane sulfonic acid	4.28	(2.94, 6.23)	<0.001
perfluoro-n-nonanoic acid	2.17	(1.23, 3.83)	0.857
perfluorohexanesulfonic acid	2.97	(1.87, 4.72)	0.003
Polychlorinated Biphenyls	5.03	(3.29, 7.69)	<0.001
2,2',3',4,4',5-hexachlorobiphenyl	4.00	(2.52, 6.35)	<0.001
2,4,5,2',4',5'-hexachlorobiphenyl	4.24	(2.74, 6.57)	<0.001
Halogenated Diphenyl Ethers	4.85	(3.47, 6.76)	<0.001
Dibutyl Phthalate	5.79	(4.15, 8.09)	<0.001
bisphenol B	2.68	(1.55, 4.65)	0.100
Trichloroethylene	2.29	(1.58, 3.33)	0.003
**Developmental Disabilities (MESH: D002658)**			
Halogenated Diphenyl Ethers	9.98	(4.67, 21.31)	<0.001
**Learning Disabilities (MESH: D007859)**			
Halogenated Diphenyl Ethers	7.48	(3.61, 15.53)	<0.001

S2.3 Cross-Validation of Enrichment Results

To assess the likelihood of false-positive associations in enrichment analyses involving NDD gene sets, we performed cross-validation using randomized testing. Specifically, 1,000 pseudo-chemical gene sets of varying sizes (50, 100, 250, 500, 1,000, 2,500, 5,000, and 10,000 genes) were generated to reflect the range of pollutant-associated gene counts observed in our dataset. Each randomized gene set was tested for enrichment against disorder-specific gene sets using Fisher's exact test.

A significance threshold of P-value < 0.01 was applied, and the frequency of significant results in these random tests was calculated to estimate the likelihood of false-positive findings occurring by chance.

Supplementary Table S24. Frequency of Significant Results in Random Enrichment Tests

Pseudo-Chemical Gene Sets	MeSH® ID	50	100	250	500	1000	2500	5000	100000
Attention Deficit Disorder with Hyperactivity	D001289	1	0	10 [a]	4	1	10 [a]	5	5
Autism Spectrum Disorder	D000067877	5	9	7	9	4	11 [a]	11 [a]	8
Autistic Disorder	D001321	4	4	2	4	4	5	5	4
Developmental Disabilities	D002658	0	4	0	6	7	11 [a]	4	8
Intellectual Disability	D008607	1	4	8	4	6	10 [a]	7	10 [a]
Learning Disabilities	D007859	0	1	1	3	4	8	7	4
Motor Skills Disorders	D019957	0	1	3	8	4	7	2	5

Note: [a] False-positive results were defined as those with unadjusted P-values < 0.01, corresponding to a frequency of ≥10 out of 1,000 random permutations.

The permutations for Autism Spectrum Disorder (D000067877, N = 357) and Motor Skills Disorders (D019957, N = 13) are presented in Figure 2 of the main text and are not repeated in the supplementary materials.

Supplementary Figure S2. Cross-Validation of Attention Deficit Disorder with Hyperactivity

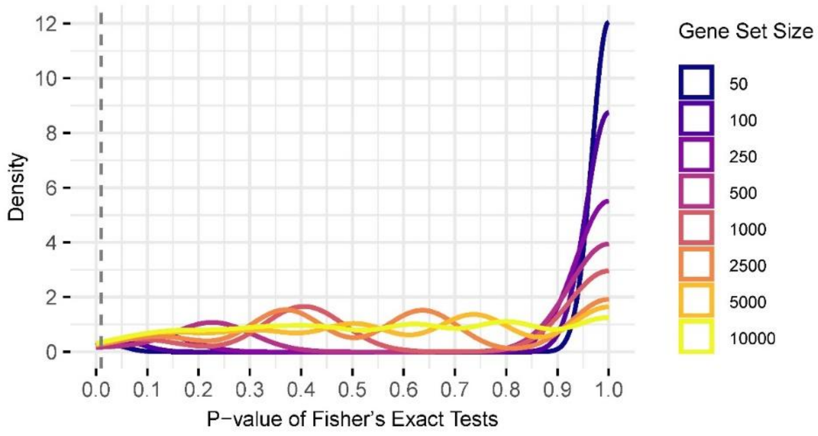

Supplementary Figure S3. Cross-Validation of Autistic Disorder

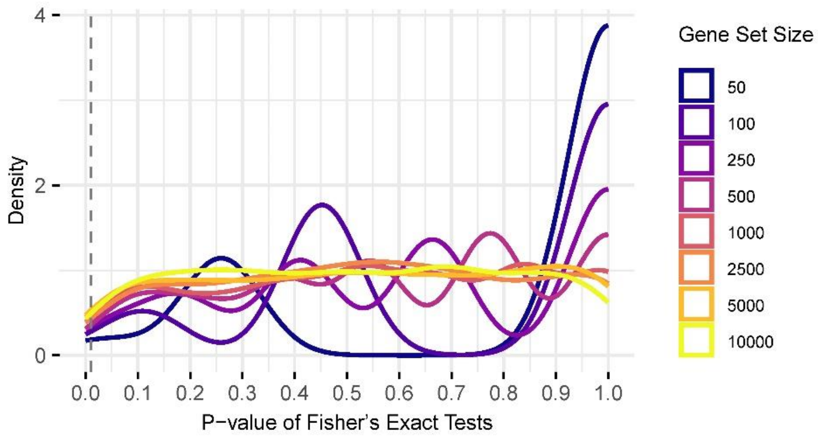

Supplementary Figure S4. Cross-Validation of Developmental Disabilities

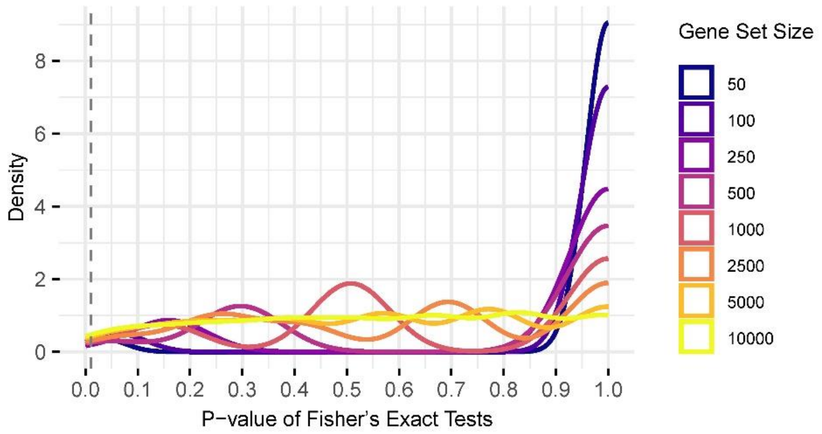

Supplementary Figure S5. Cross-Validation of Intellectual Disability

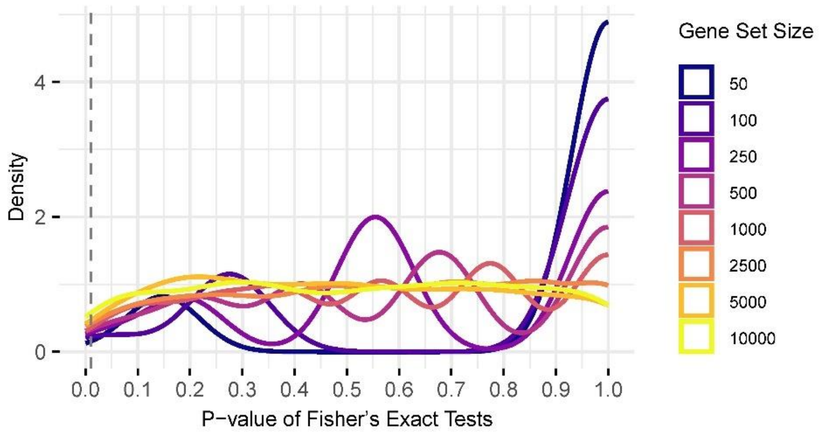

Supplementary Figure S6. Cross-Validation of Learning Disabilities

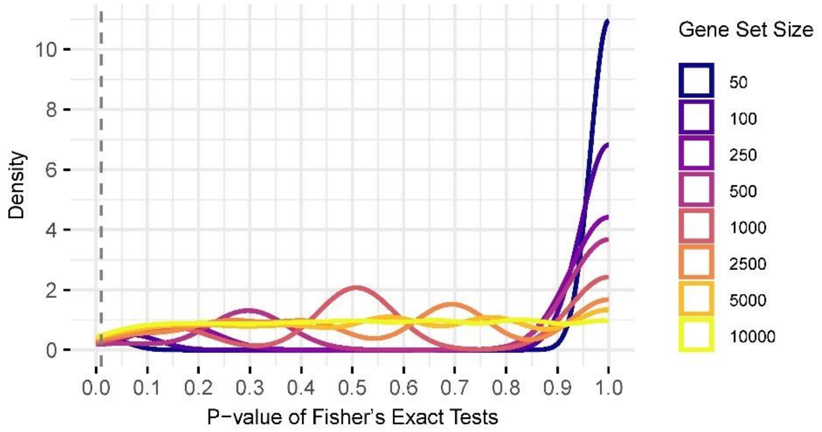

S2.4 Functional Annotation for Pollutant-Disorder Gene Sets of Autism Spectrum Disorder

Gene Ontology (GO) terms consist of three categories: biological process (BP), cellular component (CC), and molecular function (MF). In this study, we focused on the biological functions of shared genes, so GO term annotation was primarily conducted using the BP_DIRECT category for biological annotation. The significantly enriched GO terms are listed below.

S2.4.1 Significant GO Biological Processes for Pollutant-Disorder Gene Sets of Autism Spectrum Disorder

Gene Ontology (GO) terms consist of three categories: biological process (BP), cellular component (CC), and molecular function (MF). In this study, we focused on the biological functions of shared genes, so GO term annotation was primarily conducted using the BP_DIRECT category for biological annotation. All significant GO Biological Processes are listed below. If fewer than five significant GO Biological Processes are identified, the top five Biological Processes ranked by Bonferroni-corrected P-values will be presented.

Supplementary Table S25. Air Pollutants (MeSH: D000393) to Autism Spectrum Disorder (MeSH: D000067877), Bonferroni-Corrected P-values < 0.05

Term	Count	%	Genes	Fold Enrichment	Bonferroni P-values
GO:0006805~xenobiotic metabolic process	16	17.0	ABCC3, NQO1, TPMT, GSTP1, EPHX1, FMO1, FMO2, AHR, SULT1B1, ALDH3A1, GSTA3, CYP2D6, GSTA2, CYP1A1, AOX1, CBR3	28.1	1.29E-14
GO:0098869~cellular oxidant detoxification	7	7.4	GPX2, GSTO1, TXNRD1, GSTP1, CP, PTGES, PTGS1	20.1	1.40E-03
GO:0006749~glutathione metabolic process	6	6.4	GSTM1, GSTO1, GSTA3, TXNRD1, GSTP1, GSTA2	29.6	1.85E-03
GO:0010629~negative regulation of gene expression	11	11.7	NOTCH2, MEF2C, DNMT1, NOTCH1, CLDN3, LRP1, NOS2, NRXN1, MIF, IQGAP3, FOXP1	7.5	2.05E-03
GO:0006693~prostaglandin metabolic process	5	5.3	GSTM1, GSTP1, AKR1C3, PTGR1, PTGES	48.2	3.40E-03
GO:0042178~xenobiotic catabolic process	5	5.3	GSTM1, TPMT, GSTO1, CYP2D6, NOS1	46.1	4.11E-03
GO:0032496~response to lipopolysaccharide	8	8.5	NQO1, NOTCH1, NOS2, NOS3, CYP1A1, FMO1, NOS1, FOXP1	12.2	4.41E-03
GO:0010628~positive regulation of gene expression	12	12.8	AFDN, MEF2C, DNMT1, NOTCH1, CLDN3, TFRC, NOS3, NRXN1, EPHX2, HFE, DNMT3B, IQGAP3	5.0	2.73E-02
GO:0009725~response to hormone	5	5.3	NQO1, NOS2, NOS3, DHCR24, NOS1	27.9	3.20E-02

Supplementary Table S26. Particulate Matter (MeSH: D052638) to Autism Spectrum Disorder (MeSH: D000067877), Bonferroni-Corrected P-values < 0.05

Term	Count	%	Genes	Fold Enrichment	Bonferroni P-values

GO:0006805~xenobiotic metabolic process	21	24.1	ABCC3, NQO1, CBR1, ABCB1, UGT1A1, GSTP1, EPHX1, FMO2, AHR, CYP3A5, ALDH3A1, CYP26B1, CYP2B6, GSTA3, CYP2S1, GSTA2, CYP1A2, CYP1A1, AOX1, CBR3, UGT1A6	39.4	1.63E-23
GO:0006693~prostaglandin metabolic process	8	9.2	GSTM1, GSTP1, CYP2S1, AKR1C3, AKR1C2, COMT, PTGR1, PTGES	82.5	4.36E-09
GO:0032496~response to lipopolysaccharide	10	11.5	TJP1, NQO1, NOTCH1, UGT1A1, NOS2, NOS3, DIO2, CYP1A1, NOS1, LOXL1	16.3	1.11E-05
GO:0006749~glutathione metabolic process	7	8.0	GSTM1, GSTO1, TXNRD2, GSTA3, TXNRD1, GSTP1, GSTA2	36.9	3.56E-05
GO:0098869~cellular oxidant detoxification	8	9.2	GPX2, GSTO1, TXNRD2, TXNRD1, GSTP1, CP, PTGES, PTGS1	24.5	3.84E-05
GO:0042178~xenobiotic catabolic process	6	6.9	GSTM1, CYP2B6, GSTO1, CYP1A2, NOS1, CYP3A5	59.2	5.12E-05
GO:0008202~steroid metabolic process	7	8.0	CYP2B6, UGT1A1, AKR1C3, CYP1A1, AKR1C2, DHCR24, CYP3A5	31.8	9.06E-05
GO:0042572~retinol metabolic process	7	8.0	ALDH1A3, AKR1B10, CYP1A2, ALDH1A1, AKR1C3, CYP1A1, CYP3A5	29.4	1.45E-04
GO:0045454~cell redox homeostasis	6	6.9	NQO1, NOS2, TXNRD2, NOS3, TXNRD1, NOS1	30.9	1.54E-03
GO:0042573~retinoic acid metabolic process	5	5.7	ALDH1A3, CYP26B1, UGT1A1, CYP2S1, CYP3A5	45.4	4.59E-03
GO:0009725~response to hormone	5	5.7	NQO1, NOS2, NOS3, DHCR24, NOS1	29.8	2.54E-02
GO:0009636~response to toxic substance	6	6.9	NQO1, EPHX2, EPHX1, DNMT3A, AHR, COMT	17.4	2.63E-02
GO:0150104~transport across blood-brain barrier	6	6.9	ABCC3, ABCB1, TFRC, SLC2A3, SLC1A4, ABCG2	16.2	3.74E-02

Supplementary Table S27. Lead (MeSH: D007854) to Autism Spectrum Disorder (MeSH: D000067877), Bonferroni-Corrected P-values < 0.05

Term	Count	%	Genes	Fold Enrichment	Bonferroni P-values
GO:0006805~xenobiotic metabolic process	11	13.4	ABCC3, NQO1, CYP2C9, ABCB1, GSTP1, GSTA2, EPHX1, CYP1A2, CYP1A1, AHR, CES2	21.9	6.84E-08
GO:0042178~xenobiotic catabolic process	6	7.3	CYP2C9, GSTM1, GSTO1, CYP1A2, NOS1, CRYZ	62.8	3.33E-05
GO:0150104~transport across blood-brain barrier	8	9.8	ABCC3, ABCA2, ABCB1, LRP1, TFRC, SLC1A1, SLC2A3, ATP1B2	22.9	5.40E-05
GO:0042446~hormone biosynthetic process	5	6.1	CHST8, HFE, CYP1A2, DIO2, CYP1A1	66.8	7.87E-04
GO:0032496~response to lipopolysaccharide	8	9.8	ALAD, TJP1, NQO1, NOS2, NOS3, DIO2, CYP1A1, NOS1	13.9	1.70E-03
GO:0006693~prostaglandin metabolic process	5	6.1	GSTM1, GSTP1, AKR1C2, PTGR1, CES2	54.7	1.86E-03
GO:0006749~glutathione metabolic process	5	6.1	GSTM1, GSTO1, TXNRD1, GSTP1, GSTA2	28.0	2.89E-02
GO:0045454~cell redox homeostasis	5	6.1	NQO1, NOS2, NOS3, TXNRD1, NOS1	27.3	3.17E-02
GO:1903926~cellular response to bisphenol A	3	3.7	DNMT1, SLC1A1, DNMT3A	240.7	4.95E-02

Supplementary Table S28. Mercury (MeSH: D008628) to Autism Spectrum Disorder (MeSH: D000067877), Bonferroni-Corrected P-values < 0.05

Term	Count	%	Genes	Fold Enrichment	Bonferroni P-values
GO:0006805~xenobiotic metabolic process	8	22.9	NQO1, ABCB1, CYP2U1, GSTP1, GSTA2, CYP1A2, CYP1A1, AHR	37.3	6.71E-07
GO:0006749~glutathione metabolic process	5	14.3	GSTM1, TXNRD2, TXNRD1, GSTP1, GSTA2	65.6	4.52E-04
GO:0009410~response to xenobiotic stimulus	7	20.0	ALAD, TJP1, ABCB1, TXNRD2, AHR, COMT, SLC6A4	15.5	2.23E-03
GO:0045454~cell redox homeostasis	4	11.4	NQO1, NOS2, TXNRD2, TXNRD1	51.3	3.04E-02
GO:0032496~response to lipopolysaccharide	5	14.3	ALAD, TJP1, NQO1, NOS2, CYP1A1	20.3	4.74E-02

Supplementary Table S29. Cadmium (MeSH: D002104) to Autism Spectrum Disorder (MeSH: D000067877), Bonferroni-Corrected P-values < 0.05

Term	Count	%	Genes	Fold Enrichment	Bonferroni P-values
GO:0032496~response to lipopolysaccharide	8	13.8	ALAD, TJP1, NQO1, NOS2, NOS3, CYP1A1, NOS1, ADH5	19.6	1.18E-04
GO:0006693~prostaglandin metabolic process	5	8.6	GSTM1, GSTP1, AKR1C3, AKR1C2, COMT	77.3	3.56E-04
GO:0006805~xenobiotic metabolic process	7	12.1	NQO1, ABCB1, GSTA3, GSTP1, EPHX1, CYP1A1, AHR	19.7	1.07E-03
GO:0098869~cellular oxidant detoxification	6	10.3	GPX2, GSTO1, TXNRD1, GSTP1, GPX5, PTGS1	27.6	1.88E-03
GO:0006749~glutathione metabolic process	5	8.6	GSTM1, GSTO1, GSTA3, TXNRD1, GSTP1	39.6	5.73E-03
GO:0045454~cell redox homeostasis	5	8.6	NQO1, NOS2, NOS3, TXNRD1, NOS1	38.7	6.29E-03
GO:0042572~retinol metabolic process	5	8.6	AKR1B10, ALDH1A1, AKR1C3, CYP1A1, ADH6	31.5	1.43E-02
GO:0006979~response to oxidative stress	6	10.3	ALAD, NQO1, GPX2, GPX5, COMT, PTGS1	16.6	2.24E-02
GO:0006809~nitric oxide biosynthetic process	4	6.9	NQO1, NOS2, NOS3, NOS1	56.7	3.52E-02

Supplementary Table S30. Zinc (MeSH: D015032) to Autism Spectrum Disorder (MeSH: D000067877), Bonferroni-Corrected P-values < 0.05

Term	Count	%	Genes	Fold Enrichment	Bonferroni P-values
GO:0006805~xenobiotic metabolic process	15	19.0	NQO1, AOC2, ABCB1, UGT1A1, EPHX1, FMO2, AHR, FMO4, CYP3A5, CYP2D6, CYP2R1, CYP1A2, AOX1, CYP2F1, ADA	31.0	3.71E-14
GO:0042572~retinol metabolic process	8	10.1	ADH4, CYP27C1, CYP2D6, CYP1A2, ALDH1A1, AKR1C3, CYP3A5, ADH6	37.0	1.84E-06
GO:0009636~response to toxic substance	7	8.9	NQO1, EPHX1, DNMT3A, AHR, CYP2F1, DHRS2, SLC6A4	22.4	6.75E-04

GO:0150104~transport across blood-brain barrier	7	8.9	SLCO1C1, ABCB1, TFRC, INSR, SLC1A1, SLC2A3, SLC1A4	20.8	1.05E-03
GO:0032496~response to lipopolysaccharide	8	10.1	ALAD, TJP1, NQO1, NOTCH1, UGT1A1, NOS2, NOS3, ADH5	14.4	1.35E-03
GO:0042178~xenobiotic catabolic process	5	6.3	CYP2D6, CYP1A2, FMO4, CYP3A5, CRYZ	54.3	1.98E-03
GO:0008210~estrogen metabolic process	5	6.3	UGT1A1, CYP2D6, CYP1A2, CHST10, CYP3A5	39.0	7.82E-03
GO:0007613~memory	6	7.6	BDNF, INSR, SLC1A1, CIC, SHANK3, SLC6A4	17.2	2.52E-02
GO:0046185~aldehyde catabolic process	3	3.8	ALDH2, ALDH3B1, AKR1A1	249.8	4.73E-02

Supplementary Table S31. Arsenic (MeSH: D001151) to Autism Spectrum Disorder (MeSH: D000067877), Bonferroni-Corrected P-values < 0.05

Term	Count	%	Genes	Fold Enrichment	Bonferroni P-values
GO:0006805~xenobiotic metabolic process	12	23.5	ABCC3, NQO1, BCHE, CYP26B1, ABCB1, UGT1A1, CYP2U1, GSTP1, CYP1A1, FMO2, AHR, UGT1A6	38.4	8.25E-12
GO:0032496~response to lipopolysaccharide	8	15.7	ALAD, NQO1, NOTCH1, UGT1A1, NOS2, NOS3, CYP1A1, FOXP1	22.3	5.39E-05
GO:0008202~steroid metabolic process	5	9.8	SULT2B1, UGT1A1, AKR1C3, CYP1A1, AKR1C2	38.7	7.22E-03
GO:0006693~prostaglandin metabolic process	4	7.8	GSTM1, GSTP1, AKR1C3, AKR1C2	70.4	2.12E-02
GO:0006749~glutathione metabolic process	4	7.8	GSTM1, GSTO1, TXNRD1, GSTP1	36.0	1.53E-01

Supplementary Table S32. Glyphosate (MeSH: D000097797) to Autism Spectrum Disorder (MeSH: D000067877), Bonferroni-Corrected P-values < 0.05

Term	Count	%	Genes	Fold Enrichment	Bonferroni P-values
GO:0006805~xenobiotic metabolic process	11	13.1	NQO1, CYP2C9, BCHE, UGT1A10, ABCB1, TPMT, CYP2S1, GSTA2, CYP1A1, FMO1, AHR	21.6	8.41E-08
GO:0035176~social behavior	8	9.5	OXTR, CHD8, NRXN1, NRXN2, AVPR1A, SHANK3, DRD4, SLC6A4	34.6	3.15E-06
GO:0150104~transport across blood-brain barrier	7	8.3	SLCO1C1, ABCA2, SLC22A5, ABCB1, AVPR1A, SLC16A2, ABCG2	19.8	1.48E-03
GO:0042178~xenobiotic catabolic process	5	6.0	CYP2C9, GSTM1, TPMT, GSTO1, CRYZ	51.7	2.55E-03
GO:0032496~response to lipopolysaccharide	7	8.3	TJP1, NQO1, NOTCH1, NOS2, DIO2, CYP1A1, FMO1	12.0	2.70E-02

Supplementary Table S33. Perfluorooctane Sulfonic Acid (MeSH: C076994) to Autism Spectrum Disorder (MeSH: D000067877), Bonferroni-Corrected P-values < 0.05

Term	Count	%	Genes	Fold Enrichment	Bonferroni P-values
GO:0006805~xenobiotic metabolic process	22	17.6	CYP2J2, ABCC3, NQO1, CBR1, UGT1A1, TPMT, GSTP1, EPHX1, CYP2C19, CYP3A7, SULT1B1, CYP2C8, CYP2B6, GSTA3, CYP2D6, GSTA2, CYP1A2, CYP1A1, AOX1, UGT2B7, CES2, CBR3	29.7	6.40E-22
GO:0150104~transport across blood-brain barrier	14	11.2	ABCC3, ABCA2, SLC16A1, SLC22A5, LRP1, TFRC, INSR, SLC1A1, SLC1A4, AVPR1A, SLCO1C1, SLC16A2, SLC19A1, ABCG2	27.2	4.69E-12
GO:0008210~estrogen metabolic process	10	8.0	UGT2B10, CYP2C8, UGT1A1, CYP2D6, HSD17B1, CYP1A2, CYP1A1, UGT2A3, UGT2B7, CYP3A7	51.0	2.85E-10
GO:0042178~xenobiotic catabolic process	9	7.2	CYP2C8, GSTM1, CYP2B6, TPMT, GSTO1, CYP2D6, CYP1A2, CYP2C19, CRYZ	63.8	9.14E-10
GO:0019373~epoxygenase P450 pathway	8	6.4	CYP2J2, CYP2C8, CYP2B6, CYP1A2, CYP4A22, CYP4A11, CYP1A1, CYP2C19	62.1	3.81E-08
GO:0097267~omega-hydroxylase P450 pathway	6	4.8	CYP2C8, CYP1A2, CYP4A22, CYP4A11, CYP1A1, CYP2C19	81.5	7.98E-06
GO:0006749~glutathione metabolic process	8	6.4	GSTM1, GSTO1, GSTA3, TXNRD1, GSTP1, GSTA2, GSTT2, GSTM5	30.3	9.45E-06
GO:0008202~steroid metabolic process	8	6.4	CYP2C8, CYP2B6, UGT1A1, CYP2D6, CYP1A1, DHCR24, CYP2C19, CYP3A7	26.1	2.83E-05
GO:0042572~retinol metabolic process	8	6.4	ADH4, CYP2C8, CYP2D6, ADH1A, CYP1A2, ALDH1A1, CYP1A1, CYP3A7	24.2	4.91E-05
GO:0016125~sterol metabolic process	6	4.8	CYP39A1, CYP27A1, CYP11B2, CYP11A1, CYP19A1, CYP7A1	46.6	1.96E-04
GO:0006693~prostaglandin metabolic process	6	4.8	GSTM1, GSTP1, COMT, PTGR1, CES2, PTGES	44.5	2.53E-04
GO:0002933~lipid hydroxylation	4	3.2	CYP2C8, CYP4A22, CYP1A1, CYP3A7	93.2	1.02E-02
GO:0009636~response to toxic substance	7	5.6	NQO1, PON3, EPHX2, PON2, EPHX1, DNMT3A, COMT	14.6	1.09E-02
GO:0070168~negative regulation of biomineral tissue development	4	3.2	NOTCH1, HEY1, NOS3, SOX9	72.5	2.42E-02
GO:0032496~response to lipopolysaccharide	8	6.4	ALAD, TJP1, NQO1, NOTCH1, UGT1A1, NOS2, NOS3, CYP1A1	9.4	3.05E-02
GO:0070989~oxidative demethylation	4	3.2	CYP2C8, CYP2D6, CYP1A2, CYP3A7	65.2	3.42E-02
GO:0006694~steroid biosynthetic process	5	4.0	HSD17B1, HSD3B1, CYP1A1, CYP19A1, CYP3A7	28.1	3.68E-02

Supplementary Table S34. Perfluorohexanesulfonic Acid (MeSH: C471071) to Autism Spectrum Disorder (MeSH: D000067877), Bonferroni-Corrected P-values < 0.05

Term	Count	%	Genes	Fold Enrichment	Bonferroni P-values
GO:0006805~xenobiotic metabolic process	18	22.0	NQO1, CBR1, AOC2, UGT1A1, TPMT, GSTP1, FMO1, FMO2, CYP2C19, CYP3A5, CYP3A7, SULT1C3, CYP2B6, CYP2U1, CYP2D6, CYP2S1, AOX1, ADA	35.8	5.72E-19

GO:0042178~xenobiotic catabolic process	7	8.5	GSTM1, CYP2B6, TPMT, CYP2D6, NOS1, CYP2C19, CYP3A5	73.2	3.12E-07
GO:0032496~response to lipopolysaccharide	8	9.8	NQO1, UGT1A1, NOS2, NOS3, DIO2, FMO1, NOS1, LOXL1	13.9	1.37E-03
GO:0008202~steroid metabolic process	6	7.3	CYP2B6, UGT1A1, CYP2D6, CYP2C19, CYP3A5, CYP3A7	28.9	1.55E-03
GO:0051122~hepoxilin biosynthetic process	4	4.9	GSTM1, GSTP1, ALOX12B, ALOX12	107.0	4.52E-03
GO:0008210~estrogen metabolic process	5	6.1	UGT1A1, CYP2D6, HSD17B1, CYP3A5, CYP3A7	37.6	7.13E-03
GO:0006082~organic acid metabolic process	4	4.9	CYP2U1, FMO1, FMO2, CYP2C19	74.0	1.51E-02
GO:0150104~transport across blood-brain barrier	6	7.3	ABCA2, SLC22A5, AVPR1A, SLC16A2, SLC19A1, ABCG2	17.2	2.01E-02
GO:0042446~hormone biosynthetic process	4	4.9	CYP2D6, HFE, DIO2, DIO3	53.5	4.19E-02

Supplementary Table S35. Polychlorinated Biphenyls (MeSH: D011078) to Autism Spectrum Disorder (MeSH: D000067877), Bonferroni-Corrected P-values < 0.05

Term	Count	%	Genes	Fold Enrichment	Bonferroni P-values
GO:0006805~xenobiotic metabolic process	19	24.1	ABCC3, NQO1, UGT1A1, GSTP1, EPHX1, FMO1, AHR, SULT1B1, ALDH3A1, CYP2B6, CYP21A2, GSTA3, GSTA2, CYP2R1, CYP1A2, CYP1A1, AOX1, CES3, UGT1A6	39.7	4.62E-21
GO:0032496~response to lipopolysaccharide	9	11.4	TJP1, NQO1, CYP27B1, NOTCH1, UGT1A1, NOS2, CYP1A1, FMO1, FOXP1	16.4	6.66E-05
GO:0009636~response to toxic substance	7	8.9	NQO1, PON3, EPHX1, DNMT3A, AHR, COMT, SLC6A4	22.7	5.73E-04
GO:0016125~sterol metabolic process	5	6.3	CYP11B2, CYP21A2, CYP11A1, CYP19A1, CYP7A1	60.2	1.17E-03
GO:0008202~steroid metabolic process	6	7.6	SULT2B1, CYP2B6, UGT1A1, CYP21A2, CYP1A1, AKR1C2	30.4	1.41E-03
GO:0006694~steroid biosynthetic process	5	6.3	CYP21A2, HSD17B1, HSD3B1, CYP1A1, CYP19A1	43.6	4.54E-03
GO:0042359~vitamin D metabolic process	4	5.1	CYP27B1, CYP11A1, CYP2R1, CYP1A1	101.2	6.45E-03
GO:0006704~glucocorticoid biosynthetic process	4	5.1	CYP27B1, CYP11B2, CYP21A2, CYP11A1	101.2	6.45E-03
GO:0006749~glutathione metabolic process	5	6.3	GSTM1, GSTA3, GSTP1, GSTA2, GSTT2	29.4	2.24E-02
GO:0042448~progesterone metabolic process	4	5.1	CYP21A2, CYP1A2, CYP1A1, AKR1C2	59.5	3.53E-02

Supplementary Table S36. 2,4,5,2',4',5'-hexachlorobiphenyl (MeSH: C014024) to Autism Spectrum Disorder (MeSH: D000067877), Bonferroni-Corrected P-values < 0.05

Term	Count	%	Genes	Fold Enrichment	Bonferroni P-values

GO:0006805~xenobiotic metabolic process	18	22.5	ABCC3, NQO1, AOC2, UGT1A1, TPMT, EPHX1, FMO1, FMO2, AHR, SULT1B1, CYP2U1, GSTA2, CYP2R1, CYP1A2, CYP1A1, AOX1, CES3, CES2	37.2	3.04E-19
GO:0032496~response to lipopolysaccharide	10	12.5	TJP1, NQO1, CYP27B1, NOTCH1, UGT1A1, NOS2, DIO2, CYP1A1, FMO1, FOXP1	18.0	3.51E-06
GO:0006693~prostaglandin metabolic process	6	7.5	GSTM1, AKR1C3, AKR1C2, COMT, PTGR1, CES2	68.1	1.87E-05
GO:0016125~sterol metabolic process	5	6.3	CYP39A1, CYP11B2, CYP11A1, CYP19A1, CYP7A1	59.5	1.13E-03
GO:0042359~vitamin D metabolic process	4	5.0	CYP27B1, CYP11A1, CYP2R1, CYP1A1	99.9	6.15E-03
GO:0009636~response to toxic substance	6	7.5	NQO1, EPHX2, EPHX1, AHR, COMT, SLC6A4	19.2	1.25E-02
GO:0006082~organic acid metabolic process	4	5.0	CYP2U1, CYP2R1, FMO1, FMO2	76.9	1.45E-02
GO:0006749~glutathione metabolic process	5	6.3	GSTM1, TXNRD1, GSTA2, GSTT2, GSTM5	29.0	2.17E-02
GO:0042448~progesterone metabolic process	4	5.0	CYP1A2, AKR1C3, CYP1A1, AKR1C2	58.8	3.37E-02
GO:0008202~steroid metabolic process	5	6.3	SULT2B1, UGT1A1, AKR1C3, CYP1A1, AKR1C2	25.0	3.92E-02

S2.4.2 Significant DisGeNET Associations for Pollutant-Disorder Gene Sets of Autism Spectrum Disorder

All significant DisGeNET Associations are listed below, ranked by Bonferroni-corrected P-values.

Supplementary Table S37. DisGeNET Associations for Autism Spectrum Disorder, Bonferroni-Corrected P-values < 0.05

Term	Count	%	Genes	Fold Enrichment	Bonferroni P-values
C1510586~Autism Spectrum Disorders	62	22.1	GABRB3, DPP10, CNTNAP2, RYR2, OXTR, LAMC3, HFE, CHD8, ITGB3, ROGDI, CHD2, UNC80, HEY1, LRRTM3, CA2, DNMT3B, DIP2A, UPP2, SOX9, JARID2, SLC16A2, CIC, SOX5, GTF2I, SCN1A, MEF2C, NSUN2, DNMT3A, CIRBP, DIO2, EN2, TET1, DIO3, AVPR1A, FOXP1, IL1RAPL1, DPYD, TET3, RARB, SHANK3, CTTNBP2, SFSWAP, NRXN1, ANKRD11, SLC1A1, NRXN2, IQGAP3, RAI1, DPP6, RELN, PTCHD1, NTSR1, LRRN3, PCDH9, BDNF, EPHX2, DHCR24, GRIN2B, USH2A, DLG4, PAH, CEP41	29.2	4.17E-78
C0004352~Autistic Disorder	34	12.1	GABRB3, CNTNAP2, OXTR, GSTP1, CHD8, NRXN1, ITGB3, NRXN2, AQP4, ADRB2, COMT, CYP19A1, SLC6A4, RELN, PTCHD1, SLC19A1, ADSL, MEF2C, GSTM1, NOS2, BDNF, EN2, MTHFR, AVPR1A, MIF, CP, GRIN2B, FOXP2, APC, IL1RAPL1, DPYD, MET, SHANK3, ADA	5.2	1.39E-11
C0036341~Schizophrenia	61	21.8	GABRB3, ACHE, CNTNAP2, OXTR, ITGB3, MTR, AQP4, COMT, KIF17, SLC6A4, CYP2D6, DNMT3B, NOS1, JARID2, CGNL1, SOX5, AKR1A1, EN2, TET1, DIO3, AVPR1A, FOXP2, SYN1, ALDH3A1, DPYD, ALDH1A1, MET, SHANK3, DNMT1, ABCB1, LRP1, GSTP1, NRXN1, NOTCH4, CHAT,	2.8	3.75E-10

			SLC1A1, GSTT2, NRXN2, ALOX12, CYP3A5, PTGS1, RAI1, RELN, CBS, ALDH3B1, DRD4, NTSR1, NQO2, GSTM1, JAG1, BDNF, EPHX2, B3GAT2, MTHFR, CP, GRIN2B, GFAP, APC, DLG4, PAH, CYP1A2		
C0027765~nervous system disorder	14	5.0	ACHE, NOTCH1, ABCB1, BDNF, MTHFR, ADRB2, GFAP, CYP2C9, CYP2C8, DPYD, NOS1, GUSB, SLC19A1, ABCG2	10.6	6.69E-07
C0013221~Drug toxicity	16	5.7	ACHE, BCHE, ABCB1, UGT1A1, EPHX2, MTHFR, MTR, CP, CYP2C9, CYP2C8, CYP2D6, CYP1A2, SLCO2A1, SLC19A1, CES2, ABCG2	7.6	3.42E-06
C0041755~Adverse reaction to drug	16	5.7	ACHE, BCHE, ABCB1, UGT1A1, EPHX2, MTHFR, MTR, CP, CYP2C9, CYP2C8, CYP2D6, CYP1A2, SLCO2A1, SLC19A1, CES2, ABCG2	7.6	3.42E-06
C0001969~Alcoholic Intoxication	11	3.9	BCHE, ALDH2, BDNF, ADH1B, ALDH1A1, CHAT, AKR1A1, MTHFR, MTR, DRD4, SLC6A4	13.8	5.73E-06
C0001973~Alcoholic Intoxication, Chronic	26	9.3	GABRB3, CNTNAP2, ADH1B, ADH1A, COMT, ADH5, ADH6, SLC6A4, ALDH3B2, ADH4, CYP2B6, ALDH2, MPDZ, DRD4, NTSR1, NQO2, VWF, BDNF, EPHX1, AKR1C3, AKR1A1, MTHFR, GRIN2B, GFAP, ALDH1A1, CFTR	3.9	2.11E-05
C0011581~Depressive disorder	27	9.6	GABRB3, OXTR, ABCB1, NRXN1, CHAT, SLC1A1, ATP1A3, MTR, AQP4, CYP2C19, COMT, SLC6A4, RELN, CYP2D6, NOS1, DRD4, GSTM1, NOS2, NOS3, BDNF, MTHFR, MIF, GRIN2B, SYN1, GFAP, SLCO1C1, DLG4	3.7	2.25E-05
C0011570~Mental Depression	25	8.9	GABRB3, OXTR, ABCB1, NRXN1, CHAT, SLC1A1, ATP1A3, MTR, AQP4, CYP2C19, COMT, SLC6A4, RELN, CYP2D6, DRD4, GSTM1, NOS2, NOS3, BDNF, MTHFR, GRIN2B, SYN1, GFAP, SLCO1C1, DLG4	3.9	3.23E-05
C0030567~Parkinson Disease	14	5.0	NQO1, CNTNAP2, GSTM1, ABCB1, BDNF, HFE, GSTP1, HGF, INSR, MTHFR, CP, GFAP, CYP2D6, NOS1	6.6	2.90E-04
C1257931~Mammary Neoplasms, Human	35	12.5	NOTCH2, ACHE, NOTCH3, DNMT1, FLT1, NOTCH1, ABCB1, TFRC, GSTP1, NOTCH4, MTR, AHR, COMT, CYP19A1, PTGS1, LOXL2, HEY1, CYP2D6, DNMT3B, NQO1, BCHE, NQO2, GPX2, JAG1, NOS2, NOS3, DNMT3A, DIO3, MTHFR, MIF, CYP24A1, DPYD, CYP1A1, RARB, ABCG2	2.7	4.51E-04
C4704874~Mammary Carcinoma, Human	35	12.5	NOTCH2, ACHE, NOTCH3, DNMT1, FLT1, NOTCH1, ABCB1, TFRC, GSTP1, NOTCH4, MTR, AHR, COMT, CYP19A1, PTGS1, LOXL2, HEY1, CYP2D6, DNMT3B, NQO1, BCHE, NQO2, GPX2, JAG1, NOS2, NOS3, DNMT3A, DIO3, MTHFR, MIF, CYP24A1, DPYD, CYP1A1, RARB, ABCG2	2.7	4.51E-04
C1458155~Mammary Neoplasms	35	12.5	NOTCH2, ACHE, NOTCH3, DNMT1, FLT1, NOTCH1, ABCB1, TFRC, GSTP1, NOTCH4, MTR, AHR, COMT, CYP19A1, PTGS1, LOXL2, HEY1, CYP2D6, DNMT3B, NQO1, BCHE, NQO2, GPX2, JAG1, NOS2, NOS3, DNMT3A, DIO3, MTHFR, MIF, CYP24A1, DPYD, CYP1A1, RARB, ABCG2	2.7	4.97E-04
C0678222~Breast Carcinoma	35	12.5	NOTCH2, ACHE, NOTCH3, DNMT1, FLT1, NOTCH1, ABCB1, TFRC, GSTP1, NOTCH4, MTR, AHR, COMT, CYP19A1, PTGS1, LOXL2, HEY1, CYP2D6, DNMT3B, NQO1, BCHE, NQO2, GPX2, JAG1, NOS2, NOS3, DNMT3A, DIO3, MTHFR, MIF, CYP24A1, DPYD, CYP1A1, RARB, ABCG2	2.6	8.07E-04
C0400966~Non-alcoholic Fatty Liver Disease	12	4.3	ALDH4A1, NQO1, ADH4, GSTM1, ALDH2, ADH1B, GSTP1, ADH1A, CYP1A2, ALDH1A1, AHR, FOLR2	6.9	2.03E-03

C3241937~Nonalcoholic Steatohepatitis	12	4.3	ALDH4A1, NQO1, ADH4, GSTM1, ALDH2, ADH1B, GSTP1, ADH1A, CYP1A2, ALDH1A1, AHR, FOLR2	6.9	2.03E-03
C0751111~Awakening Epilepsy	12	4.3	GABRB3, CNTNAP2, MEF2C, ABCB1, BDNF, TXNRD1, ANKRD11, SLC1A1, CHD2, GRIN2B, SCN1A, GFAP	5.9	1.01E-02
C0236018~Aura	12	4.3	GABRB3, CNTNAP2, MEF2C, ABCB1, BDNF, TXNRD1, ANKRD11, SLC1A1, CHD2, GRIN2B, SCN1A, GFAP	5.9	1.01E-02
C0086237~Epilepsy, Cryptogenic	12	4.3	GABRB3, CNTNAP2, MEF2C, ABCB1, BDNF, TXNRD1, ANKRD11, SLC1A1, CHD2, GRIN2B, SCN1A, GFAP	5.9	1.01E-02
C0085762~Alcohol abuse	11	3.9	ADH4, ALDH2, BDNF, ADH1B, HFE, ALDH1A1, AKR1A1, COMT, MPDZ, DRD4, SLC6A4	6.6	1.02E-02
C0024121~Lung Neoplasms	21	7.5	NOTCH2, NOTCH3, GSTM1, PTGIS, JAG1, NOS2, TFRC, GSTP1, EPHX1, DNMT3A, MTHFR, SLC3A2, CYP24A1, APC, HEY1, CYP1A2, DPYD, RARB, SOX9, MET, ADA	3.2	1.79E-02
C0242379~Malignant neoplasm of lung	21	7.5	NOTCH2, NOTCH3, GSTM1, PTGIS, JAG1, NOS2, TFRC, GSTP1, EPHX1, DNMT3A, MTHFR, SLC3A2, CYP24A1, APC, HEY1, CYP1A2, DPYD, RARB, SOX9, MET, ADA	3.2	1.89E-02
C0006267~Bronchiectasis	7	2.5	SCNN1G, RSPH4A, DNAH11, DNAI2, DNAAF2, SCNN1A, CFTR	12.8	2.25E-02
C0033578~Prostatic Neoplasms	34	12.1	ACHE, DNMT1, GSTP1, ITGB3, ALOX12B, AHR, ADRB2, CYP2C19, COMT, PRKCZ, CYP3A5, CYP19A1, ALAD, HSD17B1, DNMT3B, AOX1, NQO1, CBR1, GSTM1, TXNRD2, GSTO1, NOS3, EPHX1, HSD3B1, AKR1C3, MTHFR, MIF, SULT2B1, CLDN3, APC, CYP11B2, PARD3, CYP1A1, MET	2.2	4.16E-02
C0376358~Malignant neoplasm of prostate	34	12.1	ACHE, DNMT1, GSTP1, ITGB3, ALOX12B, AHR, ADRB2, CYP2C19, COMT, PRKCZ, CYP3A5, CYP19A1, ALAD, HSD17B1, DNMT3B, AOX1, NQO1, CBR1, GSTM1, TXNRD2, GSTO1, NOS3, EPHX1, HSD3B1, AKR1C3, MTHFR, MIF, SULT2B1, CLDN3, APC, CYP11B2, PARD3, CYP1A1, MET	2.2	4.16E-02

S2.4.3 Significant KEGG Pathways for Pollutant-Disorder Gene Sets of Autism Spectrum Disorder

All significant KEGG Pathways are listed below, ranked by Bonferroni-corrected P-values.

Supplementary Table S38. KEGG Pathways for Autism Spectrum Disorder, Bonferroni-Corrected P-values < 0.05

Term	Count	%	Genes	Fold Enrichment	Bonferroni P-values
hsa00980:Metabolism of xenobiotics by cytochrome P450	34	12.1	UGT1A10, ADH1B, GSTP1, ADH1A, GSTT2, CYP3A5, ADH5, ADH6, ALDH3B2, ADH4, AKR7A2, AKR7A3, CYP2B6, CYP2D6, ALDH3B1, UGT2A3, CYP2F1, CBR3, UGT1A6, UGT2B10, CBR1, GSTM1, UGT1A1, GSTO1, EPHX1, CYP2C9, ALDH3A1, GSTA3, CYP2S1, GSTA2, CYP1A2, CYP1A1, UGT2B7, GSTM5	17.5	1.06E-30
hsa00982:Drug metabolism - cytochrome P450	33	11.8	UGT1A10, ADH1B, GSTP1, ADH1A, GSTT2, CYP2C19, CYP3A5, ADH5, ADH6, ALDH3B2, ADH4, CYP2B6, CYP2D6, ALDH3B1, UGT2A3, AOX1,	18.3	1.52E-30

			UGT1A6, UGT2B10, GSTM1, UGT1A1, GSTO1, FMO1, FMO2, FMO3, FMO4, CYP2C9, ALDH3A1, CYP2C8, GSTA3, GSTA2, CYP1A2, UGT2B7, GSTM5		
hsa01100:Metabolic pathways	105	37.5	UGT1A10, ALDH1L1, COMT, STS, UGT2B10, MIF, CYP27A1, SULT2B1, ALDH3A2, ALDH3A1, CYP2U1, PTGES, DNMT1, ALOX15, GSTT2, ALOX12, CYP2C19, CYP19A1, ADH5, ADH6, CYP27B1, ADH4, ALDH3B2, CYP2B6, CYP11A1, ALDH3B1, CHST10, CYP2J2, GSTM1, EPHX2, MTHFR, DHCR24, CYP2C9, CYP2C8, ALDH6A1, AKR1B10, CYP11B2, CYP2S1, PAH, CYP1A2, CYP1A1, ADA, GSTM5, GAL3ST1, MTR, ALAD, CYP26B1, ALDH2, CA2, CYP2R1, DNMT3B, AOX1, UGT2A3, UPP2, NOS1, GUSB, CHST8, CBR1, GPX2, ADSL, PTGIS, UGT1A1, GSTO1, GPX6, GPX5, DNMT3A, AKR1A1, ALDH1A3, ALDH5A1, CYP21A2, DPYD, ALDH1A1, UGT2B7, ADH1B, ADH1A, GSTP1, ALOX12B, CYP3A5, CYP7A1, CYP3A7, PTGS1, CBS, HSD17B1, UGT1A6, CBR3, NQO1, AOC1, AOC2, NOS2, PTGES2, NOS3, B3GAT2, HSD3B1, AKR1C3, FMO1, AKR1C2, FMO2, FMO3, FMO4, SUOX, GALNS, ALDH4A1, CYP24A1, GSTA3, GSTA2	2.7	5.96E-23
hsa00830:Retinol metabolism	24	8.6	UGT2B10, UGT1A10, UGT1A1, ADH1B, ADH1A, CYP3A5, ADH5, ADH6, CYP3A7, CYP2C9, ALDH1A3, ADH4, CYP27C1, CYP2C8, CYP26B1, CYP2B6, CYP2S1, CYP1A2, ALDH1A1, CYP1A1, UGT2A3, AOX1, UGT2B7, UGT1A6	14.3	2.09E-18
hsa00140:Steroid hormone biosynthesis	22	7.9	UGT2B10, UGT1A10, UGT1A1, HSD3B1, AKR1C3, AKR1C2, COMT, CYP3A5, CYP7A1, CYP19A1, CYP3A7, SULT2B1, STS, CYP11A1, CYP11B2, CYP21A2, HSD17B1, CYP1A2, CYP1A1, UGT2A3, UGT2B7, UGT1A6	14.4	1.06E-16
hsa05204:Chemical carcinogenesis - DNA adducts	23	8.2	UGT2B10, CBR1, UGT1A10, GSTM1, UGT1A1, GSTO1, GSTP1, EPHX1, GSTT2, AKR1C2, CYP2C19, CYP3A5, CYP3A7, CYP2C9, CYP2C8, GSTA3, GSTA2, CYP1A2, CYP1A1, UGT2A3, UGT2B7, GSTM5, UGT1A6	13.1	1.33E-16
hsa00590:Arachidonic acid metabolism	18	6.4	CYP2J2, CBR1, PTGIS, PTGES2, EPHX2, ALOX15, AKR1C3, ALOX12, ALOX12B, PTGR1, CYP2C19, PTGS1, CYP2C9, CYP2C8, CYP2B6, CYP2U1, PTGES, CBR3	12.0	1.17E-11
hsa00983:Drug metabolism - other enzymes	18	6.4	UGT2B10, UGT1A10, GSTM1, UGT1A1, TPMT, GSTO1, GSTP1, GSTT2, GSTA3, GSTA2, DPYD, UGT2A3, UPP2, GUSB, UGT2B7, CES2, GSTM5, UGT1A6	9.0	1.81E-09
hsa04976:Bile secretion	17	6.1	UGT2B10, ABCC3, UGT1A10, ABCB1, UGT1A1, EPHX1, AQP9, ATP1A3, AQP4, ATP1B2, CYP7A1, CA2, UGT2A3, UGT2B7, CFTR, UGT1A6, ABCG2	7.7	1.07E-07
hsa00350:Tyrosine metabolism	12	4.3	ALDH3A1, ALDH3B2, ADH4, AOC2, ADH1B, ADH1A, ALDH3B1, AOX1, MIF, COMT, ADH5, ADH6	13.5	1.32E-07
hsa00053:Ascorbate and aldarate metabolism	10	3.6	ALDH3A2, UGT2B10, UGT1A10, UGT1A1, ALDH2, AKR1A1, UGT2A3, GUSB, UGT2B7, UGT1A6	13.5	6.22E-06
hsa05207:Chemical carcinogenesis - receptor activation	21	7.5	UGT2B10, NOTCH2, UGT1A10, GSTM1, JAG1, UGT1A1, GSTO1, EPHX2, EPHX1, GSTT2, AHR, ADRB2, CYP2B6, GSTA3, GSTA2, CYP1A2, CYP1A1, UGT2A3, UGT2B7, GSTM5, UGT1A6	4.0	7.09E-05
hsa00040:Pentose and glucuronate interconversions	9	3.2	UGT2B10, UGT1A10, AKR1B10, UGT1A1, AKR1A1, UGT2A3, GUSB, UGT2B7, UGT1A6	10.1	4.81E-04

hsa04726:Serotonergic synapse	14	5.0	GABRB3, CYP2J2, ALOX15, ALOX12, ALOX12B, ITPR3, CYP2C19, SLC6A4, PTGS1, GNGT1, CYP2C9, CYP2C8, CYP4X1, CYP2D6	4.9	1.09E-03
hsa00010:Glycolysis / Gluconeogenesis	11	3.9	ALDH3A2, ALDH3A1, ALDH3B2, ADH4, ALDH2, ADH1B, ADH1A, ALDH3B1, AKR1A1, ADH5, ADH6	6.7	1.17E-03
hsa00410:beta-Alanine metabolism	8	2.9	ALDH3A2, ALDH3A1, ALDH3B2, ALDH6A1, AOC2, ALDH2, ALDH3B1, DPYD	10.5	1.92E-03
hsa00480:Glutathione metabolism	10	3.6	GPX2, GSTM1, GSTO1, GSTA3, GPX6, GSTP1, GSTA2, GPX5, GSTT2, GSTM5	7.0	2.54E-03
hsa05208:Chemical carcinogenesis - reactive oxygen species	19	6.8	NQO1, CBR1, GSTM1, GSTO1, EPHX2, HGF, EPHX1, AKR1C3, AKR1A1, GSTT2, AKR1C2, AHR, GSTA3, GSTA2, CYP1A2, CYP1A1, CYP2F1, MET, GSTM5	3.4	2.59E-03
hsa00860:Porphyrin metabolism	9	3.2	ALAD, UGT2B10, UGT1A10, UGT1A1, UGT2A3, GUSB, CP, UGT2B7, UGT1A6	7.9	3.35E-03
hsa00360:Phenylalanine metabolism	6	2.1	ALDH3A1, ALDH3B2, AOC2, ALDH3B1, PAH, MIF	15.2	7.37E-03
hsa00071:Fatty acid degradation	8	2.9	ALDH3A2, ADH4, ALDH2, CYP2U1, ADH1B, ADH1A, ADH5, ADH6	7.5	1.82E-02
hsa00620:Pyruvate metabolism	8	2.9	ALDH3A2, ADH4, ALDH2, ADH1B, ADH1A, AKR1A1, ADH5, ADH6	6.9	3.24E-02
hsa00340:Histidine metabolism	6	2.1	ALDH3A2, ALDH3A1, ALDH3B2, AOC1, ALDH2, ALDH3B1	11.1	3.88E-02

S2.5 Functional Annotation for Pollutant-Disorder Gene Sets of Intellectual Disability

S2.5.1 Significant GO Biological Processes for Pollutant-Disorder Gene Sets of Intellectual Disability

Gene Ontology (GO) terms consist of three categories: biological process (BP), cellular component (CC), and molecular function (MF). In this study, we focused on the biological functions of shared genes, so GO term annotation was primarily conducted using the BP_DIRECT category for biological annotation. All significant GO Biological Processes are listed below. If fewer than five significant GO Biological Processes are identified, the top five Biological Processes ranked by Bonferroni-corrected P-values will be presented.

Supplementary Table S39. Air Pollutants (MeSH: D000393) to Intellectual Disability (MeSH: D008607), Bonferroni-Corrected P-values < 0.05

Term	Count	%	Genes	Fold Enrichment	Bonferroni P-values
GO:0035176~social behavior	5	15.2	MECP2, TH, NRXN1, CIC, SHANK3	54.4	1.17E-03
GO:0043524~negative regulation of neuron apoptotic process	5	15.2	MECP2, PRKCG, MEF2C, CHL1, BDNF	19.8	6.22E-02
GO:0007612~learning	4	12.1	TH, NRXN1, CIC, SHANK3	40.5	7.31E-02
GO:0007416~synapse assembly	4	12.1	MECP2, BDNF, NRXN1, SHANK3	35.7	1.05E-01
GO:0021987~cerebral cortex development	4	12.1	MECP2, TH, KCNA2, SLC2A1	30.7	1.60E-01

Supplementary Table S40. Particulate Matter (MeSH: D052638) to Intellectual Disability (MeSH: D008607), Bonferroni-Corrected P-values < 0.05

Term	Count	%	Genes	Fold Enrichment	Bonferroni P-values
GO:0007416~synapse assembly	5	25.0	MECP2, BDNF, NRXN1, PTEN, SHANK3	73.6	2.96E-04
GO:0030534~adult behavior	4	20.0	NAGLU, NRXN1, PTEN, SHANK3	123.3	2.41E-03
GO:0035176~social behavior	4	20.0	MECP2, NRXN1, PTEN, SHANK3	71.8	1.25E-02
GO:0043524~negative regulation of neuron apoptotic process	4	20.0	MECP2, MEF2C, CHL1, BDNF	26.1	2.25E-01
GO:2000463~positive regulation of excitatory postsynaptic potential	3	15.0	NRXN1, PTEN, SHANK3	92.5	2.44E-01

Supplementary Table S41. Lead (MeSH: D007854) to Intellectual Disability (MeSH: D008607), Bonferroni-Corrected P-values < 0.05

Term	Count	%	Genes	Fold Enrichment	Bonferroni P-values

GO:0035176~social behavior	6	13.6	MECP2, PTCHD1, TH, NRXN1, PTEN, SHANK2	50.1	7.61E-05
GO:0007416~synapse assembly	5	11.4	MECP2, BDNF, NRXN1, PTEN, SHANK2	34.2	8.50E-03
GO:0043524~negative regulation of neuron apoptotic process	5	11.4	MECP2, PRKCG, MEF2C, CHL1, BDNF	15.2	1.84E-01
GO:0060291~long-term synaptic potentiation	4	9.1	MECP2, PRKCG, GRIN2B, SHANK2	27.0	2.46E-01
GO:0007611~learning or memory	4	9.1	PRKCG, MEF2C, PTEN, GRIN2B	27.0	2.46E-01

Supplementary Table S42. Zinc (MeSH: D015032) to Intellectual Disability (MeSH: D008607), Bonferroni-Corrected P-values < 0.05

Term	Count	%	Genes	Fold Enrichment	Bonferroni P-values
GO:0035176~social behavior	5	17.2	TH, PTEN, CIC, SHANK3, SHANK2	64.1	5.83E-04
GO:0060291~long-term synaptic potentiation	5	17.2	PRKCG, NF1, GRIN2B, SHANK3, SHANK2	51.8	1.38E-03
GO:0007612~learning	4	13.8	TH, CIC, SHANK3, SHANK2	47.8	4.46E-02
GO:0007416~synapse assembly	4	13.8	BDNF, PTEN, SHANK3, SHANK2	42.1	6.45E-02
GO:0007399~nervous system development	6	20.7	HDAC4, APC, BDNF, FMR1, PTEN, PAX6	10.2	1.32E-01

Supplementary Table S43. Glyphosate (MeSH: D000097797) to Intellectual Disability (MeSH: D008607), Bonferroni-Corrected P-values < 0.05

Term	Count	%	Genes	Fold Enrichment	Bonferroni P-values
GO:0035176~social behavior	4	13.8	TH, CHD8, NRXN1, SHANK3	49.5	3.55E-02
GO:0007613~memory	4	13.8	NTF4, TH, BDNF, SHANK3	31.3	1.32E-01
GO:0006338~chromatin remodeling	5	17.2	KDM5A, HDAC4, CHD8, NSD1, PAX6	9.9	5.27E-01
GO:0032259~methylation	4	13.8	GAMT, KDM5A, NSD1, DNMT3A	17.0	5.67E-01
GO:0000122~negative regulation of transcription by RNA polymerase II	7	24.1	KDM5A, HDAC4, PARP1, CHD8, NSD1, DNMT3A, PAX6	4.9	6.76E-01

Supplementary Table S44. Perfluorooctane Sulfonic Acid (MeSH: C076994) to Intellectual Disability (MeSH: D008607), Bonferroni-Corrected P-values < 0.05

Term	Count	%	Genes	Fold Enrichment	Bonferroni P-values
GO:0048667~cell morphogenesis involved in neuron differentiation	3	8.1	MEF2C, HEXA, TBCD	160.0	8.64E-02

GO:0043524~negative regulation of neuron apoptotic process	4	10.8	MECP2, PRKCG, MEF2C, BDNF	14.1	8.05E-01
GO:0032259~methylation	4	10.8	METTL23, KDM5C, DNMT3A, COQ5	13.3	8.54E-01
GO:0035176~social behavior	3	8.1	MECP2, TH, CIC	29.1	9.41E-01
GO:0050890~cognition	3	8.1	METTL23, GNAS, MFSD2A	29.1	9.41E-01

Supplementary Table S45. Perfluorohexanesulfonic Acid (MeSH: C471071) to Intellectual Disability (MeSH: D008607), Bonferroni-Corrected P-values < 0.05

Term	Count	%	Genes	Fold Enrichment	Bonferroni P-values
GO:0001501~skeletal system development	3	14.3	HDAC4, RAI1, HEXA	22.8	9.09E-01
GO:0060445~branching involved in salivary gland morphogenesis	2	9.5	LAMA1, FGFR2	179.4	9.75E-01
GO:0040015~negative regulation of multicellular organism growth	2	9.5	RAI1, GNAS	151.8	9.87E-01
GO:0050884~neuromuscular process controlling posture	2	9.5	HEXA, SCN1A	141.0	9.91E-01
GO:0010832~negative regulation of myotube differentiation	2	9.5	HDAC4, BDNF	123.3	9.95E-01

Supplementary Table S46. Polychlorinated Biphenyls (MeSH: D011078) to Intellectual Disability (MeSH: D008607), Bonferroni-Corrected P-values < 0.05

Term	Count	%	Genes	Fold Enrichment	Bonferroni P-values
GO:0045471~response to ethanol	3	11.5	TH, GRIN2B, FGFR2	21.1	9.87E-01
GO:0001764~neuron migration	3	11.5	MEF2C, DISC1, FGFR2	19.3	9.94E-01
GO:0021860~pyramidal neuron development	2	7.7	SLC4A10, FGFR2	168.7	9.97E-01
GO:0009887~animal organ morphogenesis	3	11.5	COL18A1, TH, FGFR2	17.0	9.99E-01
GO:0071456~cellular response to hypoxia	3	11.5	CCNA2, DNMT3A, FGFR2	16.0	9.99E-01

Supplementary Table S47. 2,4,5,2',4',5'-hexachlorobiphenyl (MeSH: C014024) to Intellectual Disability (MeSH: D008607), Bonferroni-Corrected P-values < 0.05

Term	Count	%	Genes	Fold Enrichment	Bonferroni P-values
GO:0035176~social behavior	3	12.5	TH, PTEN, SHANK2	44.8	5.37E-01
GO:0040015~negative regulation of multicellular organism growth	2	8.3	RAI1, GNAS	126.5	9.98E-01

GO:0009416~response to light stimulus	2	8.3	TH, SLC4A10	86.6	1.00E+00
GO:0034244~negative regulation of transcription elongation by RNA polymerase II	2	8.3	PARP1, LARP7	82.2	1.00E+00
GO:0007507~heart development	3	12.5	KDM6B, TH, PTEN	11.6	1.00E+00

S2.5.2 Significant DisGeNET Associations for Pollutant-Disorder Gene Sets of Intellectual Disability

All significant DisGeNET Associations are listed below, ranked by Bonferroni-corrected P-values.

Supplementary Table S48. DisGeNET Associations for Intellectual Disability, Bonferroni-Corrected P-values < 0.05

Term	Count	%	Genes	Fold Enrichment	Bonferroni P-values
C0020796~Profound Mental Retardation	98	100.0	HYCC1, FMR1, FRY, TSEN54, YY1, EEF1B2, SYNGAP1, NAGLU, H4C3, SCAPER, RGS7, MFSD2A, SCN1A, GAMT, PRKCG, PDHX, CCBE1, MEF2C, ENTPD1, INPP4A, SCN8A, PHIP, RABL6, CALCA, WDR62, CACNA1G, RAI1, SRGAP3, RALGDS, ARL14EP, BDNF, SLC4A10, TBCD, LARP7, MCC, GRIN2B, COQ5, MED13L, GON4L, TH, FASN, NF1, GNAS, FGFR2, KDM5A, COL18A1, KDM5C, DOCK8, CHD8, HEXA, PTEN, SLC2A1, ADK, AP4E1, MECP2, CHL1, CASP2, BBS7, CA8, CIC, KDM6B, PARP1, DCC, DNMT3A, PAX6, KIF7, ADRA2B, METTL23, CCNA2, ALDH5A1, SETBP1, PEX6, L2HGDH, SHANK3, SHANK2, HDAC4, LAMA1, WDR45B, NRXN1, KCNA2, ZBTB40, NTF4, PTCHD1, NSD1, SC5D, DISC1, DYNC1H1, SLC31A1, TMCO1, LETM1, H3-4, APC, POLR3B, CAPN10, PRKRA, NAA15, FOLR1, TAF2	69.8	2.59E-196
C0917816~Mental deficiency	98	100.0	HYCC1, FMR1, FRY, TSEN54, YY1, EEF1B2, SYNGAP1, NAGLU, H4C3, SCAPER, RGS7, MFSD2A, SCN1A, GAMT, PRKCG, PDHX, CCBE1, MEF2C, ENTPD1, INPP4A, SCN8A, PHIP, RABL6, CALCA, WDR62, CACNA1G, RAI1, SRGAP3, RALGDS, ARL14EP, BDNF, SLC4A10, TBCD, LARP7, MCC, GRIN2B, COQ5, MED13L, GON4L, TH, FASN, NF1, GNAS, FGFR2, KDM5A, COL18A1, KDM5C, DOCK8, CHD8, HEXA, PTEN, SLC2A1, ADK, AP4E1, MECP2, CHL1, CASP2, BBS7, CA8, CIC, KDM6B, PARP1, DCC, DNMT3A, PAX6, KIF7, ADRA2B, METTL23, CCNA2, ALDH5A1, SETBP1, PEX6, L2HGDH, SHANK3, SHANK2, HDAC4, LAMA1, WDR45B, NRXN1, KCNA2, ZBTB40, NTF4, PTCHD1, NSD1, SC5D, DISC1, DYNC1H1, SLC31A1, TMCO1, LETM1, H3-4, APC, POLR3B, CAPN10, PRKRA, NAA15, FOLR1, TAF2	69.8	2.59E-196
C0025363~Mental Retardation, Psychosocial	98	100.0	HYCC1, FMR1, FRY, TSEN54, YY1, EEF1B2, SYNGAP1, NAGLU, H4C3, SCAPER, RGS7, MFSD2A, SCN1A, GAMT, PRKCG, PDHX, CCBE1, MEF2C, ENTPD1, INPP4A, SCN8A, PHIP, RABL6, CALCA, WDR62, CACNA1G,	69.8	2.59E-196

			RAI1, SRGAP3, RALGDS, ARL14EP, BDNF, SLC4A10, TBCD, LARP7, MCC, GRIN2B, COQ5, MED13L, GON4L, TH, FASN, NF1, GNAS, FGFR2, KDM5A, COL18A1, KDM5C, DOCK8, CHD8, HEXA, PTEN, SLC2A1, ADK, AP4E1, MECP2, CHL1, CASP2, BBS7, CA8, CIC, KDM6B, PARP1, DCC, DNMT3A, PAX6, KIF7, ADRA2B, METTL23, CCNA2, ALDH5A1, SETBP1, PEX6, L2HGDH, SHANK3, SHANK2, HDAC4, LAMA1, WDR45B, NRXN1, KCNA2, ZBTB40, NTF4, PTCHD1, NSD1, SC5D, DISC1, DYNC1H1, SLC31A1, TMCO1, LETM1, H3-4, APC, POLR3B, CAPN10, PRKRA, NAA15, FOLR1, TAF2		
C3714756~Intellectual Disability	98	100.0	HYCC1, FMR1, FRY, TSEN54, YY1, EEF1B2, SYNGAP1, NAGLU, H4C3, SCAPER, RGS7, MFSD2A, SCN1A, GAMT, PRKCG, PDHX, CCBE1, MEF2C, ENTPD1, INPP4A, SCN8A, PHIP, RABL6, CALCA, WDR62, CACNA1G, RAI1, SRGAP3, RALGDS, ARL14EP, BDNF, SLC4A10, TBCD, LARP7, MCC, GRIN2B, COQ5, MED13L, GON4L, TH, FASN, NF1, GNAS, FGFR2, KDM5A, COL18A1, KDM5C, DOCK8, CHD8, HEXA, PTEN, SLC2A1, ADK, AP4E1, MECP2, CHL1, CASP2, BBS7, CA8, CIC, KDM6B, PARP1, DCC, DNMT3A, PAX6, KIF7, ADRA2B, METTL23, CCNA2, ALDH5A1, SETBP1, PEX6, L2HGDH, SHANK3, SHANK2, HDAC4, LAMA1, WDR45B, NRXN1, KCNA2, ZBTB40, NTF4, PTCHD1, NSD1, SC5D, DISC1, DYNC1H1, SLC31A1, TMCO1, LETM1, H3-4, APC, POLR3B, CAPN10, PRKRA, NAA15, FOLR1, TAF2	21.7	5.86E-132
C0004352~Autistic Disorder	21	21.4	MEF2C, KDM5C, CALCA, BDNF, NRXN1, CHD8, FMR1, PTEN, PAX6, MCC, GRIN2B, MECP2, NTF4, SYNGAP1, APC, PTCHD1, NSD1, NF1, DISC1, SHANK3, SHANK2	8.0	9.89E-10
C0376634~Craniofacial Abnormalities	14	14.3	NRXN1, FMR1, DNMT3A, PTEN, PAX6, TMCO1, MECP2, LETM1, RAI1, SETBP1, PRKRA, FOLR1, H4C3, FGFR2	8.9	5.12E-06
C1510586~Autism Spectrum Disorders	11	11.2	RAI1, MEF2C, PTCHD1, BDNF, CHD8, NRXN1, DNMT3A, CIC, GRIN2B, SHANK3, SCN1A	12.8	1.27E-05
C3853041~Severe Congenital Microcephaly	8	8.2	DYNC1H1, DNMT3A, SLC2A1, TBCD, WDR62, H4C3, TSEN54, MFSD2A	25.5	2.10E-05
C1535926~Neurodevelopmental Disorders	11	11.2	MECP2, MEF2C, SYNGAP1, SETBP1, DOCK8, SCN8A, PTEN, SRGAP3, GRIN2B, SCN1A, MED13L	11.7	3.07E-05
C1956147~Microlissencephaly	8	8.2	DYNC1H1, DNMT3A, SLC2A1, TBCD, WDR62, H4C3, TSEN54, MFSD2A	24.0	3.35E-05
C0751111~Awakening Epilepsy	10	10.2	MECP2, MEF2C, SYNGAP1, BDNF, SCN8A, SLC4A10, L2HGDH, GRIN2B, FOLR1, SCN1A	12.1	1.33E-04
C0236018~Aura	10	10.2	MECP2, MEF2C, SYNGAP1, BDNF, SCN8A, SLC4A10, L2HGDH, GRIN2B, FOLR1, SCN1A	12.1	1.33E-04
C0086237~Epilepsy, Cryptogenic	10	10.2	MECP2, MEF2C, SYNGAP1, BDNF, SCN8A, SLC4A10, L2HGDH, GRIN2B, FOLR1, SCN1A	12.1	1.33E-04
C0014544~Epilepsy	11	11.2	MECP2, MEF2C, KDM5C, SYNGAP1, BDNF, SCN8A, SLC4A10, L2HGDH, GRIN2B, FOLR1, SCN1A	10.0	1.42E-04
C0751257~Auditory Processing Disorder, Central	5	5.1	SETBP1, NRXN1, KCNA2, PTEN, SHANK3	45.0	3.54E-03

C0023014~Language Development Disorders	5	5.1	SETBP1, NRXN1, KCNA2, PTEN, SHANK3	45.0	3.54E-03
C0454655~Semantic-Pragmatic Disorder	5	5.1	SETBP1, NRXN1, KCNA2, PTEN, SHANK3	45.0	3.54E-03
C0023012~Language Delay	5	5.1	SETBP1, NRXN1, KCNA2, PTEN, SHANK3	45.0	3.54E-03
C0241210~Speech Delay	5	5.1	SETBP1, NRXN1, KCNA2, PTEN, SHANK3	45.0	3.54E-03
C0025958~Microcephaly	8	8.2	DYNC1H1, DNMT3A, SLC2A1, TBCD, WDR62, H4C3, TSEN54, MFSD2A	11.8	5.16E-03
C0085997~Child Development Disorders, Specific	6	6.1	MECP2, DOCK8, PTEN, SLC2A1, TBCD, SHANK3	20.5	1.06E-02
C0085996~Child Development Deviations	6	6.1	MECP2, DOCK8, PTEN, SLC2A1, TBCD, SHANK3	20.5	1.06E-02
C0008073~Developmental Disabilities	6	6.1	MECP2, DOCK8, PTEN, SLC2A1, TBCD, SHANK3	19.8	1.26E-02

S2.5.3 Significant KEGG Pathways for Pollutant-Disorder Gene Sets of Intellectual Disability

No significant KEGG Pathways were identified. Therefore, the top ten KEGG Pathways ranked by Bonferroni-corrected P-values are listed below.

Supplementary Table S49. KEGG Pathways for Intellectual Disability

Term	Count	%	Genes	Fold Enrichment	Bonferroni P-values
hsa04014:Ras signaling pathway	8	8.2	NTF4, PRKCG, SYNGAP1, BDNF, NF1, GRIN2B, RALGDS, FGFR2	4.9	1.84E-01
hsa05034:Alcoholism	7	7.1	HDAC4, H3-4, TH, BDNF, GNAS, GRIN2B, H4C3	5.4	2.68E-01
hsa05200:Pathways in cancer	11	11.2	CCNA2, PRKCG, APC, LAMA1, DCC, PTEN, SLC2A1, GNAS, KIF7, RALGDS, FGFR2	3.0	4.18E-01
hsa05030:Cocaine addiction	4	4.1	TH, BDNF, GNAS, GRIN2B	11.8	5.64E-01
hsa04724:Glutamatergic synapse	5	5.1	PRKCG, GNAS, GRIN2B, SHANK3, SHANK2	6.3	7.62E-01
hsa05031:Amphetamine addiction	4	4.1	PRKCG, TH, GNAS, GRIN2B	8.4	8.84E-01
hsa04728:Dopaminergic synapse	5	5.1	PRKCG, TH, GNAS, GRIN2B, SCN1A	5.5	9.01E-01
hsa04010:MAPK signaling pathway	7	7.1	NTF4, PRKCG, MEF2C, BDNF, NF1, FGFR2, CACNA1G	3.4	9.49E-01
hsa01521:EGFR tyrosine kinase inhibitor resistance	4	4.1	PRKCG, PTEN, NF1, FGFR2	7.2	9.60E-01
hsa04713:Circadian entrainment	4	4.1	PRKCG, GNAS, GRIN2B, CACNA1G	6.0	9.95E-01

S2.6 Temporal-Specific Expression Signatures

By fitting a multivariable linear model for each gene (expression ~ age + sex + brain region), we obtained t-values and adjusted P-values for 18,911 genes. The genes were ranked based on their t-values, and the top 5% with the highest expression levels and the bottom 5% with the lowest expression levels (adjusted P-values < 0.05) were selected. This resulted in two gene expression groups (946 genes * 2 groups) from 560 samples, covering the entire human brain development cycle, including early prenatal, intermediate prenatal, late prenatal, early postnatal, intermediate postnatal, and late postnatal stages. These gene groups were subsequently applied in further analyses of pollutant-disorder gene sets associated with autism spectrum disorder and intellectual disability.

S2.6.1 Temporal Expression of Pollutant-Disorder Gene Sets for Autism Spectrum Disorder

The gene expression matrix was filtered based on the mapping of autism spectrum disorder pollutant-disorder gene sets to the BrainSpan gene expression data. Five genes—"AFDN," "GRIN2B," "GSTT2," "GTF2I," and "ODAD2"—were excluded due to their absence in the expression data, narrowing the analysis to 275 genes.

Fisher's exact test was used to assess the association between the autism spectrum disorder gene sets and temporal-specific expression groups, utilizing 2×2 contingency tables. Odds ratios and adjusted P-values (Bonferroni corrected for 25 multiple time comparisons) were calculated to describe the associations.

2×2 Contingency Tables	ASD Gene	Not ASD Gene
High/Low Expressed Gene	A	B
Not High/Low Expressed Gene	C	D

Supplementary Table S50. Associations Between Autism Spectrum Disorder Gene Sets and High Expressed Gene Groups

Time Point	All Pollutants		Air Pollutants		Toxic and Trace Elements		Pesticides and Related Compounds		Synthetic Organic Chemicals	
	Odds Ratio	P-Adjusted	Odds Ratio	P-Adjusted	Odds Ratio	P-Adjusted	Odds Ratio	P-Adjusted	Odds Ratio	P-Adjusted
12 pcw	0.28	7.82E-02	0.15	5.63E-01	0.11	9.09E-02	0.23	1.00E+00	0.20	1.73E-01
13 pcw	0.79	1.00E+00	0.77	1.00E+00	0.70	1.00E+00	0.72	1.00E+00	0.50	1.00E+00
16 pcw	0.57	1.00E+00	0.77	1.00E+00	0.46	1.00E+00	0.97	1.00E+00	0.60	1.00E+00
17 pcw	0.71	1.00E+00	0.93	1.00E+00	0.82	1.00E+00	0.72	1.00E+00	0.92	1.00E+00
19 pcw	0.35	2.81E-01	0.30	1.00E+00	0.46	1.00E+00	0.23	1.00E+00	0.50	1.00E+00
21 pcw	0.94	1.00E+00	1.44	1.00E+00	1.60	1.00E+00	0.72	1.00E+00	1.03	1.00E+00
24 pcw	0.71	1.00E+00	0.61	1.00E+00	0.95	1.00E+00	0.47	1.00E+00	0.82	1.00E+00

37 pcw	1.92	1.16E-01	2.16	3.30E-01	1.33	1.00E+00	1.50	1.00E+00	2.08	1.13E-01
4 mos	2.19	9.34E-03	2.36	1.54E-01	3.07	2.72E-04	2.97	6.21E-02	1.84	7.19E-01
10 mos	0.71	1.00E+00	0.93	1.00E+00	0.70	1.00E+00	0.47	1.00E+00	0.50	1.00E+00
1 yrs	1.34	1.00E+00	1.44	1.00E+00	1.47	1.00E+00	1.50	1.00E+00	1.14	1.00E+00
2 yrs	1.67	8.67E-01	1.80	1.00E+00	1.88	7.52E-01	0.47	1.00E+00	1.60	1.00E+00
3 yrs	2.93	3.17E-06	3.15	1.70E-03	3.38	2.48E-05	2.35	5.27E-01	3.56	8.70E-07
4 yrs	0.21	1.82E-02	0.30	1.00E+00	0.11	9.09E-02	0.23	1.00E+00	0.30	4.93E-01
8 yrs	1.42	1.00E+00	2.16	3.30E-01	1.33	1.00E+00	1.23	1.00E+00	1.60	1.00E+00
11 yrs	0.35	2.81E-01	0.61	1.00E+00	0.34	1.00E+00	0.47	1.00E+00	0.50	1.00E+00
13 yrs	1.75	4.15E-01	2.16	3.30E-01	1.88	7.52E-01	1.78	1.00E+00	1.48	1.00E+00
18 yrs	2.28	5.23E-03	2.55	4.60E-02	2.45	2.33E-02	1.78	1.00E+00	2.59	3.88E-03
19 yrs	0.79	1.00E+00	0.61	1.00E+00	0.82	1.00E+00	0.72	1.00E+00	0.60	1.00E+00
21 yrs	0.64	1.00E+00	0.45	1.00E+00	0.58	1.00E+00	0.23	1.00E+00	0.60	1.00E+00
23 yrs	2.01	4.65E-02	2.74	1.64E-02	1.88	7.52E-01	1.78	1.00E+00	2.21	5.69E-02
30 yrs	1.02	1.00E+00	0.61	1.00E+00	0.70	1.00E+00	0.72	1.00E+00	0.92	1.00E+00
36 yrs	0.94	1.00E+00	0.45	1.00E+00	1.08	1.00E+00	0.72	1.00E+00	1.03	1.00E+00
37 yrs	0.94	1.00E+00	0.45	1.00E+00	0.70	1.00E+00	0.47	1.00E+00	1.03	1.00E+00
40 yrs	0.71	1.00E+00	0.77	1.00E+00	0.82	1.00E+00	1.23	1.00E+00	0.60	1.00E+00

Supplementary Table S50. Associations Between Autism Spectrum Disorder Gene Sets and Low Expressed Gene Groups

Time Point	All Pollutants		Air Pollutants		Toxic and Trace Elements		Pesticides and Related Compounds		Synthetic Organic Chemicals	
	Odds Ratio	P-Adjusted	Odds Ratio	P-Adjusted	Odds Ratio	P-Adjusted	Odds Ratio	P-Adjusted	Odds Ratio	P-Adjusted
12 pcw	0.94	1.00E+00	0.77	1.00E+00	1.20	1.00E+00	0.72	1.00E+00	0.71	1.00E+00
13 pcw	1.50	1.00E+00	1.80	1.00E+00	1.60	1.00E+00	1.78	1.00E+00	1.26	1.00E+00
16 pcw	2.37	1.57E-03	2.94	5.45E-03	2.60	1.15E-02	1.50	1.00E+00	2.08	1.13E-01
17 pcw	1.10	1.00E+00	1.62	1.00E+00	1.20	1.00E+00	0.72	1.00E+00	1.26	1.00E+00
19 pcw	0.71	1.00E+00	1.10	1.00E+00	0.70	1.00E+00	0.47	1.00E+00	0.71	1.00E+00

21 pcw	2.10	2.73E-02	2.35	1.55E-01	2.02	2.98E-01	1.23	1.00E+00	2.34	1.95E-02
24 pcw	1.58	1.00E+00	1.62	1.00E+00	1.60	1.00E+00	0.47	1.00E+00	1.60	1.00E+00
37 pcw	0.42	6.34E-01	0.45	1.00E+00	0.34	1.00E+00	0.47	1.00E+00	0.50	1.00E+00
4 mos	0.64	1.00E+00	0.61	1.00E+00	0.70	1.00E+00	0.47	1.00E+00	0.40	1.00E+00
10 mos	0.42	6.34E-01	0.15	5.63E-01	0.70	1.00E+00	0.00	8.96E-01	0.30	4.93E-01
1 yrs	0.64	1.00E+00	0.93	1.00E+00	0.82	1.00E+00	0.72	1.00E+00	0.82	1.00E+00
2 yrs	0.21	1.82E-02	0.15	5.63E-01	0.23	4.70E-01	0.00	8.96E-01	0.10	3.11E-02
3 yrs	0.42	6.34E-01	0.45	1.00E+00	0.34	1.00E+00	0.47	1.00E+00	0.10	3.11E-02
4 yrs	1.02	1.00E+00	1.10	1.00E+00	1.33	1.00E+00	0.47	1.00E+00	1.03	1.00E+00
8 yrs	0.94	1.00E+00	0.61	1.00E+00	0.82	1.00E+00	0.97	1.00E+00	0.82	1.00E+00
11 yrs	0.57	1.00E+00	0.61	1.00E+00	0.58	1.00E+00	0.23	1.00E+00	0.71	1.00E+00
13 yrs	0.28	7.82E-02	0.00	8.04E-02	0.34	1.00E+00	0.23	1.00E+00	0.30	4.93E-01
18 yrs	0.49	1.00E+00	0.30	1.00E+00	0.58	1.00E+00	0.72	1.00E+00	0.40	1.00E+00
19 yrs	0.49	1.00E+00	0.61	1.00E+00	0.58	1.00E+00	0.47	1.00E+00	0.60	1.00E+00
21 yrs	0.49	1.00E+00	0.61	1.00E+00	0.58	1.00E+00	0.72	1.00E+00	0.50	1.00E+00
23 yrs	0.49	1.00E+00	0.61	1.00E+00	0.58	1.00E+00	0.47	1.00E+00	0.60	1.00E+00
30 yrs	0.28	7.82E-02	0.45	1.00E+00	0.23	4.70E-01	0.23	1.00E+00	0.30	4.93E-01
36 yrs	0.35	2.81E-01	0.15	5.63E-01	0.34	1.00E+00	0.47	1.00E+00	0.50	1.00E+00
37 yrs	0.64	1.00E+00	0.93	1.00E+00	0.82	1.00E+00	1.50	1.00E+00	0.92	1.00E+00
40 yrs	0.87	1.00E+00	0.93	1.00E+00	0.82	1.00E+00	1.23	1.00E+00	0.92	1.00E+00

Principal component analysis (PCA) was performed to extract the expression features of the autism spectrum disorder 275 genes across 560 samples.

Supplementary Figure S7. Variance Explained and Cumulative Variance Explained by Principal Components of Pollutant-Disorder Gene Sets for Autism Spectrum Disorder

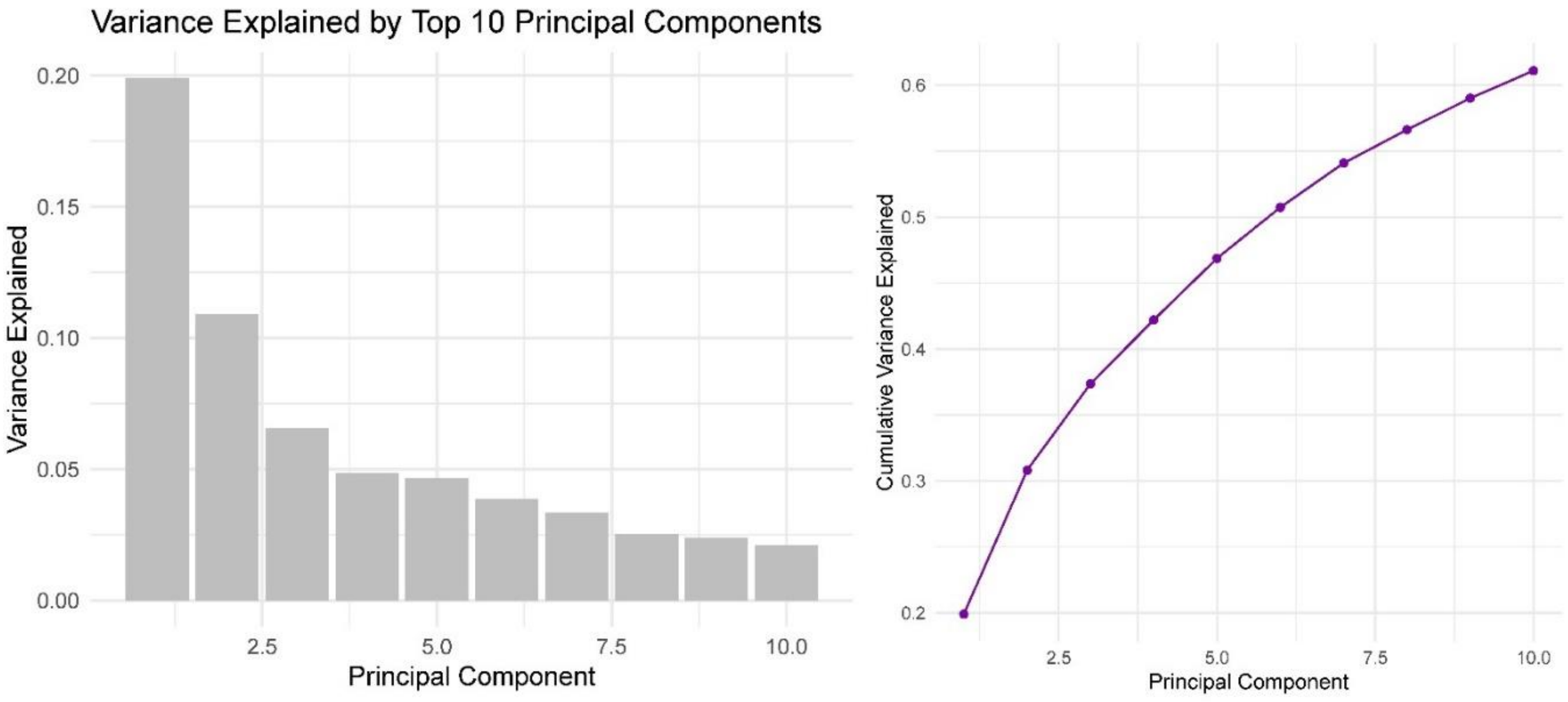

S2.6.2 Temporal Trajectories of Pollutant-Disorder Gene Sets for Autism Spectrum Disorder

Based on the analysis of BrainSpan data by Werling et al., they identified three distinct gene expression trajectories: 6,934 "rising" genes (showing higher expression postnatally), 5,143 "falling" genes (showing higher expression prenatally), and 11,705 "non-transitional" genes (with no statistically significant change in expression).[11] Similarly, we categorized the expression trajectories of the pollutant-disorder gene sets for autism spectrum disorder into three groups: 76 genes with higher expression prenatally, 86 genes with higher expression around the perinatal period, and 113 genes with higher expression postnatally.

Supplementary Table S51. Trajectory Gene Sets for Autism Spectrum Disorder

Cluster	Number of Genes	Gene Sets
Cluster 1	76	ADH1A|ADH5|ADH6|ADSL|AHR|AKR1A1|AKR1C2|ALDH3A2|AOC1|ARSK|B3GAT2|CBS|CEP41|CHAT|CHD2|CHD8|CHST10|CHST11|CIRBP|CNTNAP2|CTTNBP2|CYP27B1|CYP2C9|CYP2R1|CYP3A5|CYP4A11|DIO3|DIP2A|DNAAF2|DNMT1|DNMT3A|DNMT3B|DPP10|FMO1|FOXP1|FOXP2|GABRB3|GNGT1|GPX5|GPX6|IFT74|INSR|IQGAP3|ITGB3|JARID2|KIFC1|LRRN3|MAGEA8|MEF2C|MTHFR|MTR|MTTP|NRXN1|NSUN2|NTSR1|PAPPA2|PARD6B|PTGES|RAI1|RARB|RELN|RSPH4A|SCNN1G|SLC16A2|SLC1A1|SLC25A25|SLC6A4|SOX5|TET1|TET3|TP53I3|TXNRD1|UGT1A1|UGT1A10|UGT1A6|UGT2B10
Cluster 2	113	ABCA2|ABCC3|ABCG2|ACHE|ADH4|AKR1B10|AKR1C3|AKR7A3|ALAD|ALDH1A1|ALDH1A3|ALDH2|ALDH3A1|ALDH3B1|ALDH3B2|ALDH4A1|ALDH5A1|ALOX15|ANKRD11|AQP9|ARSI|ATP1A3|BDNF|CA2|CBR1|CBR3|CES2|CFTR|CGN|CHST6|CLDN3|CRYZ|CYP11A1|CYP11B2|CYP19A1|CYP1A1|CYP1A2|CYP24A1|CYP26B1|CYP27A1|CYP2B6|CYP2C19|CYP2C8|CYP2D6|CYP2F1|CYP2J2|CYP3A7|CYP4A22|CYP4B1|CYP4X1|CYP7A1|DHCR24|DHRS2|DIO2|DLG4|DPP6|DPYD|EN2|EPHX1|EPHX2|FMO3|GAL3ST1|GPX2|GSTA2|GSTM1|GSTM5|GSTO1|HSD3B1|HTRA4|IL1RAPL1|IL1RL1|JAM3|LRRTM3|MET|MPDZ|NOS1|NOS2|NQO1|NQO2|OXTR|PAH|PCDH9|PON3|PRKCZ|PSG1|PSG6|PTCHD1|PTGES2|PTGR1|PTGS1|ROGDI|RYR2|SCN1A|SCNN1A|SHANK3|SLC13A4|SLC22A5|SLC25A20|SLCO1C1|STS|SULT1C2|SULT1C3|SULT2B1|SUOX|SYN1|TFRC|TJP1|TJP2|TPMT|UGT2A3|UNC80|UPP2|USH2A
Cluster 3	86	ABCB1|ADA|ADH1B|ADRB2|AKR7A2|ALDH1L1|ALDH6A1|ALOX12|ALOX12B|AOC2|AOX1|APC|AQP4|ARSG|ATP1B2|AVPR1A|BCHE|CES3|CGNL1|CHST3|CHST8|CIC|COMT|CP|CYP21A2|CYP27C1|CYP2S1|CYP2U1|CYP39A1|DHRS1|DNAH11|DNAH7|DNAI2|DRD4|EBI3|FLT1|FMO2|FMO4|FOLR2|GALNS|GFAP|GSTA3|GSTP1|GUSB|HEY1|HFE|HGF|HSD17B1|ITPR3|JAG1|JAM2|KIF17|LAMC3|LOXL1|LOXL2|LOXL4|LRP1|MIF|NOS3|NOTCH1|NOTCH2|NOTCH3|NOTCH4|NRXN2|PARD3|PON2|PRG2|PROK1|PTGIS|SFSWAP|SLC12A7|SLC16A1|SLC19A1|SLC1A4|SLC2A3|SLC3A2|SLCO2A1|SOX9|SULF1|SULF2|SULT1B1|TPST2|TXNRD2|UGT2B7|VCL|VWF

Supplementary Figure S8. Associations Between First/Second Principal Components and Time in Cluster 1

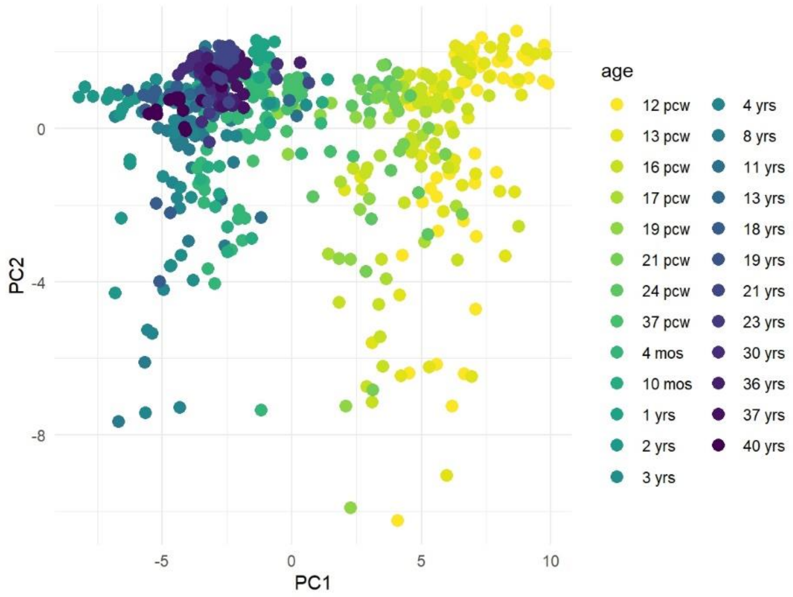

Supplementary Table S52. Significant GO Biological Processes for Genes in Cluster 1, Bonferroni-Corrected P-values < 0.05

Term	Count	%	Genes	Fold Enrichment	Bonferroni P-values
GO:0006805~xenobiotic metabolic process	8	10.5	CYP2C9, UGT1A10, UGT1A1, CYP2R1, FMO1, AHR, CYP3A5, UGT1A6	17.4	2.89E-04
GO:0008210~estrogen metabolic process	5	6.6	UGT2B10, CYP2C9, UGT1A1, CHST10, CYP3A5	41.1	4.98E-03
GO:1903926~cellular response to bisphenol A	3	3.9	DNMT1, SLC1A1, DNMT3A	263.1	3.37E-02
GO:0052695~cellular glucuronidation	4	5.3	UGT2B10, UGT1A10, UGT1A1, UGT1A6	52.6	4.45E-02

Supplementary Figure S9. Associations Between First/Second Principal Components and Time in Cluster 2

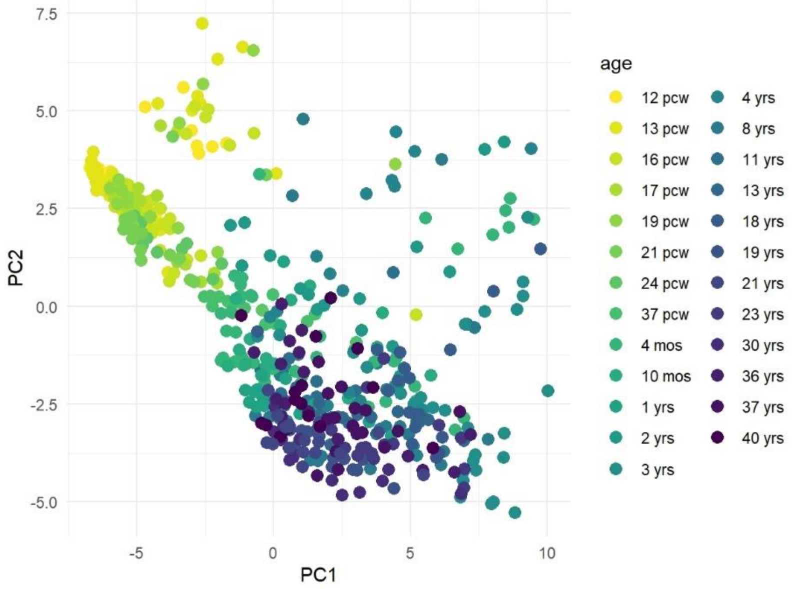

Supplementary Table S53. Significant GO Biological Processes for Genes in Cluster 2, Bonferroni-Corrected P-values < 0.05

Term	Count	%	Genes	Fold Enrichment	Bonferroni P-values
GO:0006805~xenobiotic metabolic process	20	17.7	CYP2J2, ABCC3, NQO1, CBR1, TPMT, EPHX1, CYP2C19, CYP3A7, SULT1C3, ALDH3A1, CYP2C8, CYP26B1, CYP2B6, CYP2D6, GSTA2, CYP1A2, CYP1A1, CYP2F1, CES2, CBR3	29.12	1.37E-19
GO:0042178~xenobiotic catabolic process	10	8.8	CYP2C8, GSTM1, CYP2B6, TPMT, GSTO1, CYP2D6, CYP1A2, NOS1, CYP2C19, CRYZ	76.61	2.82E-12
GO:0019373~epoxygenase P450 pathway	8	7.1	CYP2J2, CYP2C8, CYP2B6, CYP1A2, CYP4A22, CYP1A1, CYP2F1, CYP2C19	67.12	1.45E-08
GO:0042572~retinol metabolic process	10	8.8	ALDH1A3, ADH4, CYP2C8, AKR1B10, CYP2D6, CYP1A2, ALDH1A1, AKR1C3, CYP1A1, CYP3A7	32.63	1.58E-08
GO:0008202~steroid metabolic process	9	8.0	SULT2B1, CYP2C8, CYP2B6, CYP2D6, AKR1C3, CYP1A1, DHCR24, CYP2C19, CYP3A7	31.72	3.10E-07
GO:0006081~cellular aldehyde metabolic process	6	5.3	ALDH3A1, ALDH3B2, ADH4, AKR7A3, ALDH3B1, ALDH1A1	96.11	2.09E-06

GO:0016125~sterol metabolic process	6	5.3	CYP27A1, CYP26B1, CYP11B2, CYP11A1, CYP19A1, CYP7A1	50.34	8.82E-05
GO:0097267~omega-hydroxylase P450 pathway	5	4.4	CYP2C8, CYP1A2, CYP4A22, CYP1A1, CYP2C19	73.42	4.11E-04
GO:0008203~cholesterol metabolic process	8	7.1	SULT1C3, SULT2B1, CYP27A1, CYP24A1, CYP11B2, CYP2D6, CYP11A1, CYP1A2	16.20	5.38E-04
GO:0008210~estrogen metabolic process	6	5.3	CYP2C8, CYP2D6, CYP1A2, CYP1A1, UGT2A3, CYP3A7	33.04	8.31E-04
GO:0042759~long-chain fatty acid biosynthetic process	5	4.4	CYP2C8, CYP2D6, ALOX15, CYP1A2, CYP1A1	41.95	4.76E-03
GO:0002933~lipid hydroxylation	4	3.5	CYP2C8, CYP4A22, CYP1A1, CYP3A7	100.68	5.39E-03
GO:0006066~alcohol metabolic process	4	3.5	ALDH3B2, ADH4, ALDH2, ALDH3B1	100.68	5.39E-03
GO:0034650~cortisol metabolic process	4	3.5	CYP27A1, CYP24A1, CYP11B2, CYP11A1	88.10	8.57E-03
GO:0006700~C21-steroid hormone biosynthetic process	4	3.5	CYP27A1, CYP24A1, CYP11B2, CYP11A1	78.31	1.28E-02
GO:0019371~cyclooxygenase pathway	4	3.5	CBR1, PTGES2, AKR1C3, PTGS1	78.31	1.28E-02
GO:0070989~oxidative demethylation	4	3.5	CYP2C8, CYP2D6, CYP1A2, CYP3A7	70.48	1.81E-02
GO:0006704~glucocorticoid biosynthetic process	4	3.5	CYP27A1, CYP24A1, CYP11B2, CYP11A1	70.48	1.81E-02

Supplementary Figure S10. Associations Between First/Second Principal Components and Time in Cluster 3

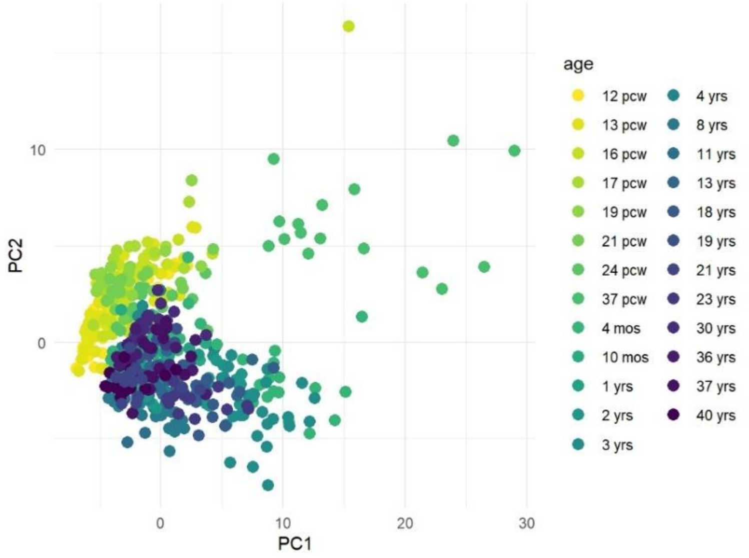

Supplementary Table S54. Significant GO Biological Processes for Genes in Cluster 3, Bonferroni-Corrected P-values < 0.05

Term	Count	%	Genes	Fold Enrichment	Bonferroni P-values
GO:0006805~xenobiotic metabolic process	15	17.4	BCHE, AOC2, ABCB1, GSTP1, FMO2, FMO4, SULT1B1, CYP2U1, CYP21A2, GSTA3, CYP2S1, AOX1, CES3, UGT2B7, ADA	29.12	1.21E-13
GO:0150104~transport across blood-brain barrier	8	9.3	SLC16A1, ABCB1, LRP1, SLC2A3, SLC1A4, ATP1B2, AVPR1A, SLC19A1	22.37	6.87E-05
GO:0003203~endocardial cushion morphogenesis	5	5.8	NOTCH1, APC, HEY1, NOS3, SOX9	61.82	1.17E-03
GO:0003184~pulmonary valve morphogenesis	5	5.8	NOTCH2, NOTCH1, JAG1, HEY1, NOS3	55.94	1.80E-03
GO:0070168~negative regulation of biomineral tissue development	4	4.7	NOTCH1, HEY1, NOS3, SOX9	104.41	6.45E-03
GO:0007219~Notch signaling pathway	7	8.1	NOTCH2, NOTCH3, NOTCH1, JAG1, HEY1, NOTCH4, SOX9	13.37	1.44E-02
GO:0003180~aortic valve morphogenesis	5	5.8	NOTCH1, JAG1, HEY1, NOS3, SOX9	31.75	1.87E-02
GO:0006082~organic acid metabolic process	4	4.7	CYP2U1, CYP21A2, FMO2, FMO4	72.29	2.15E-02

GO:0060411~cardiac septum morphogenesis	4	4.7	DNAH11, NOTCH1, JAG1, HEY1	67.12	2.73E-02

S2.6.3 Temporal Expression of Pollutant-Disorder Gene Sets for Intellectual Disability

The gene expression matrix was filtered based on the mapping of intellectual disability pollutant-disorder gene sets to the BrainSpan gene expression data. Five genes—"ADRA2B," "GRIN2B," "H3-4," "H4C3," and "HYCC1"—were excluded due to their absence in the expression data, narrowing the analysis to 93 genes.

Supplementary Table S55. Associations Between Intellectual Disability Gene Sets and High Expressed Gene Groups

Time Point	All Pollutants		Air Pollutants		Toxic and Trace Elements		Pesticides and Related Compounds		Synthetic Organic Chemicals	
	Odds Ratio	P-Adjusted	Odds Ratio	P-Adjusted	Odds Ratio	P-Adjusted	Odds Ratio	P-Adjusted	Odds Ratio	P-Adjusted
12 pcw	1.08	1.00E+00	0.50	1.00E+00	1.49	1.00E+00	0.68	1.00E+00	0.34	1.00E+00
13 pcw	2.57	1.66E-01	1.58	1.00E+00	3.25	1.43E-01	3.05	1.00E+00	2.24	1.00E+00
16 pcw	2.05	1.00E+00	1.58	1.00E+00	1.90	1.00E+00	3.05	1.00E+00	2.24	1.00E+00
17 pcw	1.55	1.00E+00	1.58	1.00E+00	0.72	1.00E+00	0.68	1.00E+00	2.24	1.00E+00
19 pcw	0.85	1.00E+00	0.50	1.00E+00	1.10	1.00E+00	0.68	1.00E+00	1.06	1.00E+00
21 pcw	4.00	3.42E-04	5.74	2.57E-03	3.74	3.76E-02	6.08	1.13E-02	3.59	4.87E-02
24 pcw	1.55	1.00E+00	1.58	1.00E+00	0.72	1.00E+00	3.05	1.00E+00	1.06	1.00E+00
37 pcw	0.85	1.00E+00	1.58	1.00E+00	1.10	1.00E+00	0.68	1.00E+00	0.69	1.00E+00
4 mos	2.31	6.50E-01	3.47	3.04E-01	2.34	1.00E+00	3.05	1.00E+00	1.83	1.00E+00
10 mos	0.85	1.00E+00	1.03	1.00E+00	1.49	1.00E+00	0.68	1.00E+00	0.34	1.00E+00
1 yrs	4.00	3.37E-04	4.94	1.45E-02	4.26	8.69E-03	3.98	3.36E-01	2.67	5.70E-01
2 yrs	0.42	1.00E+00	1.03	1.00E+00	0.35	1.00E+00	1.41	1.00E+00	0.69	1.00E+00
3 yrs	1.08	1.00E+00	1.58	1.00E+00	0.35	1.00E+00	0.68	1.00E+00	1.83	1.00E+00
4 yrs	0.21	1.00E+00	0.00	1.00E+00	0.00	1.00E+00	0.68	1.00E+00	0.00	1.00E+00
8 yrs	0.63	1.00E+00	0.50	1.00E+00	0.35	1.00E+00	0.68	1.00E+00	0.69	1.00E+00
11 yrs	0.63	1.00E+00	0.00	1.00E+00	1.10	1.00E+00	0.00	1.00E+00	0.00	1.00E+00
13 yrs	0.63	1.00E+00	1.58	1.00E+00	0.35	1.00E+00	0.68	1.00E+00	0.69	1.00E+00
18 yrs	0.21	1.00E+00	0.50	1.00E+00	0.00	1.00E+00	0.00	1.00E+00	0.34	1.00E+00
19 yrs	0.42	1.00E+00	0.50	1.00E+00	0.72	1.00E+00	0.00	1.00E+00	0.34	1.00E+00
21 yrs	1.79	1.00E+00	1.03	1.00E+00	1.49	1.00E+00	0.68	1.00E+00	1.44	1.00E+00

23 yrs	0.42	1.00E+00	1.03	1.00E+00	0.00	1.00E+00	0.00	1.00E+00	0.69	1.00E+00
30 yrs	0.85	1.00E+00	1.58	1.00E+00	1.49	1.00E+00	0.68	1.00E+00	1.06	1.00E+00
36 yrs	1.08	1.00E+00	0.50	1.00E+00	0.35	1.00E+00	0.68	1.00E+00	1.44	1.00E+00
37 yrs	0.63	1.00E+00	0.50	1.00E+00	0.35	1.00E+00	0.68	1.00E+00	0.69	1.00E+00
40 yrs	0.00	3.75E-01	0.00	1.00E+00	0.00	1.00E+00	0.00	1.00E+00	0.00	1.00E+00

Supplementary Table S56. Associations Between Intellectual Disability Gene Sets and Low Expressed Gene Groups

Time Point	All Pollutants		Air Pollutants		Toxic and Trace Elements		Pesticides and Related Compounds		Synthetic Organic Chemicals	
	Odds Ratio	P-Adjusted	Odds Ratio	P-Adjusted	Odds Ratio	P-Adjusted	Odds Ratio	P-Adjusted	Odds Ratio	P-Adjusted
12 pcw	0.42	1.00E+00	0.50	1.00E+00	0.72	1.00E+00	0.68	1.00E+00	0.34	1.00E+00
13 pcw	0.42	1.00E+00	1.03	1.00E+00	0.72	1.00E+00	0.68	1.00E+00	0.00	1.00E+00
16 pcw	0.85	1.00E+00	1.03	1.00E+00	1.49	1.00E+00	0.68	1.00E+00	0.00	1.00E+00
17 pcw	1.08	1.00E+00	2.18	1.00E+00	1.10	1.00E+00	0.68	1.00E+00	0.69	1.00E+00
19 pcw	1.55	1.00E+00	1.03	1.00E+00	1.90	1.00E+00	0.00	1.00E+00	0.69	1.00E+00
21 pcw	0.63	1.00E+00	0.50	1.00E+00	0.35	1.00E+00	0.00	1.00E+00	1.06	1.00E+00
24 pcw	0.63	1.00E+00	0.50	1.00E+00	0.72	1.00E+00	0.68	1.00E+00	0.69	1.00E+00
37 pcw	1.55	1.00E+00	1.58	1.00E+00	2.33	1.00E+00	3.05	1.00E+00	1.83	1.00E+00
4 mos	1.31	1.00E+00	1.58	1.00E+00	1.10	1.00E+00	1.41	1.00E+00	1.44	1.00E+00
10 mos	0.42	1.00E+00	0.00	1.00E+00	0.35	1.00E+00	0.00	1.00E+00	0.34	1.00E+00
1 yrs	0.63	1.00E+00	0.00	1.00E+00	1.10	1.00E+00	0.68	1.00E+00	0.34	1.00E+00
2 yrs	1.31	1.00E+00	1.03	1.00E+00	2.33	1.00E+00	0.00	1.00E+00	1.06	1.00E+00
3 yrs	1.55	1.00E+00	1.03	1.00E+00	1.49	1.00E+00	1.41	1.00E+00	1.83	1.00E+00
4 yrs	4.96	3.42E-06	4.93	1.46E-02	4.79	1.82E-03	6.08	1.13E-02	5.12	5.01E-04
8 yrs	3.40	5.16E-03	4.18	7.23E-02	1.90	1.00E+00	3.97	3.38E-01	3.12	1.78E-01
11 yrs	1.79	1.00E+00	1.58	1.00E+00	1.49	1.00E+00	0.00	1.00E+00	2.67	5.73E-01
13 yrs	0.21	1.00E+00	0.00	1.00E+00	0.35	1.00E+00	0.00	1.00E+00	0.34	1.00E+00
18 yrs	1.31	1.00E+00	1.03	1.00E+00	1.49	1.00E+00	1.41	1.00E+00	1.44	1.00E+00

19 yrs	0.42	1.00E+00	0.00	1.00E+00	0.35	1.00E+00	0.00	1.00E+00	0.69	1.00E+00
21 yrs	1.08	1.00E+00	0.50	1.00E+00	0.72	1.00E+00	0.00	1.00E+00	1.44	1.00E+00
23 yrs	0.63	1.00E+00	0.50	1.00E+00	0.72	1.00E+00	0.68	1.00E+00	1.06	1.00E+00
30 yrs	1.79	1.00E+00	1.03	1.00E+00	1.49	1.00E+00	1.41	1.00E+00	1.83	1.00E+00
36 yrs	0.42	1.00E+00	0.50	1.00E+00	0.00	1.00E+00	0.00	1.00E+00	0.34	1.00E+00
37 yrs	0.85	1.00E+00	0.00	1.00E+00	0.72	1.00E+00	0.68	1.00E+00	1.06	1.00E+00
40 yrs	2.84	5.68E-02	2.18	1.00E+00	2.33	1.00E+00	3.05	1.00E+00	3.12	1.78E-01

Supplementary Figure S11. Associations Between Intellectual Disability Gene Sets and High/Low Expressed Gene Groups

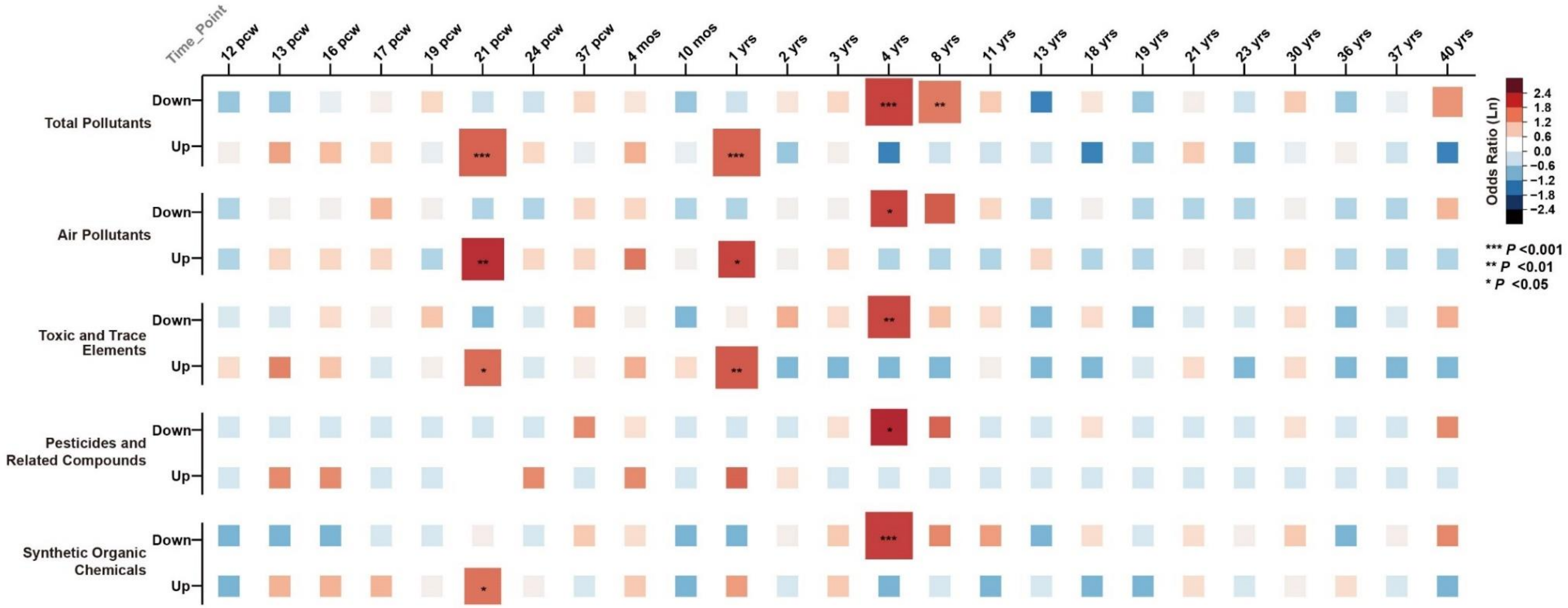

Principal component analysis (PCA) was performed to extract the expression features of the intellectual disability 93 genes across 560 samples.

Supplementary Figure S12. Associations Between First/Second Principal Components and Time in Intellectual Disability Gene Sets

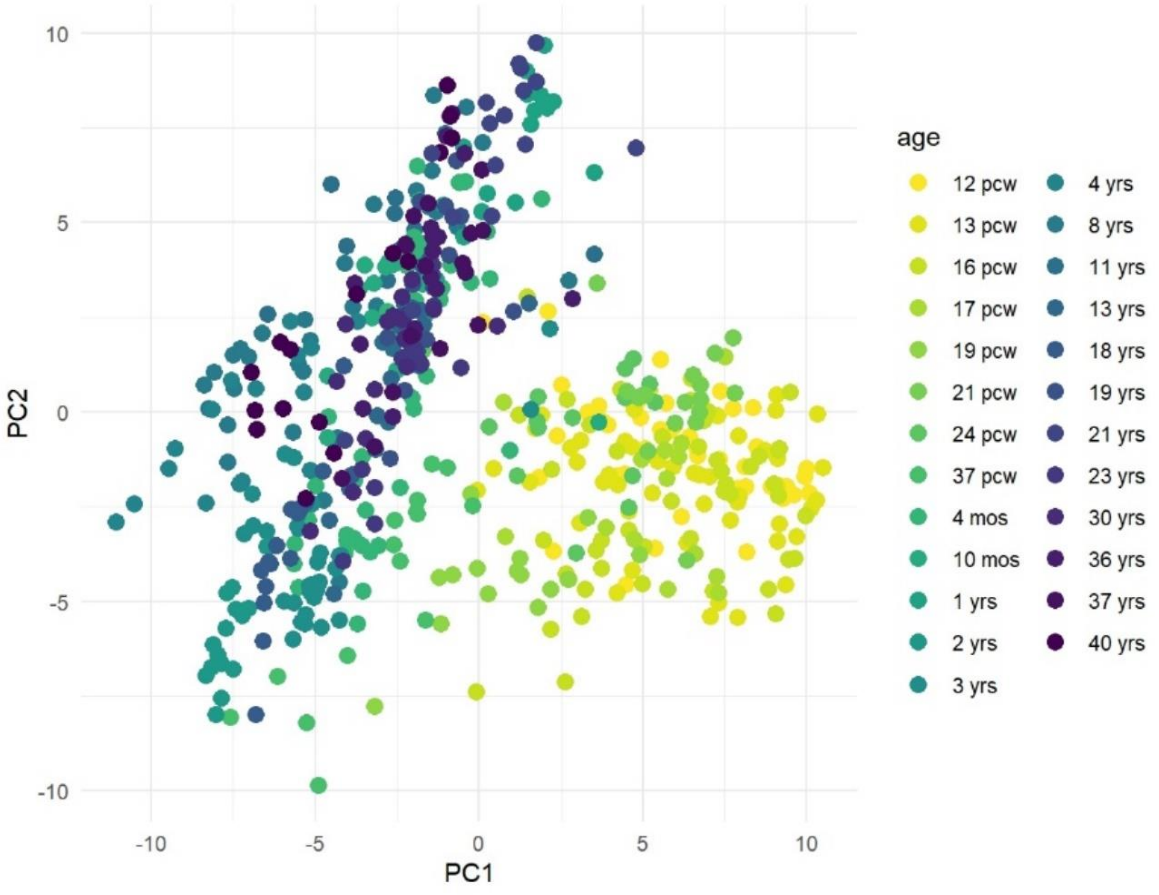

Supplementary Figure S13. Variance Explained and Cumulative Variance Explained by Principal Components of Pollutant-Disorder Gene Sets for Intellectual Disability

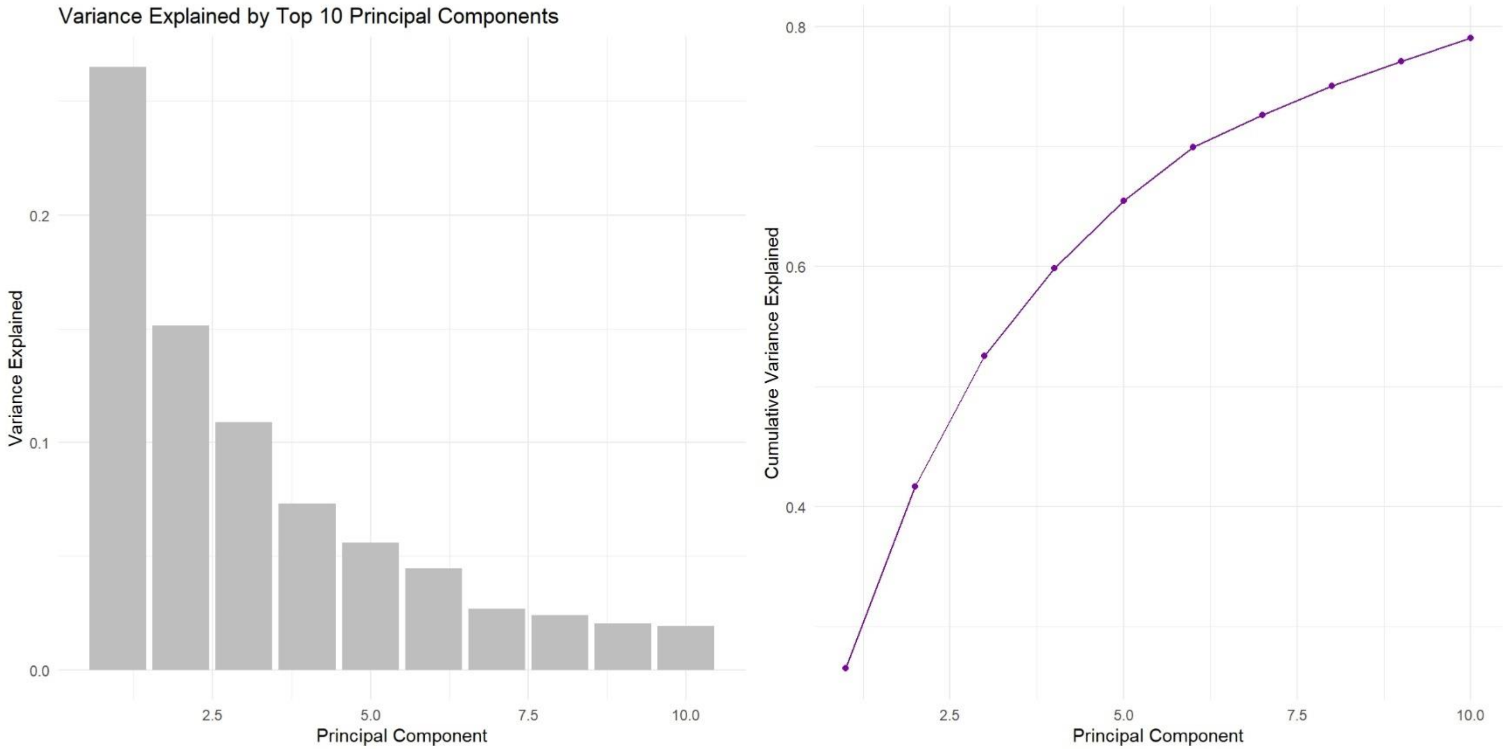

S2.6.4 Temporal Trajectories of Pollutant-Disorder Gene Sets for Intellectual Disability

Based on the analysis of BrainSpan data by Werling et al., they identified three distinct gene expression trajectories: "rising" genes, "falling" genes, and "non-transitional" genes.[11] Similarly, we categorized the expression trajectories of the pollutant-disorder gene sets for intellectual disability into three groups: 44 genes with higher expression prenatally, 30 genes with higher expression around the perinatal period, and 19 genes with higher expression postnatally.

Supplementary Table S57. Trajectory Gene Sets for Intellectual Disability

Cluster	Number of Genes	Gene Sets
Cluster 1	19	ADK|ALDH5A1|BBS7|BDNF|CACNA1G|CALCA|FGFR2|FMR1|KCNA2|NF1|PDHX|PRKCG|RABL6|RGS7|SCN1A|SCN8A|SHANK3|SLC4A10|TH
Cluster 2	30	APC|CAPN10|CIC|COL18A1|COQ5|DISC1|DOCK8|FOLR1|FRY|GAMT|GNAS|HDAC4|HEXA|LAMA1|MCC|METTL23|MFSD2A|NAGLU|NTF4|PAX6|PEX6|PTCHD1|RALGDS|SHANK2|SLC2A1|SLC31A1|SYNGAP1|TBCD|TSEN54|WDR45B
Cluster 3	44	AP4E1|ARL14EP|CA8|CASP2|CCBE1|CCNA2|CHD8|CHL1|DCC|DNMT3A|DYNC1H1|EEF1B2|ENTPD1|FASN|GON4L|INPP4A|KDM5A|KDM5C|KDM6B|KIF7|L2HGDH|LARP7|LETM1|MECP2|MED13L|MEF2C|NAA15|NRXN1|NSD1|PARP1|PHIP|POLR3B|PRKRA|PTEN|RAI1|SC5D|SCAPER|SETBP1|SRGAP3|TAF2|TMCO1|WDR62|YY1|ZBTB40

Supplementary Figure S14. Temporal Trajectories of Intellectual Disability Clusters

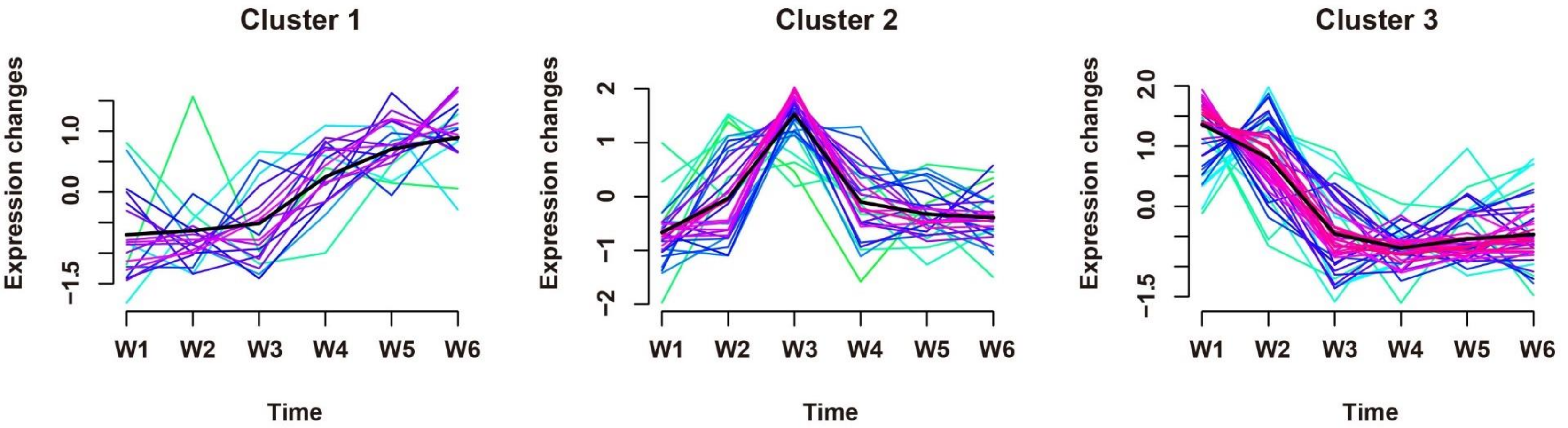

Supplementary Figure S15. Associations Between First/Second Principal Components and Time in Cluster 1

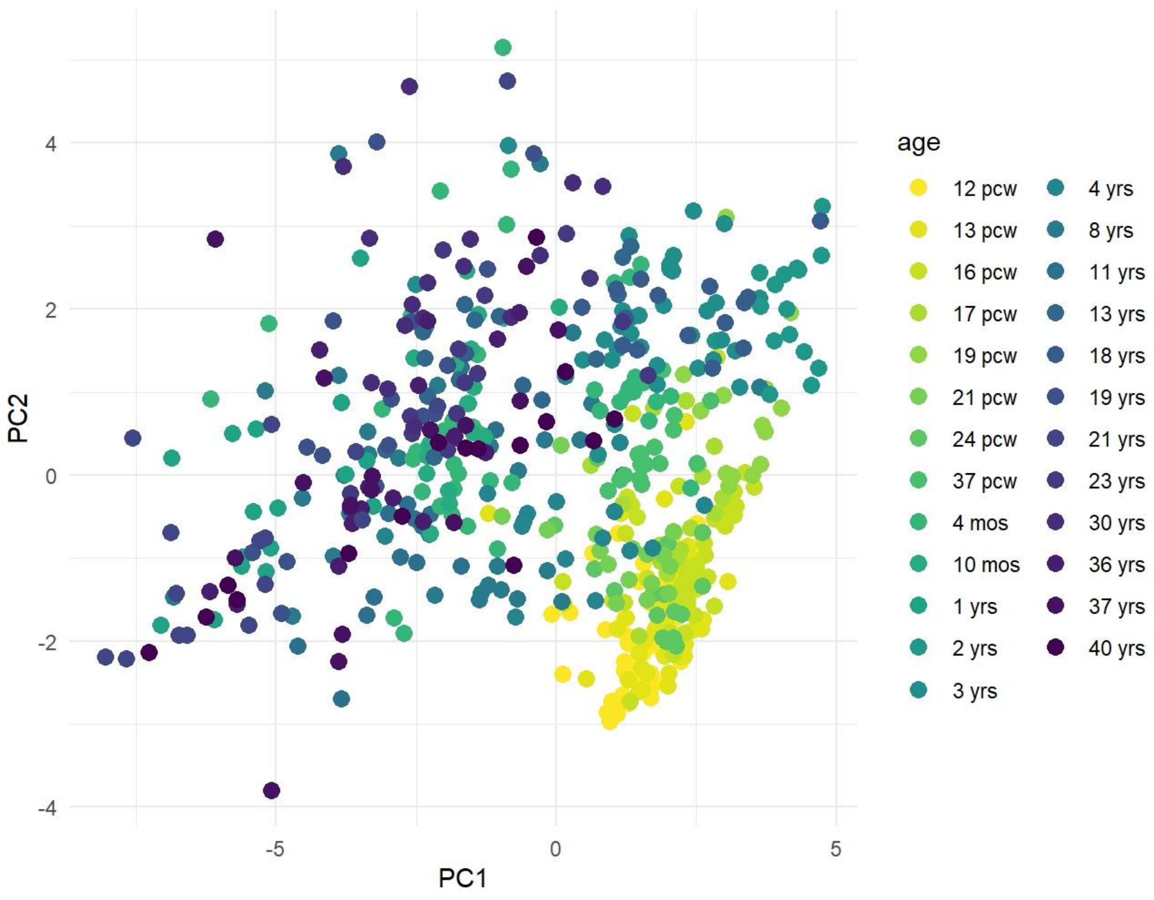

PC2
PC1
4
2
0
-2
-4
-5
0
5
age
12 pcw
13 pcw
16 pcw
17 pcw
19 pcw
21 pcw
24 pcw
37 pcw
4 mos
10 mos
1 yrs
2 yrs
3 yrs
4 yrs
8 yrs
11 yrs
13 yrs
18 yrs
19 yrs
21 yrs
23 yrs
30 yrs
36 yrs
37 yrs
40 yrs

Supplementary Figure S16. Variance Explained and Cumulative Variance Explained by Principal Components of Cluster 1

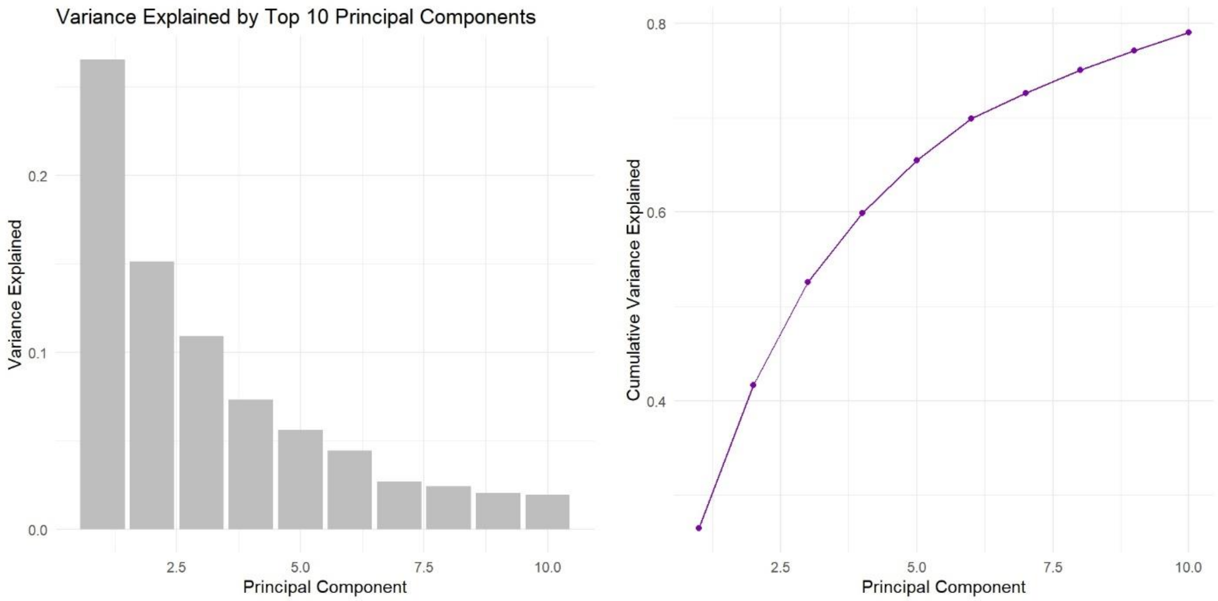

Supplementary Table S58. Significant GO Biological Processes for Genes in Cluster 1, Bonferroni-Corrected P-values < 0.05

Term	Count	%	Genes	Fold Enrichment	Bonferroni P-values
GO:0086010~membrane depolarization during action potential	3	15.8	SCN8A, CACNA1G, SCN1A	205.56	3.95E-02

Supplementary Figure S17. Associations Between First/Second Principal Components and Time in Cluster 2

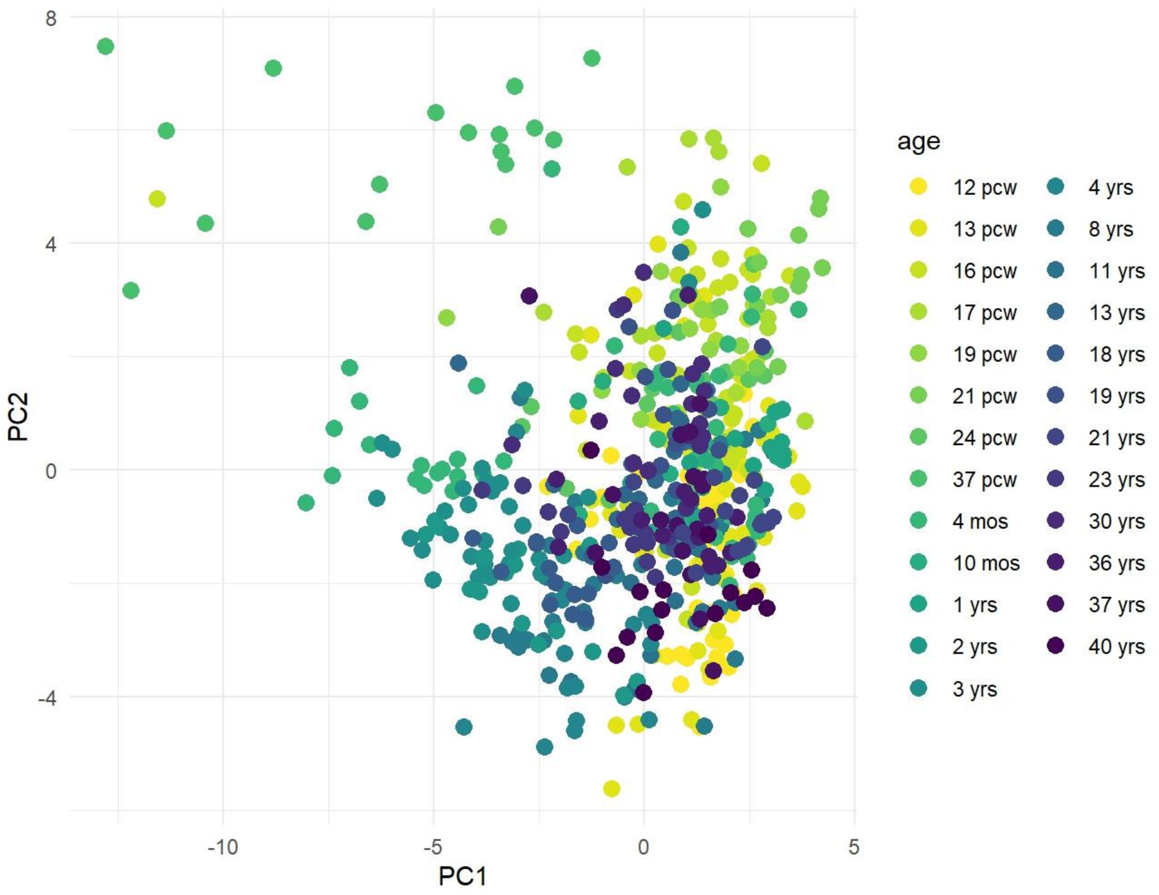

Supplementary Figure S18. Variance Explained and Cumulative Variance Explained by Principal Components of Cluster 2

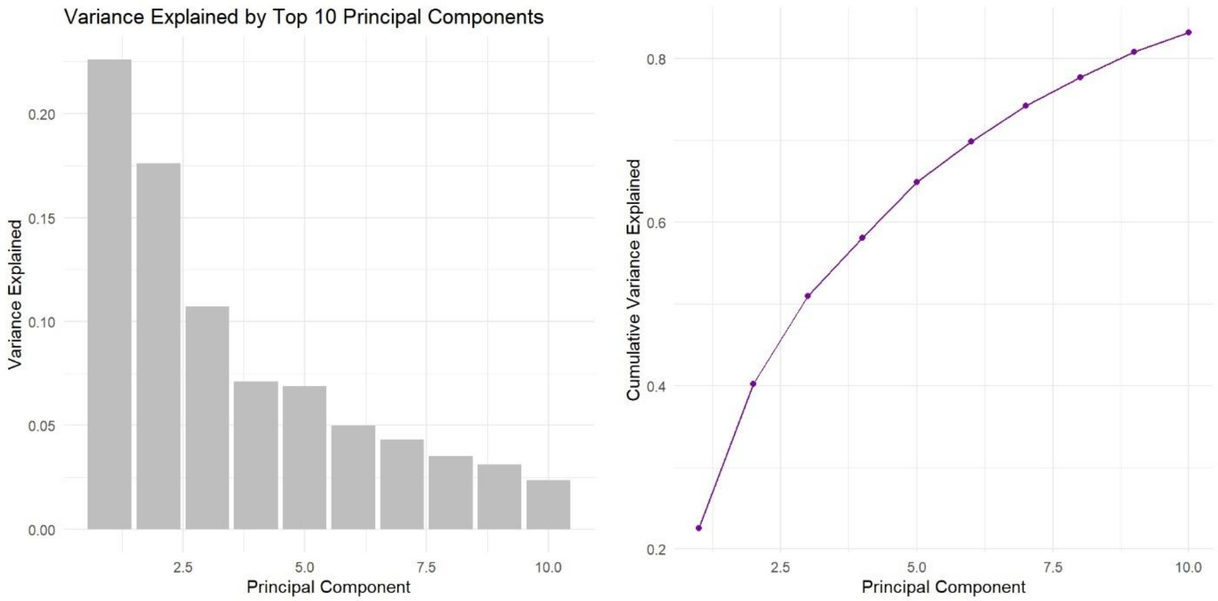

Supplementary Table S59. Significant GO Biological Processes for Genes in Cluster 2, Bonferroni-Corrected P-values < 0.05

Term	Count	%	Genes	Fold Enrichment	Bonferroni P-values
GO:0050890~cognition	4	13.3	METTL23, PTCHD1, GNAS, MFSD2A	47.84	3.00E-02

Supplementary Figure S19. Associations Between First/Second Principal Components and Time in Cluster 3

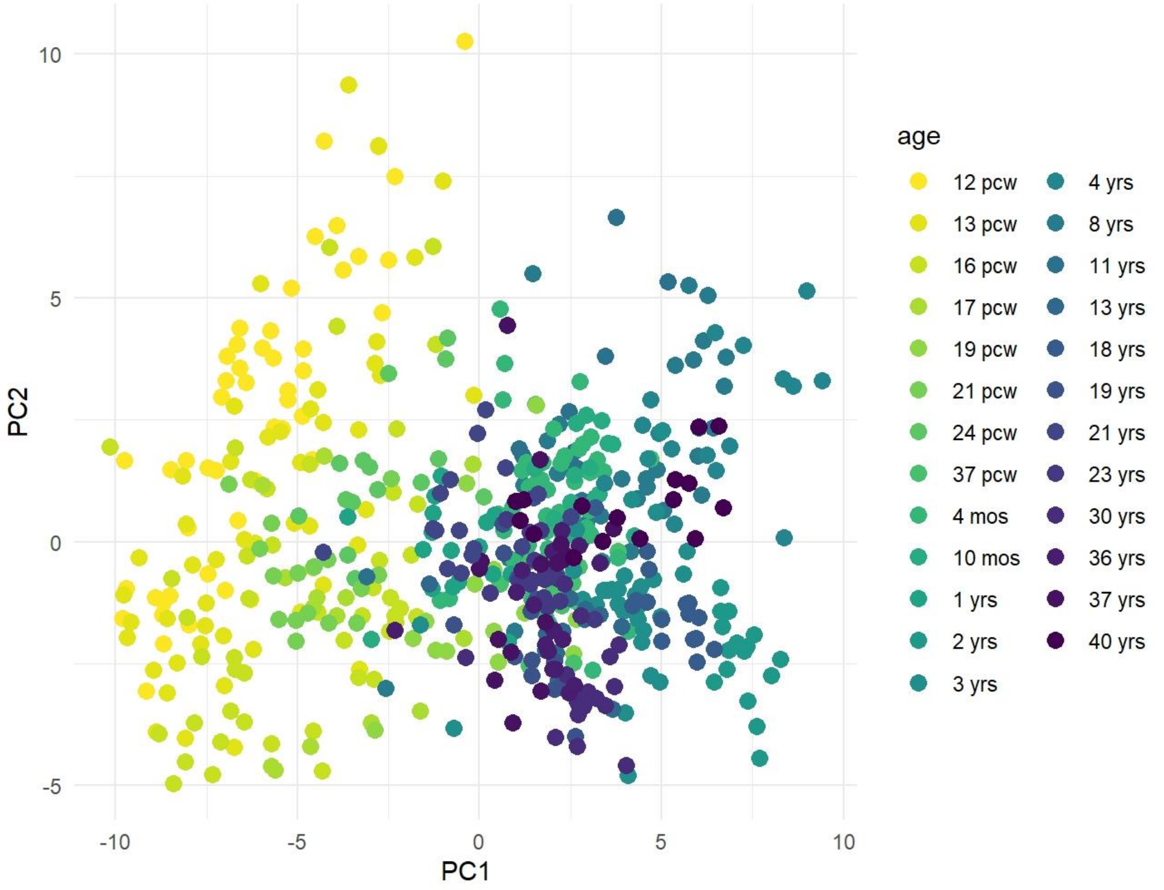

Supplementary Figure S20. Variance Explained and Cumulative Variance Explained by Principal Components of Cluster 3

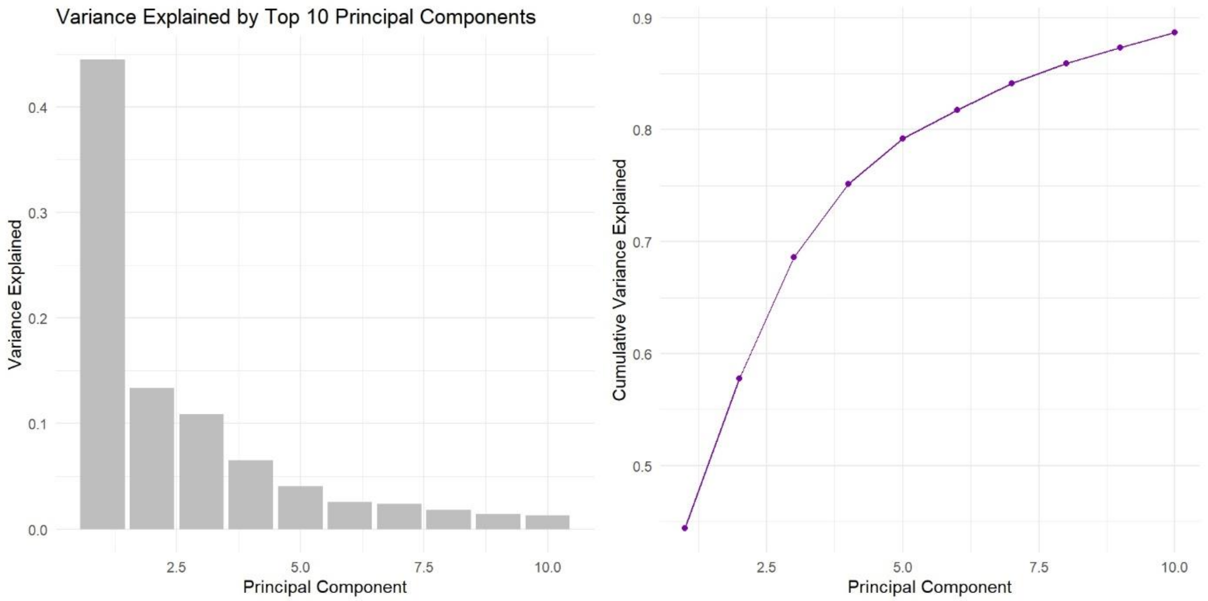

Supplementary Table S60. Significant GO Biological Processes for Genes in Cluster 3, Bonferroni-Corrected P-values < 0.05

Term	Count	%	Genes	Fold Enrichment	Bonferroni P-values
GO:0045893~positive regulation of DNA-templated transcription	10	22.7	KDM5A, YY1, CCNA2, RAI1, MEF2C, CHD8, NSD1, PHIP, NAA15, TAF2	6.44	8.52E-03
GO:0006338~chromatin remodeling	7	15.9	KDM5A, YY1, KDM6B, KDM5C, SETBP1, CHD8, NSD1	9.37	4.48E-02

Supplemental References

1 Davis, A. P. *et al.* Comparative toxicogenomics database's 20th anniversary: update 2025. *Nucleic Acids Res* (2024). https://doi.org/10.1093/nar/gkae883

2 Davis, A. P. *et al.* Generating Gene Ontology-Disease Inferences to Explore Mechanisms of Human Disease at the Comparative Toxicogenomics Database. *PLoS One* **11**, e0155530 (2016). https://doi.org/10.1371/journal.pone.0155530

3 Gao, X., Zheng, X., Wang, X., Li, Z. & Yang, L. Environmental pollutant exposure and adverse neurodevelopmental outcomes: An umbrella review and evidence grading of meta-analyses. *J Hazard Mater* **491**, 137832 (2025). https://doi.org/10.1016/j.jhazmat.2025.137832

4 Vrijheid, M., Casas, M., Gascon, M., Valvi, D. & Nieuwenhuijsen, M. Environmental pollutants and child health—A review of recent concerns. *International Journal of Hygiene and Environmental Health* **219**, 331-342 (2016). https://doi.org/https://doi.org/10.1016/j.ijheh.2016.05.001

5 Naidu, R. *et al.* Chemical pollution: A growing peril and potential catastrophic risk to humanity. *Environment International* **156**, 106616 (2021). https://doi.org/https://doi.org/10.1016/j.envint.2021.106616

6 Adeola, F. O. in *Handbook of Global Health* (eds Ilona Kickbusch, Detlev Ganten, & Matshidiso Moeti) 2227-2256 (Springer International Publishing, 2021).

7 Li, M. *et al.* Integrative functional genomic analysis of human brain development and neuropsychiatric risks. *Science* **362**, eaat7615 (2018). https://doi.org/doi:10.1126/science.aat7615

8 Pei, G. *et al.* Gene expression imputation and cell-type deconvolution in human brain with spatiotemporal precision and its implications for brain-related disorders. *Genome Res* **31**, 146-158 (2021). https://doi.org/10.1101/gr.265769.120

9 Ritchie, M. E. *et al.* limma powers differential expression analyses for RNA-sequencing and microarray studies. *Nucleic Acids Res* **43**, e47 (2015). https://doi.org/10.1093/nar/gkv007

10 Kumar, L. & M, E. F. Mfuzz: a software package for soft clustering of microarray data. *Bioinformation* **2**, 5-7 (2007). https://doi.org/10.6026/97320630002005

11 Werling, D. M. *et al.* Whole-Genome and RNA Sequencing Reveal Variation and Transcriptomic Coordination in the Developing Human Prefrontal Cortex. *Cell Reports* **31**, 107489 (2020). https://doi.org/https://doi.org/10.1016/j.celrep.2020.03.053
